# Supplementary material for: Housing, neighbourhood and sociodemographic associations with adult levels of physical activity and adiposity: baseline findings from the ENABLE London study
Source: BMJ Open. 2018 Aug 17;8(8):e021257. doi: 10.1136/bmjopen-2017-021257 (PMC6104748; doi:10.1136/bmjopen-2017-021257)
Supplement: Supplementary file 1 [file bmjopen-2017-021257supp001.docx]

**Supplementary Table 1: Questionnaire items included in the factor analysis on perceptions of the neighbourhood**

| **Perceptions of neighbourhood crime items** |
| --- |
| There is a lot of crime in my neighbourhood. |
| The level of crime in my neighbourhood makes it unsafe to walk on the streets at night. |
| There are threatening groups of young people in my neighbourhood. |
| The level of crime in my neighbourhood makes it unsafe to walk on the streets during the day. |
| Vandalism, graffiti or deliberate damage to property is a problem in my local area. |
| **Perceptions of neighbourhood quality items** |
| I enjoy walking in my neighbourhood. |
| This area is a place I enjoy living in. |
| My neighbourhood is attractive to look at (e.g. there are attractive buildings, green space. Landscaping views). |
| This area has good leisure things for people like myself, leisure centres or community centres for example. |
| You often see people out on walks or riding their bicycles in my neighbourhood. |
| This area has good local transport. |
| **Additional items included in the factor analysis with factor loadings below 0.4 and were therefore not included in the solution** |
| My neighbourhood is generally free from litter. |
| There is too much traffic in my neighbourhood. |
| Our neighbourhood streets have good lighting at night. |
|  |

Participants were asked to select a response from the following for all questionnaire items stated in the table: Strongly agree, Agree, Neither agree nor disagree, Disagree, Strongly disagree

**Supplementary Table 2: Participant characteristics for 1240 adults with measurements of adiposity at baseline**

|  | Housing sector | | | | | |  | |  |
| --- | --- | --- | --- | --- | --- | --- | --- | --- | --- |
|  | Social | | Intermediate | | Market rent | | Total | |  |
|  | (n = 512) | | (n = 503) | | (n = 225) | | (N = 1240) | | p (X^2^) |
| **Sex** |  |  |  |  |  |  |  |  |  |
| Male | 137 | (26.8%) | 259 | (51.5%) | 126 | (56.0%) | 522 | (42.1%) |  |
| Female | 375 | (73.2%) | 244 | (48.5%) | 99 | (44.0%) | 718 | (57.9%) | <0.0001 |
| **Age group** |  |  |  |  |  |  |  |  |  |
| 16-24 | 107 | (20.9%) | 92 | (18.3%) | 70 | (31.1%) | 269 | (21.7%) |  |
| 25-34 | 129 | (25.2%) | 291 | (57.9%) | 111 | (49.3%) | 531 | (42.8%) |  |
| 35-49 | 233 | (45.5%) | 102 | (20.3%) | 23 | (10.2%) | 358 | (28.9%) |  |
| 50+ | 43 | (8.4%) | 18 | (3.6%) | 21 | (9.3%) | 82 | (6.6%) | <0.0001 |
| **Ethnic group** |  |  |  |  |  |  |  |  |  |
| White | 96 | (18.8%) | 342 | (68.0%) | 157 | (69.8%) | 595 | (48.0%) |  |
| Black | 245 | (47.9%) | 53 | (10.5%) | 16 | (7.1%) | 314 | (25.3%) |  |
| Asian | 107 | (20.9%) | 75 | (14.9%) | 28 | (12.4%) | 210 | (16.9%) |  |
| Mixed/Other | 64 | (12.5%) | 33 | (6.6%) | 24 | (10.7%) | 121 | (9.8%) | <0.0001 |
| **NS-SEC*** |  |  |  |  |  |  |  |  |  |
| Higher Managerial / Professional | 60 | (11.9%) | 357 | (71.4%) | 150 | (66.7%) | 567 | (46.1%) |  |
| Intermediate Occupations | 62 | (12.3%) | 77 | (15.4%) | 38 | (16.9%) | 177 | (14.4%) |  |
| Routine / Manual | 125 | (24.8%) | 34 | (6.8%) | 10 | (4.4%) | 169 | (13.7%) |  |
| Economically inactive | 258 | (51.1%) | 32 | (6.4%) | 27 | (12.0%) | 317 | (25.8%) | <0.0001 |
| **Limiting illness** |  |  |  |  |  |  |  |  |  |
| Yes | 102 | (19.9%) | 40 | (8.0%) | 11 | (4.9%) | 153 | (12.3%) |  |
| No | 410 | (80.1%) | 463 | (92.0%) | 214 | (95.1%) | 1087 | (87.7%) | <0.0001 |

p (X^2^): p-value for Chi-squared test

* 10 responses missing for NS-SEC group

**Supplementary Table 3: Mean levels of adiposity and physical activity by participant characteristics**

|  |  | Mean/Geometric mean* levels adiposity and physical activity (95% confidence intervals) | | | | | | | | | |
| --- | --- | --- | --- | --- | --- | --- | --- | --- | --- | --- | --- |
|  | n | BMI (kg/m^2^)* | | Fat mass % | | Daily steps† | | Daily minutes of MVPA† | | Daily minutes of MVPA in ≥10 minute bouts† | |
| **Sex** |  |  |  |  |  |  |  |  |  |  |  |
| Male | 522 | 25.4 | (25.0, 25.8) | 20.4 | (19.7, 21.0) | 9279 | (8991, 9568) | 64.8 | (62.6, 67.1) | 22.8 | (21.1, 24.4) |
| Female | 718 | 25.1 | (24.8, 25.5) | 31.5 | (30.9, 32.0) | 8709 | (8464, 8954) | 55.6 | (53.6, 57.5) | 18.7 | (17.3, 20.1) |
| **Age group** |  |  |  |  |  |  |  |  |  |  |  |
| Age 16-24 | 269 | 23.5 | (23.0, 24.0) | 23.0 | (22.1, 23.9) | 8534 | (8136, 8932) | 57.0 | (53.9, 60.2) | 20.5 | (18.2, 22.8) |
| Age 25-34 | 531 | 25.0 | (24.6, 25.3) | 26.2 | (25.5, 26.8) | 9035 | (8744, 9326) | 61.1 | (58.8, 63.3) | 21.4 | (19.7, 23.1) |
| Age 35-49 | 358 | 26.6 | (26.1, 27.1) | 29.3 | (28.5, 30.1) | 9232 | (8879, 9585) | 60.9 | (58.1, 63.6) | 19.4 | (17.3, 21.4) |
| Age 50+ | 82 | 27.6 | (26.6, 28.7) | 32.2 | (30.5, 33.8) | 8525 | (7800, 9249) | 51.1 | (45.4, 56.7) | 18.4 | (14.2, 22.7) |
| **Ethnic group** |  |  |  |  |  |  |  |  |  |  |  |
| White | 595 | 24.9 | (24.5, 25.2) | 25.8 | (25.1, 26.4) | 9491 | (9203, 9779) | 63.6 | (61.3, 65.8) | 23.7 | (22.0, 25.4) |
| Black | 314 | 26.4 | (25.8, 27.0) | 29.4 | (28.5, 30.3) | 8375 | (7961, 8789) | 56.2 | (52.9, 59.4) | 17.1 | (14.7, 19.6) |
| Asian | 210 | 24.8 | (24.2, 25.4) | 25.8 | (24.8, 26.8) | 8082 | (7608, 8556) | 52.1 | (48.4, 55.8) | 15.6 | (12.8, 18.4) |
| Other/Mixed | 121 | 25.2 | (24.4, 26.0) | 26.8 | (25.5, 28.1) | 9060 | (8465, 9656) | 59.0 | (54.3, 63.7) | 19.8 | (16.3, 23.3) |
| **Limiting illness** | |  |  |  |  |  |  |  |  |  |  |
| No | 1087 | 25.1 | (24.9, 25.4) | 26.6 | (26.1, 27.0) | 9077 | (8877, 9277) | 60.1 | (58.6, 61.7) | 20.8 | (19.6, 21.9) |
| Yes | 153 | 26.2 | (25.5, 27.0) | 28.2 | (27.0, 29.4) | 7996 | (7447, 8545) | 54.4 | (50.1, 58.7) | 18.0 | (14.8, 21.1) |
| **Housing sector** | |  |  |  |  |  |  |  |  |  |  |
| Social | 512 | 26.0 | (25.6, 26.5) | 28.4 | (27.6, 29.1) | 8298 | (7953, 8642) | 54.6 | (51.8, 57.3) | 16.0 | (14.0, 18.1) |
| Intermediate | 503 | 24.8 | (24.4, 25.2) | 25.7 | (25.0, 26.4) | 9422 | (9110, 9735) | 62.1 | (59.6, 64.5) | 22.5 | (20.6, 24.4) |
| Market-rent | 225 | 24.6 | (24.0, 25.2) | 25.5 | (24.5, 26.5) | 9318 | (8863, 9773) | 64.3 | (60.8, 67.9) | 25.3 | (22.6, 28.1) |

* Geometric means are presented for BMI

All means/geometric means are adjusted for sex, age group, ethnic group, limiting longstanding illness, housing sector and a random effect to allow for clustering at household level.

† Data missing for 133 participants for average daily steps, MVPA and MVPA in bouts

**Supplementary Table 4: Associations between participant characteristics and physical activity variables in participants with at least 4 days of recording of physical activity data**

|  |  | Difference or % difference* in physical activity variable (95% confidence interval), p-value | | | | | | | | |
| --- | --- | --- | --- | --- | --- | --- | --- | --- | --- | --- |
|  | n | Daily steps | | | Daily minutes spent in MVPA | | | Daily minutes spent in MVPA in ≥10 minute bouts | | |
| **Sex** |  |  | | |  | | |  | | |
| Male (Ref) | 402 | - | | | - | | | - | | |
| Female | 529 | -559 | (-947, -172) | 0.005 | -9.7 | (-12.7, -6.7) | <0.0001 | -4.7 | (-6.8, -2.5) | <0.0001 |
| **Age group** |  |  |  |  |  | | |  | | |
| Age 16-24 (Ref) | 180 | - | | | - | | | - | | |
| Age 25-34 | 412 | 410 | (-116, 936) | 0.13 | 3.0 | (-1.2, 7.2) | 0.16 | -0.3 | (-3.3, 2.8) | 0.87 |
| Age 35-49 | 276 | 520 | (-45, 1086) | 0.07 | 2.5 | (-2.0, 6.9) | 0.27 | -2.5 | (-5.7, 0.7) | 0.12 |
| Age 50+ | 63 | -25 | (-889, 838) | 0.95 | -7.9 | (-14.8, -1.1) | 0.02 | -3.2 | (-8.2, 1.8) | 0.20 |
| **Ethnic group** |  |  |  |  |  | | |  | | |
| White (Ref) | 482 | - | | | - | | | - | | |
| Black | 214 | -1213 | (-1789, -638) | <0.0001 | -7.1 | (-11.7, -2.5) | 0.002 | -6.5 | (-9.9, -3.1) | <0.001 |
| Asian | 142 | -1128 | (-1719, -538) | <0.001 | -10.3 | (-15.0, -5.6) | <0.0001 | -7.8 | (-11.3, -4.3) | <0.0001 |
| Other/Mixed | 93 | -582 | (-1273, 110) | 0.10 | -4.7 | (-10.2, 0.8) | 0.09 | -4.2 | (-8.2, -0.2) | 0.04 |
| **Limiting illness** |  |  |  |  |  | | |  | | |
| No (Ref) | 834 | - | | | - | | | - | | |
| Yes | 97 | -976 | (-1612, -341) | 0.003 | -4.6 | (-9.6, 0.4) | 0.07 | -2.0 | (-5.6, 1.6) | 0.28 |
| **Housing sector** |  |  |  |  |  | | |  | | |
| Social | 332 | -978 | (-1515, -440) | <0.001 | -6.9 | (-11.2, -2.6) | 0.002 | -7.2 | (-10.4, -4.0) | <0.0001 |
| Intermediate (Ref) | 410 | - | | | - | | | - | | |
| Market-rent | 189 | -359 | (-889, 171) | 0.185 | 0.2 | (-4.0, 4.3) | 0.91 | 0.9 | (-2.2, 4.1) | 0.56 |

All differences are mutually adjusted for sex, age group, ethnic group, limiting longstanding illness, housing sector and a random effect to allow for clustering at household level.

MVPA and MVPA in ≥10 minute bouts are an average daily estimate, obtained from averaging a participant’s weekly total.

**Supplementary Table 5: Physical activity differences between weekday (Monday-Friday) and weekend (Saturday, Sunday) activity by housing sector.**

| Physical activity variable (N = 1107) | Housing sector group | Mean (95% CI) weekday (Mon-Fri) activity | | Difference in PA outcome compared to weekdays  (95% confidence interval), p-value | | | | | |
| --- | --- | --- | --- | --- | --- | --- | --- | --- | --- |
|  |  |  |  | Saturday - weekday | | | Sunday - weekday | | |
| Daily steps | Social | 8733 | (8364, 9103) | -1643 | (-2078, -1207) | <0.0001 | -2629 | (-3093, -2164) | <0.0001 |
|  | Intermediate | 9497 | (9178, 9817) | 460 | (59, 862) | 0.02 | -1104 | (-1528, -680) | <0.0001 |
|  | Market-rent | 9146 | (8673, 9619) | 1055 | (467, 1642) | <0.001 | -102 | (-734, 531) | 0.75 |
| MVPA (minutes) | Social | 57.2 | (54.3, 60.1) | -11.2 | (-14.7, -7.7) | <0.0001 | -18.4 | (-22.1, -14.7) | <0.0001 |
|  | Intermediate | 63.1 | (60.6, 65.7) | 1.5 | (-1.8, 4.7) | 0.37 | -8.5 | (-11.9, -5.1) | <0.0001 |
|  | Market-rent | 63.5 | (59.8, 67.3) | 6.6 | (1.9, 11.3) | 0.01 | -0.1 | (-5.2, 5.0) | 0.97 |
| MVPA in ≥10 minute bouts (minutes) | Social | 16.3 | (14.0, 18.5) | -4.1 | (-6.9, -1.3) | 0.004 | -6.8 | (-9.8, -3.9) | <0.0001 |
|  | Intermediate | 22.6 | (20.7, 24.6) | 2.5 | (-0.06, 5.1) | 0.06 | -0.7 | (-3.4, 2.0) | 0.62 |
|  | Market-rent | 24.2 | (21.3, 27.1) | 6.1 | (2.4, 9.9) | 0.001 | 2.8 | (-1.2, 6.9) | 0.17 |

Means and differences (95% confidence intervals) are adjusted for sex, age group, ethnic group, limiting longstanding illness, month of recording, day of the week, housing sector, an interaction between housing sector and day of week, and random effects to allow for multiple days of measurement and clustering of participants within household.

MVPA and MVPA in ≥10 minute bouts are an average daily estimate, obtained from averaging a participant’s weekly total.
