# Supplementary material for: Housing, neighbourhood and sociodemographic associations with adult levels of physical activity and adiposity: baseline findings from the ENABLE London study
Source: BMJ Open. 2018 Aug 17;8(8):e021257. doi: 10.1136/bmjopen-2017-021257 (PMC6104748; doi:10.1136/bmjopen-2017-021257)
Supplement: Author's manuscript [file bmjopen-2017-021257.draft_revisions.pdf]

BMJ Open

BMJ Open is committed to open peer review. As part of this commitment we make the peer review history of every article we publish publicly available.

When an article is published we post the peer reviewers' comments and the authors' responses online. We also post the versions of the paper that were used during peer review. These are the versions that the peer review comments apply to.

The versions of the paper that follow are the versions that were submitted during the peer review process. They are not the versions of record or the final published versions. They should not be cited or distributed as the published version of this manuscript.

BMJ Open is an open access journal and the full, final, typeset and author-corrected version of record of the manuscript is available on our site with no access controls, subscription charges or pay-per-view fees (http://bmjopen.bmj.com).

If you have any questions on BMJ Open's open peer review process please email info.bmjopen@bmj.com

BMJ Open

# Housing, neighbourhood and sociodemographic associations with adult levels of physical activity and adiposity: baseline findings from the ENABLE London Study



| | |
|---|---|
| Journal: | *BMJ Open* |
| Manuscript ID | bmjopen-2017-021257 |
| Article Type: | Research |
| Date Submitted by the Author: | 19-Dec-2017 |





| | |
|---|---|
| Complete List of Authors: | Nightingale, C; St George's University London, Division of Population Health Sciences and Education<br>Rudnicka, Alicja; SGUL University of London<br>Ram, Bina; St George's, University of London, Population Health Research Institute<br>Shankar, Aparna; St. Geoege's, University of London, Population Health Research Institute<br>Procter, Duncan; University of Bristol, Centre for Exercise, Nutrition and Health Sciences<br>Cooper, Ashley; University of Bristol,<br>Page, Angie; University of Bristol, Centre for Exercise, Nutrition and Health Sciences<br>Ellaway, Anne; University of Glasgow, MRC/SCO Social and Public Health Sciences Unit<br>Giles-Corti, Billie; RMIT University, NHMRC Centre of Research Excellence in Healthy Liveable Communities<br>Clary, Christelle; London School of Hygiene and Tropical Medicine, Social And Environmental Health Research<br>Lewis, Daniel; London School of Hygiene and Tropical Medicine,<br>Cummins, Steven; London School of Hygiene and Tropical Medicine, Department of Social and Environmental Health Research<br>Whincup, Peter; St Georges, University of London<br>Cook, Derek; St George's University, Division of Population Health Sciences<br>Owen, C; St George's, University London, Population Health Research Institute |





| | |
|---|---|
| Keywords: | Physical activity, Adiposity, Housing, Perceived neighbourhood environment, ENABLE-London |
| | |



SCHOLARONE™ Manuscripts

# Housing, neighbourhood and sociodemographic associations with adult levels of physical activity and adiposity: baseline findings from the ENABLE London Study

CM Nightingale[1], AR Rudnicka[1], B Ram[1], A Shankar[1], D Procter[2,3], AR Cooper[2, 3], AS Page[2, 3], A Ellaway[4], B Giles-Corti[5], C Clary[6], D Lewis[6], S Cummins[6], PH Whincup[1], DG Cook[1], CG Owen[1]

[1] Population Health Research Institute, St George's, University of London, London, UK.

[2] Centre for Exercise, Nutrition and Health Sciences, University of Bristol, Bristol, UK.

[3] National Institute for Health Research Bristol Biomedical Research Centre (Nutrition Theme)

[4] MRC/SCO Social and Public Health Sciences Unit, University of Glasgow, Glasgow, UK.

[5] NHMRC Centre of Research Excellence in Healthy Liveable Communities, RMIT University, Melbourne, Australia.

[6] London School of Hygiene and Tropical Medicine, London, UK.

Corresponding Author:-

Dr Claire M Nightingale

Population Health Research Institute, St. George's, University of London, Cranmer Terrace, London SW17 0RE. Email: cnightin@sgul.ac.uk. Phone: +44 (0) 208 725 2342

**Total word count:** 3996 excluding abstract (300 words)

**Tables and figures:** 3 tables, 1 figure, 5 supplementary tables

**References:** 48



For peer review only - http://bmjopen.bmj.com/site/about/guidelines.xhtml

## *Abstract*

**Objectives:** The neighbourhood environment is increasingly shown to be an important correlate of health. We assessed associations between housing tenure, neighbourhood perceptions, sociodemographic factors, and levels of physical activity (PA) and adiposity among adults seeking housing in East Village (formerly London 2012 Olympic/Paralympic Games Athletes' Village).

**Setting:** Cross-sectional analysis of adults seeking social, intermediate and market-rent housing in East Village.

**Participants:** 1278 participants took part in the study (58% female). Complete data on adiposity (body mass index [BMI] and fat mass %) were available for 1240 participants (97%); of these a sub-set of 1107 participants (89%) met the inclusion criteria for analyses of accelerometer-based measurements of PA. We examined associations between housing sector sought, neighbourhood perceptions (covariates) and PA and adiposity (dependent variables) adjusted for household clustering, sex, age group, ethnic group, and limiting longstanding illness.

**Results:** Participants seeking social housing had the fewest steps (8304, 95%CI 7959,8648) and highest BMI (26.0kg/$m^2$ 95%CI 25.5,26.5kg/$m^2$) compared with those seeking intermediate (steps 9417, 95%CI 9106,9731; BMI 24.8kg/$m^2$ 95%CI 24.4,25.2kg/$m^2$) or market-rent housing (steps 9313, 95%CI 8858,9768; BMI 24.6kg/$m^2$ 95%CI 24.0,25.2kg/$m^2$). Those seeking social housing had lower levels of PA (by 19-42%) at weekends vs weekdays, compared with other housing groups. Positive perceptions of neighbourhood quality were associated with higher steps and lower BMI, with differences between social and



For peer review only - http://bmjopen.bmj.com/site/about/guidelines.xhtml

intermediate groups reduced by ~10% following adjustment, equivalent to a reduction of 111 for steps and 0.5kg/m$^2$ for BMI.

**Conclusions:** The social housing group undertook less PA than other housing sectors, with weekend PA offering the greatest scope for increasing PA, and tackling adiposity in this group. Perceptions of neighbourhood quality were associated with PA and adiposity and reduced differences in steps and BMI between housing sectors. Moving to East Village may provide scope to encourage PA and reduce adiposity amongst the most disadvantaged.

**Strengths and limitations of this study**

- Large sample with representation of three different aspirational housing groups, providing a wide range of socioeconomic backgrounds
- Objective measurements of physical activity and adiposity outcomes using accelerometry and bioelectrical impedance respectively
- Lower number of participants studied seeking market-rent housing compared with those seeking intermediate or social housing

**Keywords**

Physical activity; Adiposity; Housing; Perceived neighbourhood environment; ENABLE-London


For peer review only - http://bmjopen.bmj.com/site/about/guidelines.xhtml

## *Introduction*

Physical inactivity and adiposity are associated with an increased risk of type 2 diabetes and cardiovascular disease (1-4) and constitute a serious public health problem in the UK and globally (5). Evidence suggests that levels of physical activity (PA) are lower among those who are socioeconomically disadvantaged (6), who experience greater economic, access and health related barriers to being physically active (7). Socioeconomic status is also associated with differences in types of PA, in particular higher socioeconomic status is associated with more vigorous leisure time PA (8). Previous research has found variation in PA by day of the week with studies showing lower levels of activity on Sundays compared with weekdays in young adults (9), parents and their children (10).

There is emerging evidence suggesting that housing tenure is an important determinant of health. In particular, UK-based studies have shown that housing tenure (owner vs. private renter vs. public sector renter) is associated with illness and mortality (15;16). Amongst particular groups including those who are economically inactive or unemployed, housing tenure might provide a better indication of socioeconomic status compared with measures based on occupation or income (11). Indeed, in several studies housing tenure remained associated with health outcomes following adjustment for conventional measures of socioeconomic status such as income or education (12;13). A more nuanced approach is therefore required with respect to measures of socioeconomic status, and they should not be simply regarded as interchangeable (14;15). Despite this, there has been limited research examining the direct effect of housing tenure on PA, and existing evidence is equivocal. Harrison and colleagues found no association between housing tenure and meeting recommended levels of PA among community dwelling healthy adults in the North-East of


For peer review only - http://bmjopen.bmj.com/site/about/guidelines.xhtml

England (16). Similarly housing tenure was not associated with self-reported energetic PA among older Australians (17). Ogilvie and colleagues found overall levels of PA to be higher among individuals living in social rented accommodation compared with owner-occupiers (18). The authors suggest that may capture occupational PA levels which are likely to be higher among social renters (18). In contrast, living in private rental accommodation was associated with a greater likelihood of taking up exercise over a 9-year period among men aged 18-49 at baseline, compared with those in local authority accommodation (19).

Housing tenure may affect health and health behaviours in part through characteristics of the home or neighbourhood itself (20;21) or psychological factors such as self-efficacy or self-esteem (22). Social housing estates which are common in the UK may be associated with specific cultures and norms, which in turn shape residents' behaviours (13). Subjective characteristics of the neighbourhood environment including higher perceived access to recreational facilities and shops in local proximity have been shown to be associated with higher levels of PA (23;24). Residents who perceive their neighbourhood more positively, have been shown to have better mental health and are less likely to relocate (25). Conversely, real and perceived crime, has the potential to constrain resident's PA (26). However, a recent systematic review suggested a lack of association between PA and perceptions of safety from crime; highlighting the need for high quality evidence, including prospective studies and natural experiments (27), to examine this issue further. In particular, high quality evidence is needed to understand the potentially multifactorial influence of residential location on health and health behaviours; effects which are likely to extend beyond simple measures of socioeconomic status (27).


For peer review only - http://bmjopen.bmj.com/site/about/guidelines.xhtml

The Examining Neighbourhood Activities and Built Living Environments in London (ENABLE London) study is a longitudinal study evaluating how active urban design influences the health and wellbeing of people moving in to the former Athletes' Village of the London 2012 Olympic and Paralympic Games now known as 'East Village' (28). East Village is a new high-density neighbourhood development built on active design principles containing a mix of social housing, intermediate (including affordable rent, shared ownership and shared equity) housing, and market-rent housing. This paper draws on baseline data (prior to any potential move to East Village) to first, examine predictors of PA and adiposity (measured objectively using accelerometry and bioelectrical impedance), including the housing sector to which they are applying and perceptions of their neighbourhood. Second, to examine whether PA patterns across the week vary by housing sector and third, to examine whether adjustment for perceptions of the neighbourhood environment reduce housing sector differences in PA and adiposity.

## *Methods*

Study participants were recruited from those seeking new accommodation in East Village and were classified by the type of housing tenure sought; social, intermediate or market-rent. Current housing status was strongly linked to aspirational housing status, where those seeking social accommodation were currently in social housing or on social housing waiting lists, and those seeking intermediate and market-rent accommodation were largely in privately rented housing. Recruitment of participants in the different housing sectors was carried out between January 2013 and December 2015 in three phases determined by the order of availability of housing in East Village (social, intermediate, and market-rent respectively). Social housing is provided by the local authority or housing association at


For peer review only - http://bmjopen.bmj.com/site/about/guidelines.xhtml

subsidised rates. Baseline assessments of participants were carried out in their place of residence before any potential move to East Village. Full details of the recruitment process can be found elsewhere (28).

**Independent variables**

A team of trained fieldworkers administered self-complete questionnaires on a laptop during home visits. Data on age, sex, self-defined ethnicity, work status, occupation and whether the participant had a limiting longstanding illness or disability (lasting or expected to last at least 12 months) were collected. Participants self-defined as 'White', 'Asian', 'Black', 'Mixed', or 'Other'; the latter two categories were combined for analyses. Socioeconomic status based on occupation was coded using the National Statistics Social-Economic Coding (NS-SEC) to categorise participants into 'higher managerial or professional occupations', 'intermediate occupations', 'routine or manual' (28). An additional 'economically inactive' category included those seeking employment, unable to work due to disability or illness, retired, looking after home and family, and students. We sought information on educational attainment; participants were categorised into "Degree or equivalent / Higher", "Intermediate qualifications" (including A levels and GSCEs), and "Other / None" (including work-based or foreign qualifications). Participants completed questionnaires assessing neighbourhood perceptions. Five items assessed perceived crime (e.g "There is a lot of crime in my neighbourhood"; Cronbach's $\alpha = 0.87$) and six items assessed neighbourhood quality (e.g. "This area is a place I enjoy living in"; Cronbach's $\alpha$ $=0.78$). Responses on items were summed and scores ranged from -10 to +10 for perceived crime and -12 to +12 for perceived quality, such that positive scores indicate less perceived crime and better neighbourhood quality while negative scores indicate more perceived


For peer review only - http://bmjopen.bmj.com/site/about/guidelines.xhtml

crime and poorer quality. The scales were derived following an exploratory factor analysis of 14 questions regarding neighbourhood (Supplementary Table 1).

**Dependent variables**

Height was measured to the last complete millimetre using a portable stadiometer; weight was measured to the nearest kilogram using a Tanita SC-240 Body Composition Analyzer (Tanita, Tokyo, Japan); body mass index (BMI) was derived as weight(kg)/height(m)$^2$. The Tanita SC-240 Body Composition Analyzer also measured leg-to-leg bioelectrical impedance from which fat free mass and fat mass were estimated. Fat mass percentage was calculated as fat mass(kg)/weight(kg)*100.

Participants wore a hip-mounted ActiGraph GT3X+ accelerometer during waking hours over a consecutive period of 7 days (ActiGraph LLC, Florida, USA). These accelerometers provided daily measures of steps, counts and time spent in moderate and vigorous PA (MVPA) using established cut-offs. Daily time spent in MVPA both overall and in 10 minute bouts in accordance with UK recommendations for PA (30) were assessed. The cut-point for moderate PA was defined as 5724 counts per minute (31). We excluded any days of recording where the amount of registered time accumulated was below 540 minutes (32). Non-wear periods were defined as a minimum length of 60 minutes, allowing for a 2-minute spike tolerance. Participants with at least one day of recording were retained in analyses. We fitted a multilevel linear model for each outcome to allow for repeated measurements of daily PA, by fitting participant as a random effect and adjusting for day of the week, day order of recording and month as fixed effects. Raw level one residuals were obtained from the model and a within person average value of each outcome variable was obtained by



For peer review only - http://bmjopen.bmj.com/site/about/guidelines.xhtml

averaging these raw residuals. The average of these raw residuals for each participant was added to the sample mean for that particular PA variable to derive an unbiased average level of each PA variable for each person.

**Statistical analysis**

All analyses were carried out using STATA/SE software (Stata/SE 14 for Windows; StataCorp LP, College Station, TX, USA). Outcome variables were inspected for normality and BMI was log transformed due to its skewed distribution. Multilevel linear regression models were fitted, mutually adjusted for housing sector and participant characteristics (sex, age group, ethnic group, and limiting longstanding illness) as fixed effects and a random effect to allow for household clustering. Absolute differences or percentage differences for are presented by sex, age group, ethnic group, limiting longstanding illness and housing sector. Sensitivity analyses examined whether associations remained when the sample was restricted to 931 participants (84%) with at least four days of 540 or more minutes per day of recording.

To assess differences in PA by day of the week as opposed to overall levels of PA we took the following approach. Daily PA data were examined using multilevel models with random effects to allow for multiple days of recording within person and household clustering. An interaction between housing sector and day of the week was fitted and models were adjusted for sex, age group, ethnic group, limiting longstanding illness, day order of recording and month of measurement as fixed effects.

Associations between perceptions of neighbourhood crime and quality with adiposity/PA outcomes were assessed. The effect of adjustment for neighbourhood perceptions in


For peer review only - http://bmjopen.bmj.com/site/about/guidelines.xhtml

addition to adjustment for participant characteristics using multilevel models as described above on differences in outcomes between housing sector groups was also examined.

## *Results*

Of 1819 households who consented to initial contact by the study team, 1278 participants from 1006 households (55%) were enrolled in the study and completed a questionnaire. Participation rates for those seeking market-rent and intermediate housing were 58% and 57% respectively and were slightly lower in the social group (52%). Complete data on adiposity were available for 1240 participants (97%); of these a sub-set of 1107 participants (89%) met the inclusion criteria for analyses of objectively measured PA. Participant characteristics (age, sex) and levels of adiposity were similar among those who did and did not provide PA data; however, participants from black and Asian ethnic groups were less likely to provide PA data. Supplementary Table 2 shows participants characteristics at baseline for the 1240 adults with measurements of adiposity at baseline. Those seeking social housing were more likely to be female, of older age, of non-white ethnicity, to have limiting longstanding illness, and be in routine / manual occupations or economically inactive compared to those seeking intermediate or market-rent housing.

Adjusted mean levels of adiposity and PA outcomes by housing sector and participant characteristics are shown in Supplementary Table 3. Table 1 shows housing sector and other participant characteristics associations with adiposity markers (BMI, fat mass %) and objectively measured PA (steps, time spent in MVPA, time spent in MVPA in 10 minute bouts). Participants seeking social housing had markedly higher levels of BMI and fat mass % and markedly lower levels of steps, MVPA and MVPA in 10 minute bouts compared with


For peer review only - http://bmjopen.bmj.com/site/about/guidelines.xhtml

those seeking intermediate housing, though there were no differences between those seeking market-rent and intermediate accommodation.

Fat mass % was higher in females than males though there was no difference in BMI (Table 1). BMI and fat mass % were higher among all older age groups compared with 16-24 year olds. Participants of black ethnicity had higher levels of BMI and fat mass % compared with whites; there were no differences in adiposity between Asian or other/mixed ethnic groups and whites. Those with a limiting longstanding illness had higher levels of both BMI and fat mass %. All PA measures were lower among females. Steps and MVPA were slightly higher in 25-34 year olds and steps were also higher among 35-49 year olds compared with 16-24 year olds; however, there were no age group differences for MVPA in 10 minute bouts. Participants of black and Asian ethnicities had lower levels of steps, MVPA and MVPA in 10 minute bouts compared to whites. Participants who reported having a limiting longstanding illness had lower levels of steps and MVPA, but not MVPA in 10 minute bouts. Educational attainment level was not associated with any of the outcomes once housing sector had been adjusted for and adjustment for educational attainment did not materially alter housing sector differences in adiposity or PA outcomes (data available from authors).

Sensitivity analyses for PA outcomes were carried out in 931 participants who wore an ActiGraph for at least four days with at least 540 minutes of recording per day (Supplementary Table 4). There were no differences between market-rent and intermediate groups (consistent with the main analysis presented in Table 1). Differences between social and intermediate groups were broadly similar with the results presented in Table 1 for the main analysis.


For peer review only - http://bmjopen.bmj.com/site/about/guidelines.xhtml

Differences in PA variables between housing groups were examined by day of the week to explore whether differences between groups were consistent across the week (Figure 1A-D). Levels of PA (steps (panel A), MVPA (panel B) and MVPA in 10 minute bouts (panel C)) were generally consistent across weekdays (Monday – Friday) among all groups. In the intermediate group, steps were higher on Saturdays and lower on Sundays; MVPA and MVPA in 10 minute bouts were lower on Sundays but there was no difference on Saturdays compared to weekday activity. In the market-rent group, steps, MVPA and MVPA in 10 minute bouts were higher on Saturdays and similar to weekdays on Sundays. In the social group, steps, MVPA and MVPA in 10 minute bouts were on average lower on Saturdays and lower still on Sundays. Registered time (panel D) was lowest on average in the social group during weekdays, decreasing on Saturdays and Sundays. The intermediate and market-rent groups had higher levels of registered time during weekdays compared with the social group which decreased on average on Saturdays and Sundays (despite recording more steps and minutes in MVPA suggesting a higher intensity of activity). Mean levels of steps, MVPA, and MVPA in 10 minute bouts on weekdays and differences on Saturday and Sunday compared to weekdays are shown by housing sector in Supplementary Table 5. The marked differences in activity between weekdays and weekend days in the social group are not explained by differences in registered time (data available from authors).

Associations between perceived neighbourhood quality and crime scales and adiposity and PA outcomes are shown in Table 2, adjusted for the participant characteristics shown in Table 1. All associations between perceived neighbourhood quality and crime and outcome variables were approximately linear and were therefore fitted as continuous variables in the model. In addition, associations between perceived neighbourhood quality and crime and



For peer review only - http://bmjopen.bmj.com/site/about/guidelines.xhtml

outcome variables were similar across the three housing groups (all $p>0.05$). The differences between the highest and lowest quintile of each scale are presented for perceptions of neighbourhood quality (median: 4, IQR: 0, 7) and neighbourhood crime (median: 2, IQR: -1, 5). Participants with the most positive perceptions of neighbourhood quality (highest quintile) had lower BMI, higher steps and recorded longer durations of MVPA compared with those who had the most negative perceptions of neighbourhood quality (lowest quintile). There were no significant associations between perceptions of neighbourhood crime and adiposity or PA.

The effect of adjustment for perceived neighbourhood quality on differences in adiposity and PA between housing sector groups is presented in Table 3. Adjustment for perceptions of neighbourhood quality reduced differences in BMI, fat mass %, steps, MVPA and MVPA in 10 minute bouts between the social and intermediate groups by 10%, 6%, 10%, 10% and 7% respectively. Differences between market-rent and intermediate groups in adiposity and PA variables were not statistically significant before or after adjustment. A larger proportion of the social-intermediate group differences in steps, MVPA and MVPA in 10 minute bouts on weekends was explained by adjustment for perceptions of neighbourhood quality (11%, 16% and 17% respectively) compared to the differences in steps, MVPA and MVPA in 10 minute bouts on weekdays which were reduced by 10%, 8% and 3% respectively.

## *Discussion*

The results of this study showed that participants seeking social housing in East Village had lower levels of PA and higher levels of adiposity compared with those seeking intermediate and market-rent housing, even when adjusted for demographic factors. In the social



For peer review only - http://bmjopen.bmj.com/site/about/guidelines.xhtml

housing group, levels of PA were particularly low on weekends compared with weekdays possibly reflecting higher occupational PA and lower leisure time PA; weekday-weekend differences in PA were less marked among those seeking intermediate and market-rent housing. However, the lower registered time at weekends but higher MVPA and steps suggests more intense activity at weekends in the intermediate and market-rent housing groups. These findings may inform targeted interventions to increase PA and reduce adiposity in different socioeconomic groups.

Positive associations between perceived neighbourhood quality and PA and adiposity were also shown. Adjustment for differences in perceived neighbourhood quality reduced differences in PA and adiposity by approximately 10% between social and intermediate housing groups; equivalent to a reduction of 111 for daily steps, 0.5 minutes for MVPA and 0.5kg/m$^2$ for BMI. However, a larger proportion of the difference in PA was apparent at weekends; equivalent to a reduction of 222 for daily steps and 2.2 minutes for MVPA.

**Relation to previous studies**

Studies have shown that lower socioeconomic status is associated with lower levels of PA (33;34), and that those from more socially deprived backgrounds have the most barriers to being physically active (7). Previous research examining the role of housing tenure is limited. Findings from this study showed marked differences in PA and adiposity between those seeking social, intermediate and market-rent housing. In particular, lower PA and higher adiposity in participants seeking social housing, a group which comprises a high proportion of people from more socioeconomically disadvantaged backgrounds (28). The higher levels of adiposity in those seeking social housing compared with those seeking intermediate or


For peer review only - http://bmjopen.bmj.com/site/about/guidelines.xhtml

market-rent housing is consistent with systematic reviews which have found an association between lower socioeconomic status and higher levels of adiposity, particularly in higher income countries and among women (35). While socioeconomic status is a strong determinant of housing status, to our knowledge this is the first study to explicitly examine housing sector differences in objective PA and adiposity levels. However, it is important to consider more broadly what these aspirational housing sector differences might represent. Related studies have shown that those in social housing are less likely to use active travel compared with owner occupiers (18), and that those in social housing and home owners with a mortgage are more likely to be obese and have higher levels of illness and disability compared to outright home owners, even after adjustment for other socioeconomic status markers (36). These latter findings suggest that the effect of home ownership may be more complex and cannot be simply explained by socioeconomic status. Neighbourhood quality may offer a potential partial explanation for these findings (37). In the present study perceptions of better neighbourhood quality were associated with PA whereas perceptions of crime were not. In contrast, a large UK-based study found that perceptions of feeling safe in the neighbourhood had the largest effect on levels of PA compared with perceptions of leisure facilities, sense of belonging or access to public transport or amenities (38). Another study in the US found that low perceived safety from crime was associated with lower levels of MVPA (39). However, a recent review concluded that higher quality evidence is needed, including prospective studies and natural experiments in areas of wide crime variability, in order to further understand the effect of crime on physical and mental health (27). Moreover, effects of perceived and objective measures of neighbourhood quality may have differing and potentially independent effects on health behaviours including PA (40).


For peer review only - http://bmjopen.bmj.com/site/about/guidelines.xhtml

Our findings showed that PA levels were particularly low on the weekend among those seeking social housing, which is consistent with findings from a systematic review which found that leisure-time PA (which may be more likely to occur on weekends) was lower amongst those from lower socioeconomic groups (8). This suggests that low-cost strategies to increase weekend PA may be particularly beneficial to more disadvantaged households. A free community-based program in Bogata Colombia, temporarily closed streets on Sundays to encourage PA amongst more disadvantaged local residents (41). A similar program has been trialled in the United States (42), however the effectiveness, longevity and generalisability of these programs to other socioeconomically deprived areas is yet to be established.

**Strengths and limitations**

Strengths of this study include the representation of three different aspirational housing groups which provides a wide range of socioeconomic backgrounds. Of those seeking social housing, two-thirds (67%) were currently living in social housing accommodation provided by the local authority or housing association; the remainder were largely currently living in privately rented accommodation with many on social housing waiting lists. Of those seeking intermediate or market-rent accommodation, almost two-thirds were living in privately rented accommodation (both 64%); the remainder were largely living with relatives or friends. The study sample is large with good representation from a 'hard to reach' group of social housing participants. Participation rates were high given the target group, with between 50-60% of those who initially agreed to be contacted taking part in the study. The ActiGraph GT3X+ accelerometer provided validated objective measures of PA (43) and the use of bioelectrical impedance to provide more direct measurements of adiposity including


For peer review only - http://bmjopen.bmj.com/site/about/guidelines.xhtml
For peer review only

fat mass %, which may provide a more valid marker of adiposity than BMI, particularly in a multi-ethnic population (44;45). Reassuringly the patterns of PA by sex, ethnic group and health status were consistent with those published previously (46-48). A limitation of the study is the lower number of participants in the market-rent sector compared with the other groups. This was due to restrictions imposed on the study team on the extent and duration of access to potential applicants seeking market-rent accommodation. While the study is longitudinal, these analyses are cross-sectional limiting the degree to which causal inferences can be made. Moreover, there is the possibility of selection amongst study participants, where those who are more active seek to move to East Village, may be more likely to participate in the study and may perceive their environment differently, which may limit the generalisability of the findings to neighbourhoods outside of East London.

**Conclusions and future work**

The findings presented in this paper suggest that perceived neighbourhood quality is associated with PA and that there are substantial differences in PA and adiposity levels between the three housing groups studied. In particular the very low levels of PA in the social housing group during the weekend could provide a target for intervention to increase levels of PA. Perceptions of neighbourhood quality reduced differences in PA and adiposity between housing sector groups, and the possibility of measuring more objective markers of neighbourhood quality within this study has the potential to explain more (40). The future follow-up of the ENABLE London cohort will allow us to examine whether moving to ‘East Village’, a neighbourhood designed for healthy active living, will have a positive impact on PA and/or adiposity levels. A major aim of the study is to identify features of the local built environment that increase levels PA which could potentially help to reduce socioeconomic


For peer review only - http://bmjopen.bmj.com/site/about/guidelines.xhtml

inequalities in health. It will be of particular interest to determine whether an increase in PA is more apparent in the social housing group whose neighbourhood characteristics should improve. Furthermore, we will be in a position to examine whether any potential effects of the built environment on PA are modified by housing sector type.

For peer review only



For peer review only - http://bmjopen.bmj.com/site/about/guidelines.xhtml

Ethical approval

Full ethical approval was obtained from the relevant Multi-Centre Research Ethics Committee (REC Reference 12/LO/1031). All participants provided written informed consent.

Availability of data and material

Further details of the ENABLE London study are available from the study website (http://www.enable.sgul.ac.uk/). The ongoing collection and management of data has been made possible through grant funding from the Medical Research Council and the National Institute of Health Research. We welcome proposals for collaborative projects. For general data sharing inquiries, contact Professor Owen (cowen@sgul.ac.uk).

Competing interests

We declare that we have no competing interests.

Funding

This research is being supported by project grants from the Medical Research Council National Prevention Research Initiative (MR/J000345/1) and National Institute for Health Research (12/211/69). Diabetes and obesity prevention research at St George's, University of London is supported by the National Institute for Health Research (NIHR) Collaboration for Leadership in Applied Health Research and Care (CLAHRC) South London. AE is funded by the UK Medical Research Council as part of the Neighbourhoods and Communities Programme (MC_UU_12017-10). BG-C is supported by an NHMRC Principal Research Fellowship (#1107672). ARC is supported by National Institute for Health Research (NIHR)



For peer review only - http://bmjopen.bmj.com/site/about/guidelines.xhtml

Bristol Nutrition Biomedical Research Unit based at University Hospitals Bristol NHS Foundation Trust and the University of Bristol.

Authorship statement

CGO, ARR, AE, ARC, DL, SC, BG-C, DGC and PHW designed the study and raised funding. BR, ARR, CC, DP and CGO collected data for the study; BR, ARR and CGO enrolled participants. CMN, BR, ARR, CC, DP and CGO undertook data management. CMN analysed the data and wrote the first draft of the report. ARR, BR, AS, DP, ARC, ASP, AE, BG-C, CC, DL, SC, PHW, DGC and CGO critically appraised the manuscript and approved the final draft. CGO is responsible for data integrity.

Acknowledgements

The authors thank the East Thames Group, Triathlon Homes and Get Living London who have assisted in recruiting participants into the ENABLE London study. The ENABLE London study is advised by a Steering Committee chaired by Professor Hazel Inskip (University of Southampton), with Dr David Ogilvie (University of Cambridge) and Professor Andy Jones (University of East Anglia) as academic advisors and Mrs Kate Worley (formerly East Thames Group Assistant Director for Strategic Housing) as the lay/stakeholder member. The authors are grateful to the members of the ENABLE London study team (in particular Aine Hogan, Katrin Peuker, Cathy McKay) and to participating households, without whom this study would not be possible.


For peer review only - http://bmjopen.bmj.com/site/about/guidelines.xhtml

## References


(1) Ford ES, Williamson DF, Liu S. Weight change and diabetes incidence: findings from a national cohort of US adults. Am J Epidemiol 1997;146:214-22.

(2) Nocon M, Hiemann T, Muller-Riemenschneider F, Thalau F, Roll S, Willich SN. Association of physical activity with all-cause and cardiovascular mortality: a systematic review and meta-analysis. Eur J Cardiovasc Prev Rehabil 2008;15:239-46.

(3) Shaper AG, Wannamethee SG, Walker M. Body weight: implications for the prevention of coronary heart disease, stroke, and diabetes mellitus in a cohort study of middle aged men. BMJ 1997;314:1311-7.

(4) Whitlock G, Lewington S, Sherliker P, Clarke R, Emberson J, Halsey J, et al. Body-mass index and cause-specific mortality in 900 000 adults: collaborative analyses of 57 prospective studies. Lancet 2009;373:1083-96.

(5) Lim SS, Vos T, Flaxman AD, Danaei G, Shibuya K, Adair-Rohani H, et al. A comparative risk assessment of burden of disease and injury attributable to 67 risk factors and risk factor clusters in 21 regions, 1990-2010: a systematic analysis for the Global Burden of Disease Study 2010. Lancet 2012;380:2224-60.

(6) Health Survey for England. Health Survey for England 2012: Health, social care and lifestyles. The Information Centre; 2013 Dec.

(7) Chinn DJ, White M, Harland J, Drinkwater C, Raybould S. Barriers to physical activity and socioeconomic position: implications for health promotion. J Epidemiol Community Health 1999;53:191-2.

(8) Beenackers MA, Kamphuis CB, Giskes K, Brug J, Kunst AE, Burdorf A, et al. Socioeconomic inequalities in occupational, leisure-time, and transport related physical activity among European adults: a systematic review. Int J Behav Nutr Phys Act 2012;9:116.

(9) McVeigh JA, Winkler EA, Howie EK, Tremblay MS, Smith A, Abbott RA, et al. Objectively measured patterns of sedentary time and physical activity in young adults of the Raine study cohort. Int J Behav Nutr Phys Act 2016;13:41.

(10) Sigmundova D, Sigmund E, Vokacova J, Kopkova J. Parent-child associations in pedometer-determined physical activity and sedentary behaviour on weekdays and weekends in random samples of families in the Czech Republic. Int J Environ Res Public Health 2014;11:7163-81.

(11) Smith GD, Egger M. Socioeconomic differences in mortality in Britain and the United States. Am J Public Health 1992;82:1079-81.

(12) Dalstra JA, Kunst AE, Mackenbach JP. A comparative appraisal of the relationship of education, income and housing tenure with less than good health among the elderly in Europe. Soc Sci Med 2006;62:2046-60.

(13) Ellaway A, Macdonald L, Kearns A. Are housing tenure and car access still associated with health? A repeat cross-sectional study of UK adults over a 13-year period. BMJ Open 2016;6:e012268.




For peer review only - http://bmjopen.bmj.com/site/about/guidelines.xhtml

For peer review only

(14) Macintyre S, Hiscock R, Kearns A, Ellaway A. Housing tenure and car access: further exploration of the nature of their relations with health in a UK setting. J Epidemiol Community Health 2001;55:330-1.

(15) Macintyre S, McKay L, Der G, Hiscock R. Socio-economic position and health: what you observe depends on how you measure it. J Public Health Med 2003;25:288-94.

(16) Harrison RA, McElduff P, Edwards R. Planning to win: Health and lifestyles associated with physical activity amongst 15,423 adults. Public Health 2006;120:206-12.

(17) Kendig H, Browning C, Teshuva K. Health actions and social class among older Australians. Aust N Z J Public Health 1998;22:808-13.

(18) Ogilvie D, Mitchell R, Mutrie N, Petticrew M, Platt S. Personal and environmental correlates of active travel and physical activity in a deprived urban population. Int J Behav Nutr Phys Act 2008;5:43.

(19) Boniface DR, Cottee MJ, Neal D, Skinner A. Social and demographic factors predictive of change over seven years in CHD-related behaviours in men aged 18-49 years. Public Health 2001;115:246-52.

(20) Macintyre S, Ellaway A, Hiscock R, Kearns A, Der G, McKay L. What features of the home and the area might help to explain observed relationships between housing tenure and health? Evidence from the west of Scotland. Health Place 2003;9:207-18.

(21) Ellaway A, Macintyre S. Does housing tenure predict health in the UK because it exposes people to different levels of housing related hazards in the home or its surroundings? Health Place 1998;4:141-50.

(22) Macintyre S, Ellaway A, Der G, Ford G, Hunt K. Do housing tenure and car access predict health because they are simply markers of income or self esteem? A Scottish study. J Epidemiol Community Health 1998;52:657-64.

(23) Hoehner CM, Brennan Ramirez LK, Elliott MB, Handy SL, Brownson RC. Perceived and objective environmental measures and physical activity among urban adults. Am J Prev Med 2005;28:105-16.

(24) Sallis JF, Bowles HR, Bauman A, Ainsworth BE, Bull FC, Craig CL, et al. Neighborhood environments and physical activity among adults in 11 countries. Am J Prev Med 2009;36:484-90.

(25) Giles-Corti B, Ryan K, Foster S. Increasing density in Australia: Maximising the benefits and minimising the harm. National Heart Foundation of Australia; 2012.

(26) Foster S, Giles-Corti B. The built environment, neighborhood crime and constrained physical activity: an exploration of inconsistent findings. Prev Med 2008;47:241-51.

(27) da Silva IC, Payne VL, Hino AA, Varela AR, Reis RS, Ekelund U, et al. Physical Activity and Safety From Crime Among Adults: A Systematic Review. J Phys Act Health 2016;13:663-70.

(28) Ram B, Nightingale CM, Hudda MT, Kapetanakis VV, Ellaway A, Cooper AR, et al. Cohort profile: Examining Neighbourhood Activities in Built Living Environments in London: the ENABLE London-Olympic Park cohort. BMJ Open 2016;6:e012643.



For peer review only - http://bmjopen.bmj.com/site/about/guidelines.xhtml

(29) Office for National Statistics. The National Statistics Socio-economic Classification User Manual. 2005.

(30) Department of Health. Start active, stay active: a report on physical activity from the four home countries Chief Medical Officers. Department of Health; 2011.

(31) Freedson PS, Melanson E, Sirard J. Calibration of the Computer Science and Applications, Inc. accelerometer. Med Sci Sports Exerc 1998;30:777-81.

(32) Harris T, Kerry SM, Limb ES, Victor CR, Iliffe S, Ussher M, et al. Effect of a Primary Care Walking Intervention with and without Nurse Support on Physical Activity Levels in 45- to 75-Year-Olds: The Pedometer And Consultation Evaluation (PACE-UP) Cluster Randomised Clinical Trial. PLoS Med 2017;14:e1002210.

(33) Allen MS, Vella SA. Longitudinal determinants of walking, moderate, and vigorous physical activity in Australian adults. Prev Med 2015;78:101-4.

(34) Juneau CE, Sullivan A, Dodgeon B, Cote S, Ploubidis GB, Potvin L. Social class across the life course and physical activity at age 34 years in the 1970 British birth cohort. Ann Epidemiol 2014;24:641-7, 647.

(35) McLaren L. Socioeconomic status and obesity. Epidemiol Rev 2007;29:29-48.

(36) Tranter B, Donoghue J. Housing tenure, body mass index and health in Australia. International Journal of Housing Policy 2016;1:1-20.

(37) Turrell G, Haynes M, Wilson LA, Giles-Corti B. Can the built environment reduce health inequalities? A study of neighbourhood socioeconomic disadvantage and walking for transport. Health Place 2013;19:89-98.

(38) Harrison RA, Gemmell I, Heller RF. The population effect of crime and neighbourhood on physical activity: an analysis of 15,461 adults. J Epidemiol Community Health 2007;61:34-9.

(39) Brown BB, Werner CM, Smith KR, Tribby CP, Miller HJ. Physical activity mediates the relationship between perceived crime safety and obesity. Prev Med 2014;66:140-4.

(40) McGinn AP, Evenson KR, Herring AH, Huston SL, Rodriguez DA. Exploring associations between physical activity and perceived and objective measures of the built environment. J Urban Health 2007;84:162-84.

(41) Sarmiento O, Torres A, Jacoby E, Pratt M, Schmid TL, Stierling G. The Ciclovia-Recreativa: A mass-recreational program with public health potential. J Phys Act Health 2010;7 Suppl 2:S163-S180.

(42) Hipp JA, Eyler AA, Zieff SG, Samuelson MA. Taking physical activity to the streets: the popularity of Ciclovia and Open Streets initiatives in the United States. Am J Health Promot 2014;28:S114-S115.

(43) Santos-Lozano A, Santin-Medeiros F, Cardon G, Torres-Luque G, Bailon R, Bergmeir C, et al. Actigraph GT3X: validation and determination of physical activity intensity cut points. Int J Sports Med 2013;34:975-82.


For peer review only - http://bmjopen.bmj.com/site/about/guidelines.xhtml

(44) Deurenberg P, Yap M, van Staveren WA. Body mass index and percent body fat: a meta analysis among different ethnic groups. Int J Obes Relat Metab Disord 1998;22:1164-71.

(45) Gallagher D, Visser M, Sepulveda D, Pierson RN, Harris T, Heymsfield SB. How useful is body mass index for comparison of body fatness across age, sex, and ethnic groups? Am J Epidemiol 1996;143:228-39.

(46) Hayes L, White M, Unwin N, Bhopal R, Fischbacher C, Harland J, et al. Patterns of physical activity and relationship with risk markers for cardiovascular disease and diabetes in Indian, Pakistani, Bangladeshi and European adults in a UK population. J Public Health Med 2002;24:170-8.

(47) Health and Social Care Information Centre. Health Survey for England 2004: The health of ethnic minority groups. The Information Centre; 2006.

(48) Health and Social Care Information Centre. Health Survey for England - 2008: Physical activity and fitness. NHS Digital; 2009.

For peer review only



For peer review only - http://bmjopen.bmj.com/site/about/guidelines.xhtml

Table 1: Associations between participant characteristics and adiposity and physical activity

| | | Difference/% difference* in adiposity/physical activity | | | | | | | | | | | | | | |
|---|---|---|---|---|---|---|---|---|---|---|---|---|---|---|---|---|
| | n | BMI (kg/m$^2$)* | | | Fat mass % | | | Steps† | | | Minutes spent in MVPA† | | | Minutes spent in MVPA in 10 minute bouts† | | |
| **Sex** | | | | | | | | | | | | | | | | |
| Male (Ref) | 522 | | | - | | | - | | | - | | | - | | | - |
| Female | 718 | -1.16 | (-3.17, 0.89) | 0.26 | 11.11 | (10.25, 11.98) | <0.0001 | -570 | (-946, -194) | 0.003 | -9.29 | (-12.21, -6.36) | <0.0001 | -4.06 | (-6.12, -2.01) | <0.001 |
| **Age group** | | | | | | | | | | | | | | | | |
| Age 16-24 (Ref) | 269 | | | - | | | - | | | - | | | - | | | - |
| Age 25-34 | 531 | 6.26 | (3.51, 9.08) | <0.0001 | 3.20 | (2.10, 4.31) | <0.0001 | 502 | (11, 992) | 0.04 | 4.03 | (0.19, 7.87) | 0.04 | 0.95 | (-1.87, 3.77) | 0.51 |
| Age 35-49 | 358 | 13.35 | (10.16, 16.63) | <0.0001 | 6.36 | (5.16, 7.56) | <0.0001 | 699 | (173, 1,224) | 0.01 | 3.85 | (-0.25, 7.95) | 0.07 | -1.11 | (-4.05, 1.83) | 0.46 |
| Age 50+ | 82 | 17.61 | (12.55, 22.89) | <0.0001 | 9.18 | (7.33, 11.03) | <0.0001 | -9 | (-832, 813) | 0.98 | -5.98 | (-12.43, 0.47) | 0.07 | -2.04 | (-6.79, 2.72) | 0.40 |
| **Ethnic group** | | | | | | | | | | | | | | | | |
| White (Ref) | 595 | | | - | | | - | | | - | | | - | | | - |
| Black | 314 | 6.23 | (3.26, 9.28) | <0.0001 | 3.63 | (2.44, 4.83) | <0.0001 | -1,116 | (-1,657, -575) | <0.0001 | -7.42 | (-11.68, -3.17) | <0.001 | -6.61 | (-9.82, -3.41) | <0.0001 |
| Asian | 210 | -0.28 | (-3.14, 2.67) | 0.85 | 0.02 | (-1.20, 1.25) | 0.97 | -1,409 | (-1,972, -845) | <0.0001 | -11.46 | (-15.89, -7.03) | <0.0001 | -8.11 | (-11.43, -4.79) | <0.0001 |
| Other/Mixed | 121 | 1.29 | (-2.27, 4.98) | 0.48 | 1.04 | (-0.47, 2.55) | 0.18 | -430 | (-1,100, 239) | 0.21 | -4.57 | (-9.83, 0.69) | 0.09 | -3.95 | (-7.86, -0.04) | 0.05 |
| **Limiting illness** | | | | | | | | | | | | | | | | |
| No (Ref) | 1087 | | | - | | | - | | | - | | | - | | | - |
| Yes | 153 | 4.29 | (1.13, 7.55) | 0.01 | 1.63 | (0.33, 2.92) | 0.01 | -1,081 | (-1,666, -496) | <0.001 | -5.69 | (-10.27, -1.12) | 0.01 | -2.78 | (-6.10, 0.55) | 0.10 |
| **Housing sector** | | | | | | | | | | | | | | | | |
| Social | 512 | 4.96 | (2.21, 7.78) | <0.001 | 2.66 | (1.54, 3.78) | <0.0001 | -1,125 | (-1,629, -620) | <0.0001 | -7.53 | (-11.50, -3.55) | <0.001 | -6.49 | (-9.50, -3.48) | <0.0001 |
| Intermediate (Ref) | 503 | | | - | | | - | | | - | | | - | | | - |
| Market-rent | 225 | -0.81 | (-3.56, 2.02) | 0.57 | -0.23 | (-1.42, 0.96) | 0.70 | -104 | (-633, 424) | 0.70 | 2.26 | (-1.90, 6.42) | 0.29 | 2.82 | (-0.35, 5.98) | 0.08 |

* Percentage differences are presented for log transformed variables
All differences/% differences are mutually adjusted for sex, age group, ethnic group, limiting longstanding illness, housing sector and a random effect to allow for clustering at household level
† Missing data for 133 participants



For peer review only

For peer review only - http://bmjopen.bmj.com/site/about/guidelines.xhtml

Table 2: Associations between neighbourhood perceptions scales and adiposity and physical activity

| | Difference / % difference in outcome between the highest and lowest quintiles for each neighbourhood factor (95% CI), p-value | | | | | |
|---|---|---|---|---|---|---|
| Adiposity (N = 1240) | Perceptions of NH quality scale | | | Perceptions of NH crime scale | | |
| Body mass index (kg/m$^2$)* | -3.58 | (-6.47, -0.60) | 0.02 | -2.15 | (-5.45, 1.26) | 0.21 |
| Fat mass % | -1.23 | (-2.51, 0.06) | 0.06 | -0.76 | (-2.20, 0.69) | 0.30 |
| Physical activity (N = 1107) | | | | | | |
| Steps | 677.49 | (107.99, 1,246.99) | 0.02 | -63.05 | (-713.40, 587.31) | 0.85 |
| MVPA (minutes) | 4.49 | (0.02, 8.95) | 0.05 | 1.06 | (-4.03, 6.16) | 0.68 |
| MVPA in 10 minute bouts (minutes) | 2.67 | (-0.63, 5.97) | 0.11 | 2.37 | (-1.39, 6.13) | 0.22 |

* % differences are shown for log transformed variables
All differences/% differences are adjusted for sex, age group, ethnic group, limiting longstanding illness, housing sector and a random effect to allow for clustering at household level.
Abbreviations: NH, neighbourhood

For peer review only



For peer review only - http://bmjopen.bmj.com/site/about/guidelines.xhtml

Table 3: Body size, adiposity and physical activity differences between housing sectors: adjustment for perceptions of neighbourhood quality

| Adiposity (N = 1240) | Housing sector group | Difference / % difference* compared to intermediate housing group (95% confidence interval), p-value | | | | | |
|---|---|---|---|---|---|---|---|
| | | Model 1 | | | Model 2 (Additionally adjusted for neighbourhood quality scale) | | |
| Body mass index (kg/m$^2$)* | Social | 4.96 | (2.21, 7.78) | <0.001 | 4.45 | (1.67, 7.31) | 0.002 |
| | Intermediate | Reference group | | | | | |
| | Market rent | -0.81 | (-3.56, 2.02) | 0.57 | -0.85 | (-3.60, 1.98) | 0.55 |
| Fat mass % | Social | 2.66 | (1.54, 3.78) | <0.0001 | 2.49 | (1.35, 3.63) | <0.0001 |
| | Intermediate | Reference group | | | | | |
| | Market rent | -0.23 | (-1.42, 0.96) | 0.70 | -0.25 | (-1.43, 0.94) | 0.68 |
| Physical activity (N = 1107) | | | | | | | |
| Steps | Social | -1,125 | (-1,629, -620) | <0.0001 | -1,016 | (-1,531, -501) | <0.001 |
| | Intermediate | Reference group | | | | | |
| | Market rent | -104 | (-633, 424) | 0.70 | -96 | (-624, 431) | 0.72 |
| MVPA (minutes) | Social | -7.53 | (-11.50, -3.55) | <0.001 | -6.76 | (-10.81, -2.71) | 0.001 |
| | Intermediate | Reference group | | | | | |
| | Market rent | 2.26 | (-1.90, 6.42) | 0.29 | 2.32 | (-1.84, 6.47) | 0.27 |
| MVPA in 10 minute bouts (minutes) | Social | -6.49 | (-9.50, -3.48) | <0.0001 | -6.03 | (-9.10, -2.95) | <0.001 |
| | Intermediate | Reference group | | | | | |
| | Market rent | 2.82 | (-0.35, 5.98) | 0.08 | 2.85 | (-0.31, 6.01) | 0.08 |

* % differences are shown for log transformed variables
Model 1 is adjusted for sex, age group, ethnic group, limiting longstanding illness and clustering at household level (random effect)
Model 2: Adjusted for Model 1 plus perceived neighbourhood quality

For peer review only

Figure 1: Daily physical activity by day of the week and housing sector group: N = 6206 days from 1107 participants

Means and 95% confidence intervals are adjusted for sex, age group, ethnic group, limiting longstanding illness, month of recording, day order of recording, day of week, housing sector, an interaction between housing sector and day of week and random effects to allow for multiple days of measurement and clustering of participants within households

For peer review only



For peer review only - http://bmjopen.bmj.com/site/about/guidelines.xhtml

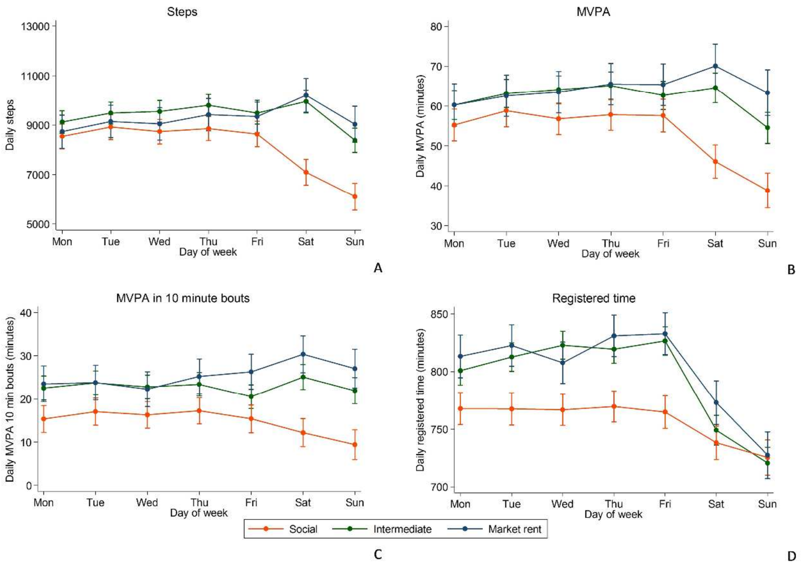


514x309mm (300 x 300 DPI)

## Supplementary material

Supplementary Table 1: Questionnaire items included the factor analysis on perceptions of the neighbourhood

| |
|---|
| **Perceptions of neighbourhood crime items** |
| There is a lot of crime in my neighbourhood. |
| The level of crime in my neighbourhood makes it unsafe to walk on the streets at night. |
| There are threatening groups of young people in my neighbourhood. |
| The level of crime in my neighbourhood makes it unsafe to walk on the streets during the day. |
| Vandalism, graffiti or deliberate damage to property is a problem in my local area. |
| **Perceptions of neighbourhood quality items** |
| I enjoy walking in my neighbourhood. |
| This area is a place I enjoy living in. |
| My neighbourhood is attractive to look at (e.g. there are attractive buildings, green space. Landscaping views). |
| This area has good leisure things for people like myself, leisure centres or community centres for example. |
| You often see people out on walks or riding their bicycles in my neighbourhood. |
| This area has good local transport. |
| **Additional items included in the factor analysis with factor loadings below 0.4 and were therefore not included in the solution** |
| My neighbourhood is generally free from litter. |
| There is too much traffic in my neighbourhood. |
| Our neighbourhood streets have good lighting at night. |

Participants were asked to select a response from the following for all questionnaire items stated in the table:- Strongly agree, Agree, Neither agree nor disagree, Disagree, Strongly disagree.



For peer review only - http://bmjopen.bmj.com/site/about/guidelines.xhtml

Supplementary Table 2: Participant characteristics for 1240 adults with measurements of adiposity at baseline

| | Social (n = 512) | | Housing sector<br>Intermediate (n = 503) | | Private (n = 225) | | Total (N = 1240) | | p ($\chi^2$) |
|---|---|---|---|---|---|---|---|---|---|
| **Sex** | | | | | | | | | |
| Male | 137 | (26.8%) | 259 | (51.5%) | 126 | (56.0%) | 522 | (42.1%) | |
| Female | 375 | (73.2%) | 244 | (48.5%) | 99 | (44.0%) | 718 | (57.9%) | <0.0001 |
| **Age group** | | | | | | | | | |
| 16-24 | 107 | (20.9%) | 92 | (18.3%) | 70 | (31.1%) | 269 | (21.7%) | |
| 25-34 | 129 | (25.2%) | 291 | (57.9%) | 111 | (49.3%) | 531 | (42.8%) | |
| 35-49 | 233 | (45.5%) | 102 | (20.3%) | 23 | (10.2%) | 358 | (28.9%) | |
| 50+ | 43 | (8.4%) | 18 | (3.6%) | 21 | (9.3%) | 82 | (6.6%) | <0.0001 |
| **Ethnic group** | | | | | | | | | |
| White | 96 | (18.8%) | 342 | (68.0%) | 157 | (69.8%) | 595 | (48.0%) | |
| Black | 245 | (47.9%) | 53 | (10.5%) | 16 | (7.1%) | 314 | (25.3%) | |
| Asian | 107 | (20.9%) | 75 | (14.9%) | 28 | (12.4%) | 210 | (16.9%) | |
| Mixed/Other | 64 | (12.5%) | 33 | (6.6%) | 24 | (10.7%) | 121 | (9.8%) | <0.0001 |
| **NS-SEC*** | | | | | | | | | |
| Higher Managerial / Professional | 60 | (11.9%) | 357 | (71.4%) | 150 | (66.7%) | 567 | (46.1%) | |
| Intermediate Occupations | 62 | (12.3%) | 77 | (15.4%) | 38 | (16.9%) | 177 | (14.4%) | |
| Routine / Manual | 125 | (24.8%) | 34 | (6.8%) | 10 | (4.4%) | 169 | (13.7%) | |
| Economically inactive | 258 | (51.1%) | 32 | (6.4%) | 27 | (12.0%) | 317 | (25.8%) | <0.0001 |
| **Limiting illness** | | | | | | | | | |
| Yes | 102 | (19.9%) | 40 | (8.0%) | 11 | (4.9%) | 153 | (12.3%) | |
| No | 410 | (80.1%) | 463 | (92.0%) | 214 | (95.1%) | 1087 | (87.7%) | <0.0001 |

p ($\chi^2$): p-value for Chi-squared test
* 10 responses missing for NS-SEC group



For peer review only - http://bmjopen.bmj.com/site/about/guidelines.xhtml

Supplementary Table 3: Mean levels of adiposity and physical activity by participant characteristics

| | n | Mean/Geometric mean* levels adiposity and physical activity | | | | | | | | | |
|---|---|---|---|---|---|---|---|---|---|---|---|
| | | BMI (kg/m$^2$)* | | Fat mass % | | Steps† | | Minutes spent in MVPA† | | Minutes spent in MVPA in 10 minute bouts† | |
| **Sex** | | | | | | | | | | | |
| Male | 522 | 25.4 | (25.0, 25.8) | 20.4 | (19.7, 21.0) | 9,279 | (8,991, 9,568) | 64.8 | (62.6, 67.1) | 22.8 | (21.1, 24.4) |
| Female | 718 | 25.1 | (24.8, 25.5) | 31.5 | (30.9, 32.0) | 8,709 | (8,464, 8,954) | 55.6 | (53.6, 57.5) | 18.7 | (17.3, 20.1) |
| **Age group** | | | | | | | | | | | |
| Age 16-24 | 269 | 23.5 | (23.0, 24.0) | 23.0 | (22.1, 23.9) | 8,534 | (8,136, 8,932) | 57.0 | (53.9, 60.2) | 20.5 | (18.2, 22.8) |
| Age 25-34 | 531 | 25.0 | (24.6, 25.3) | 26.2 | (25.5, 26.8) | 9,035 | (8,744, 9,326) | 61.1 | (58.8, 63.3) | 21.4 | (19.7, 23.1) |
| Age 35-49 | 358 | 26.6 | (26.1, 27.1) | 29.3 | (28.5, 30.1) | 9,232 | (8,879, 9,585) | 60.9 | (58.1, 63.6) | 19.4 | (17.3, 21.4) |
| Age 50+ | 82 | 27.6 | (26.6, 28.7) | 32.2 | (30.5, 33.8) | 8,525 | (7,800, 9,249) | 51.1 | (45.4, 56.7) | 18.4 | (14.2, 22.7) |
| **Ethnic group** | | | | | | | | | | | |
| White | 595 | 24.9 | (24.5, 25.2) | 25.8 | (25.1, 26.4) | 9,491 | (9,203, 9,779) | 63.6 | (61.3, 65.8) | 23.7 | (22.0, 25.4) |
| Black | 314 | 26.4 | (25.8, 27.0) | 29.4 | (28.5, 30.3) | 8,375 | (7,961, 8,789) | 56.2 | (52.9, 59.4) | 17.1 | (14.7, 19.6) |
| Asian | 210 | 24.8 | (24.2, 25.4) | 25.8 | (24.8, 26.8) | 8,082 | (7,608, 8,556) | 52.1 | (48.4, 55.8) | 15.6 | (12.8, 18.4) |
| Other/Mixed | 121 | 25.2 | (24.4, 26.0) | 26.8 | (25.5, 28.1) | 9,060 | (8,465, 9,656) | 59.0 | (54.3, 63.7) | 19.8 | (16.3, 23.3) |
| **Limiting illness** | | | | | | | | | | | |
| No | 1087 | 25.1 | (24.9, 25.4) | 26.6 | (26.1, 27.0) | 9,077 | (8,877, 9,277) | 60.1 | (58.6, 61.7) | 20.8 | (19.6, 21.9) |
| Yes | 153 | 26.2 | (25.5, 27.0) | 28.2 | (27.0, 29.4) | 7,996 | (7,447, 8,545) | 54.4 | (50.1, 58.7) | 18.0 | (14.8, 21.1) |
| **Housing sector** | | | | | | | | | | | |
| Social | 512 | 26.0 | (25.6, 26.5) | 28.4 | (27.6, 29.1) | 8,298 | (7,953, 8,642) | 54.6 | (51.8, 57.3) | 16.0 | (14.0, 18.1) |
| Intermediate | 503 | 24.8 | (24.4, 25.2) | 25.7 | (25.0, 26.4) | 9,422 | (9,110, 9,735) | 62.1 | (59.6, 64.5) | 22.5 | (20.6, 24.4) |
| Market-rent | 225 | 24.6 | (24.0, 25.2) | 25.5 | (24.5, 26.5) | 9,318 | (8,863, 9,773) | 64.3 | (60.8, 67.9) | 25.3 | (22.6, 28.1) |

* Geometric means are presented for log transformed variables
All means/geometric means are adjusted for sex, age group, ethnic group, limiting longstanding illness, housing sector and a random effect to allow for clustering at household level
† Missing data for 133 participants



For peer review only - http://bmjopen.bmj.com/site/about/guidelines.xhtml

For peer review only

Supplementary Table 4: Associations between participant characteristics and physical activity variables in participants with at least 4 days of recording of physical activity data

| | | Difference/% difference* in physical activity variable | | | | | | | | |
|---|---|---|---|---|---|---|---|---|---|---|
| | n | Steps | | | Minutes spent in MVPA | | | Minutes spent in MVPA in 10 minute bouts | | |
| **Sex** | | | | | | | | | | |
| Male (Ref) | 402 | | - | | | - | | | - | |
| Female | 529 | -559.1 | (-946.6, -171.7) | 0.005 | -9.73 | (-12.75, -6.71) | <0.0001 | -4.65 | (-6.78, -2.53) | <0.0001 |
| **Age group** | | | | | | | | | | |
| Age 16-24 (Ref) | 180 | | - | | | - | | | - | |
| Age 25-34 | 412 | 409.9 | (-115.7, 935.6) | 0.13 | 3.01 | (-1.15, 7.16) | 0.16 | -0.26 | (-3.28, 2.77) | 0.87 |
| Age 35-49 | 276 | 520.2 | (-45.2, 1,085.6) | 0.07 | 2.48 | (-1.96, 6.92) | 0.27 | -2.50 | (-5.67, 0.68) | 0.12 |
| Age 50+ | 63 | -25.5 | (-889.1, 838.1) | 0.95 | -7.94 | (-14.78, -1.10) | 0.02 | -3.23 | (-8.22, 1.76) | 0.20 |
| **Ethnic group** | | | | | | | | | | |
| White (Ref) | 482 | | - | | | - | | | - | |
| Black | 214 | -1,213.5 | (-1,788.9, -638.0) | <0.0001 | -7.08 | (-11.67, -2.49) | 0.002 | -6.46 | (-9.87, -3.06) | <0.001 |
| Asian | 142 | -1,128.3 | (-1,718.8, -537.7) | <0.001 | -10.29 | (-14.99, -5.59) | <0.0001 | -7.79 | (-11.26, -4.32) | <0.0001 |
| Other/Mixed | 93 | -581.5 | (-1,273.4, 110.4) | 0.10 | -4.73 | (-10.21, 0.76) | 0.09 | -4.20 | (-8.21, -0.18) | 0.04 |
| **Limiting illness** | | | | | | | | | | |
| No (Ref) | 834 | | - | | | - | | | - | |
| Yes | 97 | -976.1 | (-1,611.5, -340.6) | 0.003 | -4.63 | (-9.63, 0.37) | 0.07 | -1.98 | (-5.59, 1.63) | 0.28 |
| **Housing sector** | | | | | | | | | | |
| Social | 332 | -977.5 | (-1,514.6, -440.5) | <0.001 | -6.87 | (-11.16, -2.58) | 0.002 | -7.21 | (-10.40, -4.01) | <0.0001 |
| Intermediate (Ref) | 410 | | - | | | - | | | - | |
| Market-rent | 189 | -358.7 | (-888.8, 171.4) | 0.18 | 0.24 | (-4.00, 4.48) | 0.91 | 0.94 | (-2.23, 4.12) | 0.56 |

All differences are mutually adjusted for sex, age group, ethnic group, limiting longstanding illness, housing sector and a random effect to allow for clustering at household level.



For peer review only - http://bmjopen.bmj.com/site/about/guidelines.xhtml

For peer review only

Supplementary Table 5: Physical Activity differences between weekday (Monday-Friday) and weekend (Saturday, Sunday) activity: by housing sector

| Physical activity variable (N = 1107) | Housing sector group | Mean (95% CI) weekday (Mon-Fri) activity | | Difference in PA outcome compared to weekdays (95% confidence interval), p-value: Saturday - weekday | | | Sunday - weekday | | |
|---|---|---|---|---|---|---|---|---|---|
| Steps | Social | 8,733 | (8,364, 9,103) | -1,643 | (-2,078, -1,207) | <0.0001 | -2,629 | (-3,093, -2,164) | <0.0001 |
| | Intermediate | 9,497 | (9,178, 9,817) | 460 | (59, 862) | 0.02 | -1,104 | (-1,528, -680) | <0.0001 |
| | Market-rent | 9,146 | (8,673, 9,619) | 1,055 | (467, 1,642) | <0.001 | -102 | (-734, 531) | 0.75 |
| MVPA (minutes) | Social | 57.2 | (54.3, 60.1) | -11.2 | (-14.7, -7.7) | <0.0001 | -18.4 | (-22.1, -14.7) | <0.0001 |
| | Intermediate | 63.1 | (60.6, 65.7) | 1.5 | (-1.8, 4.7) | 0.37 | -8.5 | (-11.9, -5.1) | <0.0001 |
| | Market-rent | 63.5 | (59.8, 67.3) | 6.6 | (1.9, 11.3) | 0.01 | -0.1 | (-5.2, 5.0) | 0.97 |
| MVPA in 10 minute bouts (minutes) | Social | 16.3 | (14.0, 18.5) | -4.1 | (-6.9, -1.3) | 0.004 | -6.8 | (-9.8, -3.9) | <0.0001 |
| | Intermediate | 22.6 | (20.7, 24.6) | 2.5 | (-0.1, 5.1) | 0.06 | -0.7 | (-3.4, 2.0) | 0.62 |
| | Market-rent | 24.2 | (21.3, 27.1) | 6.1 | (2.4, 9.9) | 0.001 | 2.8 | (-1.2, 6.9) | 0.17 |

Means and differences (95% CIs) are adjusted for sex, age group, ethnic group, limiting longstanding illness, month of recording, day order of recording, day of the week, housing sector, an interaction between housing sector and day of week and random effects to allow for multiple days of measurement and clustering of participants within household.



For peer review only - http://bmjopen.bmj.com/site/about/guidelines.xhtml

**STROBE 2007 (v4) Statement—Checklist of items that should be included in reports of *cross-sectional studies***

| Section/Topic | Item # | Recommendation | Reported on page # |
|---|---|---|---|
| **Title and abstract** | 1 | (*a*) Indicate the study's design with a commonly used term in the title or the abstract | 1 |
| | | (*b*) Provide in the abstract an informative and balanced summary of what was done and what was found | 2 |
| **Introduction** | | | |
| Background/rationale | 2 | Explain the scientific background and rationale for the investigation being reported | 4-5 |
| Objectives | 3 | State specific objectives, including any prespecified hypotheses | 6 |
| **Methods** | | | |
| Study design | 4 | Present key elements of study design early in the paper | 6-7 |
| Setting | 5 | Describe the setting, locations, and relevant dates, including periods of recruitment, exposure, follow-up, and data collection | 6-7 |
| Participants | 6 | (*a*) Give the eligibility criteria, and the sources and methods of selection of participants | 6-7 |
| Variables | 7 | Clearly define all outcomes, exposures, predictors, potential confounders, and effect modifiers. Give diagnostic criteria, if applicable | 7-8 |
| Data sources/ measurement | 8* | For each variable of interest, give sources of data and details of methods of assessment (measurement). Describe comparability of assessment methods if there is more than one group | 7-8 |
| Bias | 9 | Describe any efforts to address potential sources of bias | *9* |
| Study size | 10 | Explain how the study size was arrived at | 6-7 |
| Quantitative variables | 11 | Explain how quantitative variables were handled in the analyses. If applicable, describe which groupings were chosen and why | 9 |
| Statistical methods | 12 | (*a*) Describe all statistical methods, including those used to control for confounding | 9-10 |
| | | (*b*) Describe any methods used to examine subgroups and interactions | 9-10 |
| | | (*c*) Explain how missing data were addressed | N/A |
| | | (*d*) If applicable, describe analytical methods taking account of sampling strategy | 9-10 |
| | | (*e*) Describe any sensitivity analyses | 9 |
| **Results** | | | |

For peer review only

| | | | |
|---|---|---|---|
| Participants | 13* | (a) Report numbers of individuals at each stage of study—eg numbers potentially eligible, examined for eligibility, confirmed eligible, included in the study, completing follow-up, and analysed | 10 |
| | | (b) Give reasons for non-participation at each stage | 10 |
| | | (c) Consider use of a flow diagram | N/A |
| Descriptive data | 14* | (a) Give characteristics of study participants (eg demographic, clinical, social) and information on exposures and potential confounders | 10-11 |
| | | (b) Indicate number of participants with missing data for each variable of interest | 10 |
| Outcome data | 15* | Report numbers of outcome events or summary measures | 10-11 |
| Main results | 16 | (*a*) Give unadjusted estimates and, if applicable, confounder-adjusted estimates and their precision (eg, 95% confidence interval). Make clear which confounders were adjusted for and why they were included | 10-11 |
| | | (*b*) Report category boundaries when continuous variables were categorized | 11 |
| | | (*c*) If relevant, consider translating estimates of relative risk into absolute risk for a meaningful time period | N/A |
| Other analyses | 17 | Report other analyses done—eg analyses of subgroups and interactions, and sensitivity analyses | 11 |
| **Discussion** | | | |
| Key results | 18 | Summarise key results with reference to study objectives | 13-14 |
| Limitations | 19 | Discuss limitations of the study, taking into account sources of potential bias or imprecision. Discuss both direction and magnitude of any potential bias | 16-17 |
| Interpretation | 20 | Give a cautious overall interpretation of results considering objectives, limitations, multiplicity of analyses, results from similar studies, and other relevant evidence | 17 |
| Generalisability | 21 | Discuss the generalisability (external validity) of the study results | 17 |
| **Other information** | | | |
| Funding | 22 | Give the source of funding and the role of the funders for the present study and, if applicable, for the original study on which the present article is based | 19-20 |

*Give information separately for cases and controls in case-control studies and, if applicable, for exposed and unexposed groups in cohort and cross-sectional studies.

**Note:** An Explanation and Elaboration article discusses each checklist item and gives methodological background and published examples of transparent reporting. The STROBE checklist is best used in conjunction with this article (freely available on the Web sites of PLoS Medicine at http://www.plosmedicine.org/, Annals of Internal Medicine at http://www.annals.org/, and Epidemiology at http://www.epidem.com/). Information on the STROBE Initiative is available at www.strobe-statement.org.

For peer review only

BMJ Open

# Housing, neighbourhood and sociodemographic associations with adult levels of physical activity and adiposity: baseline findings from the ENABLE London Study

| | |
|---|---|
| Journal: | *BMJ Open* |
| Manuscript ID | bmjopen-2017-021257.R1 |
| Article Type: | Research |
| Date Submitted by the Author: | 16-May-2018 |
| Complete List of Authors: | Nightingale, C; St George's University London, Division of Population Health Sciences and Education<br>Rudnicka, Alicja; SGUL University of London<br>Ram, Bina; St George's, University of London, Population Health Research Institute<br>Shankar, Aparna; St. Geoege's, University of London, Population Health Research Institute<br>Limb, Elizabeth; St George's University, Population Health Research Institute<br>Procter, Duncan; University of Bristol, Centre for Exercise, Nutrition and Health Sciences<br>Cooper, Ashley; University of Bristol,<br>Page, Angie; University of Bristol, Centre for Exercise, Nutrition and Health Sciences<br>Ellaway, Anne; University of Glasgow, MRC/SCO Social and Public Health Sciences Unit<br>Giles-Corti, Billie; RMIT University, NHMRC Centre of Research Excellence in Healthy Liveable Communities<br>Clary, Christelle; London School of Hygiene and Tropical Medicine, Social And Environmental Health Research<br>Lewis, Daniel; London School of Hygiene and Tropical Medicine,<br>Cummins, Steven; London School of Hygiene and Tropical Medicine, Department of Social and Environmental Health Research<br>Whincup, Peter; St Georges, University of London<br>Cook, Derek; St George's University, Division of Population Health Sciences<br>Owen, C; St George's, University London, Population Health Research Institute |
| <b>Primary Subject Heading</b>: | Epidemiology |
| Secondary Subject Heading: | Cardiovascular medicine |
| Keywords: | Physical activity, Adiposity, Housing, Perceived neighbourhood environment, ENABLE-London |
| | |

For peer review only - http://bmjopen.bmj.com/site/about/guidelines.xhtml

For peer review only

# Housing, neighbourhood and sociodemographic associations with adult levels of physical activity and adiposity: baseline findings from the ENABLE London Study

CM Nightingale[1], AR Rudnicka[1], B Ram[1], A Shankar[1], ES Limb[1], D Procter[2,3], AR Cooper[2,3], AS Page[2,3], A Ellaway[4], B Giles-Corti[5], C Clary[6], D Lewis[6], S Cummins[6], PH Whincup[1], DG Cook[1], CG Owen[1]

[1] Population Health Research Institute, St George's, University of London, London, UK.

[2] Centre for Exercise, Nutrition and Health Sciences, University of Bristol, Bristol, UK.

[3] National Institute for Health Research Bristol Biomedical Research Centre, University Hospitals Bristol NHS Foundation Trust and University of Bristol, UK.

[4] MRC/SCO Social and Public Health Sciences Unit, University of Glasgow, Glasgow, UK.

[5] NHMRC Centre of Research Excellence in Healthy Liveable Communities, RMIT University, Melbourne, Australia.

[6] London School of Hygiene and Tropical Medicine, London, UK.

Corresponding Author:-

Dr Claire M Nightingale

Population Health Research Institute, St. George's, University of London, Cranmer Terrace, London SW17 0RE. Email: cnightin@sgul.ac.uk. Phone: +44 (0) 208 725 2342

only

**Total word count:** 4551 excluding abstract (311 words)

**Tables and figures:** 3 tables, 1 figure, 5 supplementary tables

**References:** 53



For peer review only - http://bmjopen.bmj.com/site/about/guidelines.xhtml

## *Abstract*

**Objectives:** The neighbourhood environment is increasingly shown to be an important correlate of health. We assessed associations between housing tenure, neighbourhood perceptions, sociodemographic factors, and levels of physical activity (PA) and adiposity among adults seeking housing in East Village (formerly London 2012 Olympic/Paralympic Games Athletes' Village).

**Setting:** Cross-sectional analysis of adults seeking social, intermediate and market-rent housing in East Village.

**Participants:** 1278 participants took part in the study (58% female). Complete data on adiposity (body mass index [BMI] and fat mass %) were available for 1240 participants (97%); of these a sub-set of 1107 participants (89%) met the inclusion criteria for analyses of accelerometer-based measurements of PA. We examined associations between housing sector sought, neighbourhood perceptions (covariates) and PA and adiposity (dependent variables) adjusted for household clustering, sex, age group, ethnic group, and limiting longstanding illness.

**Results:** Participants seeking social housing had the fewest daily steps (8304, 95%CI 7959,8648) and highest BMI (26.0kg/$m^2$ 95%CI 25.5,26.5kg/$m^2$) compared with those seeking intermediate (daily steps 9417, 95%CI 9106,9731; BMI 24.8kg/$m^2$ 95%CI 24.4,25.2kg/$m^2$) or market-rent housing (daily steps 9313, 95%CI 8858,9768; BMI 24.6kg/$m^2$ 95%CI 24.0,25.2kg/$m^2$). Those seeking social housing had lower levels of PA (by 19-42%) at weekends vs weekdays, compared with other housing groups. Positive perceptions of neighbourhood quality were associated with higher steps and lower BMI, with differences between social and intermediate groups reduced by ~10% following adjustment, equivalent to a reduction of 111 for steps and 0.5kg/$m^2$ for BMI.



For peer review only - http://bmjopen.bmj.com/site/about/guidelines.xhtml

**Conclusions:** The social housing group undertook less PA than other housing sectors, with weekend PA offering the greatest scope for increasing PA, and tackling adiposity in this group. Perceptions of neighbourhood quality were associated with PA and adiposity and reduced differences in steps and BMI between housing sectors. Interventions to encourage physical activity at weekends and improve neighbourhood quality, especially amongst the most disadvantaged, may provide scope to reduce inequalities in health behaviour.

**Strengths and limitations of this study**

- Large sample with representation of three different aspirational housing groups, providing a wide range of socioeconomic backgrounds
- Objective measurements of physical activity and adiposity outcomes using accelerometry and bioelectrical impedance respectively
- Lower number of participants studied seeking market-rent housing compared with those seeking intermediate or social housing

**Keywords**

Physical activity; Adiposity; Housing; Perceived neighbourhood environment; ENABLE-London

For peer review only



For peer review only - http://bmjopen.bmj.com/site/about/guidelines.xhtml

## *Introduction*

Physical inactivity and adiposity are associated with an increased risk of type 2 diabetes and cardiovascular disease (1-4) and constitute a serious public health problem in the UK and globally (5). Evidence suggests that levels of physical activity (PA) are lower among those who are socioeconomically disadvantaged (6), who experience greater economic, access and health related barriers to being physically active (7). Socioeconomic status is also associated with differences in types of PA, in particular higher socioeconomic status is associated with more vigorous leisure time PA (8). Previous research has found variation in PA by day of the week with studies showing lower levels of activity on Sundays compared with weekdays in young adults (9), parents and their children (10).

There is emerging evidence suggesting that housing tenure is an important determinant of health. In particular, UK-based studies have shown that housing tenure (owner vs. private renter vs. public sector renter) is associated with illness and mortality (15;16). Amongst particular groups including those who are economically inactive or unemployed, housing tenure might provide a better indication of socioeconomic status compared with measures based on occupation or income (11). Indeed, in several studies housing tenure remained associated with health outcomes following adjustment for conventional measures of socioeconomic status such as income or education (12;13). A more nuanced approach is therefore required with respect to measures of socioeconomic status, and they should not be simply regarded as interchangeable (14;15). Despite this, there has been limited research examining the direct effect of housing tenure on PA, and existing evidence is equivocal. Harrison and colleagues found no association between housing tenure and meeting recommended levels of PA among community dwelling healthy adults in the North-East of


For peer review only - http://bmjopen.bmj.com/site/about/guidelines.xhtml

England (16). Similarly housing tenure was not associated with self-reported energetic PA among older Australians (17). Ogilvie and colleagues found overall levels of PA to be higher among individuals living in social housing compared with owner-occupiers (18). The authors suggest that may capture occupational PA levels which are likely to be higher among those in social housing (18). In contrast, living in private rental accommodation was associated with a greater likelihood of taking up exercise over a 9-year period among men aged 18-49 at baseline, compared with those in local authority accommodation (19).

Housing tenure may affect health and health behaviours in part through characteristics of the home or neighbourhood itself (20;21) or psychological factors such as self-efficacy or self-esteem (22). Social housing estates which are common in the UK may be associated with specific cultures and norms, which in turn shape residents' behaviours (13). Subjective characteristics of the neighbourhood environment including higher perceived access to recreational facilities and shops in local proximity have been shown to be associated with higher levels of PA (23;24). Residents who perceive their neighbourhood more positively, have been shown to have better mental health and are less likely to relocate (25). Conversely, real and perceived crime, has the potential to constrain residents' PA (26). However, a recent systematic review suggested a lack of association between PA and perceptions of safety from crime; highlighting the need for high quality evidence, including prospective studies and natural experiments (27), to examine this issue further. In particular, high quality evidence is needed to understand the potentially multifactorial influence of residential location on health and health behaviours; effects which are likely to extend beyond simple measures of socioeconomic status (27).


For peer review only - http://bmjopen.bmj.com/site/about/guidelines.xhtml

The Examining Neighbourhood Activities and Built Living Environments in London (ENABLE London) study is a longitudinal study evaluating how active urban design influences the health and wellbeing of people moving into the former Athletes' Village of the London 2012 Olympic and Paralympic Games now known as 'East Village' (28). East Village is a new high-density neighbourhood development built on active design principles containing a mix of social housing, intermediate (including affordable rent, shared ownership and shared equity) housing, and market-rent housing. This paper draws on baseline data (prior to any potential move to East Village) to first, examine predictors of PA and adiposity (measured objectively using accelerometry and bioelectrical impedance), including the housing sector to which they are applying and perceptions of their neighbourhood. Second, to examine whether PA patterns across the week vary by housing sector and third, to examine whether adjustment for perceptions of the neighbourhood environment reduce housing sector differences in PA and adiposity.

## *Methods*

Study participants were recruited from those seeking or who had applied for new accommodation in East Village and were classified by the type of housing tenure sought based on level of income; i.e. social, intermediate or market-rent. The inclusion criteria was broad and included anyone interested / applying for single or multiple occupancy accommodation in East Village. There was no explicit exclusion criteria; adults of any age, gender, ethnic group, with or without handicap, were invited to participate. Current housing status was strongly linked to aspirational housing status, where those seeking social accommodation were currently in social housing or on social housing waiting lists, and those seeking intermediate and market-rent accommodation were largely in privately rented


For peer review only - http://bmjopen.bmj.com/site/about/guidelines.xhtml

housing. Recruitment of participants in the different housing sectors was carried out between January 2013 and December 2015 in three phases determined by the order of availability of housing in East Village (social, intermediate, and market-rent respectively). Those applying for social housing in East Village were initially recruited between January 2013 and May 2014, households seeking intermediate accommodation between July 2013 and November 2014 and those seeking market rent accommodation between September 2014 and December 2015. Recruitment processes for those applying for social housing were slightly different compared with other housing sectors. The East Thames Group housing association was primarily responsible for recruiting participants in social housing, whereas the ENABLE London team (in association with Triathlon Homes and Get Living London) recruited participants from the other housing sectors (28). Aspirational housing tenure is integral to the design of ENABLE London, and we have shown that this provides a clear socioeconomic marker of study participants. For example, those seeking social housing in East Village are more likely to be unemployed, less educated and more likely to represent ethnic minorities (a classic marker of socioeconomic vulnerability), compared to those seeking affordable and market-rent accommodation (29). We have also shown key differences in mental health and well-being between housing groups, where those seeking social housing were more likely to be depressed, anxious and have poorer well-being, compared to other housing groups (30). Moreover, this is entirely consistent with earlier studies which found that both current housing tenure and aspirational housing tenure are associated with a variety of health outcomes, including mental health and measures of general health (31;32).


For peer review only - http://bmjopen.bmj.com/site/about/guidelines.xhtml

Baseline assessments of participants were carried out in their place of residence before any potential move to East Village. Full details of the recruitment process can be found elsewhere (28).

**Independent variables**

A team of trained fieldworkers administered self-complete questionnaires on a laptop during home visits. Data on age, sex, self-defined ethnicity, work status, occupation and whether the participant had a limiting longstanding illness or disability (lasting or expected to last at least 12 months) were collected. Participants self-defined as 'White', 'Asian', 'Black', 'Mixed', or 'Other'; the latter two categories were combined for analyses. Socioeconomic status based on occupation was coded using the National Statistics Social-Economic Coding (NS-SEC) to categorise participants into 'higher managerial or professional occupations', 'intermediate occupations', 'routine or manual' (33). An additional 'economically inactive' category included those seeking employment, unable to work due to disability or illness, retired, looking after home and family, and students. We sought information on educational attainment; participants were categorised into "Degree or equivalent / Higher", "Intermediate qualifications" (including A levels and GSCEs), and "Other / None" (including work-based or foreign qualifications). Participants completed questionnaires assessing neighbourhood perceptions (30). Five items assessed perceived crime (e.g., "There is a lot of crime in my neighbourhood"; Cronbach's $\alpha = 0.87$) and six items assessed neighbourhood quality (e.g. "This area is a place I enjoy living in"; Cronbach's $\alpha = 0.78$). Responses on items were summed and scores ranged from -10 to +10 for perceived crime and -12 to +12 for perceived quality, such that positive scores indicate less perceived crime and better neighbourhood quality while negative scores indicate more


For peer review only - http://bmjopen.bmj.com/site/about/guidelines.xhtml

perceived crime and poorer quality. The scales were derived following an exploratory factor analysis of 14 questions regarding neighbourhood (Supplementary Table 1).

**Dependent variables**

Height was measured to the last complete millimetre using a portable stadiometer; weight was measured to the nearest kilogram using a Tanita SC-240 Body Composition Analyzer (Tanita, Tokyo, Japan); body mass index (BMI) was derived as weight(kg)/height(m)$^2$. The Tanita SC-240 Body Composition Analyzer also measured leg-to-leg bioelectrical impedance from which fat free mass and fat mass were estimated. Fat mass percentage was calculated as fat mass (kg)/weight (kg)*100.

Participants wore a hip-mounted ActiGraph GT3X+ accelerometer during waking hours over a consecutive period of 7 days (ActiGraph LLC, Florida, USA). These accelerometers provided daily measures of steps, counts and time spent in moderate and vigorous PA (MVPA) using established cut-offs. Daily time spent in MVPA both overall and in ≥10 minute bouts in accordance with UK recommendations for PA (34) were assessed. The cut-point for moderate PA was defined as ≥1952 counts per minute (35). We excluded any days of recording where the amount of registered time accumulated was below 540 minutes (36). Non-wear periods were defined as a minimum length of 60 minutes, allowing for a 2-minute spike tolerance. Participants with at least one day of recording were retained in analyses. We fitted a multilevel linear model for each outcome to allow for repeated measurements of daily PA, by fitting participant as a random effect and adjusting for day of the week, day order of recording and month as fixed effects. Raw level one residuals were obtained from the model and a within person average value of each outcome variable was obtained by


For peer review only - http://bmjopen.bmj.com/site/about/guidelines.xhtml

averaging these raw residuals. The average of these raw residuals for each participant was added to the sample mean for that particular PA variable to derive an unbiased average level of each PA variable for each person.

**Statistical analysis**

All analyses were carried out using STATA/SE software (Stata/SE 14 for Windows; StataCorp LP, College Station, TX, USA). Outcome variables were inspected for normality and BMI was log transformed due to its skewed distribution. Multilevel linear regression models were fitted, mutually adjusted for housing sector and participant characteristics (sex, age group, ethnic group, and limiting longstanding illness) as fixed effects, with a random effect to allow for household clustering. Residuals did not show departure from linearity, suggesting that the model assumptions were appropriate. Absolute differences or percentage differences for log transformed outcomes (i.e. BMI) are presented by sex, age group, ethnic group, limiting longstanding illness and housing sector. Sensitivity analyses examined whether associations remained when the sample was restricted to 931 participants (84%) with at least four days of 540 or more minutes per day of recording.

To assess differences in PA by day of the week as opposed to overall levels of PA we took the following approach. Daily PA data were examined using multilevel models with random effects to allow for multiple days of recording within person and household clustering. An interaction between housing sector and day of the week was fitted and models were adjusted for sex, age group, ethnic group, limiting longstanding illness, day order of recording and month of measurement as fixed effects.


For peer review only - http://bmjopen.bmj.com/site/about/guidelines.xhtml

The associations between neighbourhood perception scales and adiposity and PA outcomes were examined. Each of the neighbourhood quality and crime scores were included in the models as quintiles, to examine the differences in outcomes between the top and bottom quintile. Finally, the effect of adjustment for neighbourhood perception on differences in adiposity and PA between housing sectors was examined. If associations between outcomes and neighbourhood perceptions appeared linear, models examining housing sector differences were additionally adjusted for neighbourhood perceptions as a continuous variable.

**Patient and Public Involvement**

The ENABLE London study was developed in partnership with a network of both local and regional stakeholders identified through our collaborator links to agencies, involved with the design, planning and management of large-scale accommodation developments. Locally these included local authorities (particularly Newham) and a number of housing associations, in particular Triathlon Homes, a partner organisation of housing associations, which manages social and intermediate homes in East Village. Participants have been involved in the study from an early stage to ensure assessments and participation remain relevant and enjoyable, to ensure the continued significance and potential generalisability of the work.

## *Results*

Of 1819 households who agreed to be contacted by the study team in order to receive further information about the ENABLE London study, 1278 adults from 1006 households (55%) participated in the study and completed a questionnaire. Participation rates for those


For peer review only - http://bmjopen.bmj.com/site/about/guidelines.xhtml

seeking market-rent and intermediate housing were 58% and 57% respectively and were slightly lower in the social group (52%). Complete data on adiposity were available for 1240 participants (97%); of these a sub-set of 1107 participants (89%) met the inclusion criteria for analyses of objectively measured PA. Participant characteristics (age, sex) and levels of adiposity were similar among those who did and did not provide PA data; however, participants from black and Asian ethnic groups were less likely to provide PA data. Supplementary Table 2 shows participants characteristics at baseline for the 1240 adults with measurements of adiposity at baseline. Those seeking social housing were more likely to be female, of older age, of non-white ethnicity, to have limiting longstanding illness, and be in routine / manual occupations or economically inactive compared to those seeking intermediate or market-rent housing.

Adjusted mean levels of adiposity and PA outcomes by housing sector and participant characteristics are shown in Supplementary Table 3. Table 1 shows housing sector and other participant characteristics associations with BMI and fat mass %, and objectively measured PA (steps, time spent in MVPA, time spent in MVPA in ≥10 minute bouts). Participants seeking social housing had markedly higher levels of BMI and fat mass % and markedly lower levels of steps, MVPA and MVPA in ≥10 minute bouts compared with those seeking intermediate housing, though there were no differences between those seeking market-rent and intermediate accommodation.

Fat mass % was higher in females than males though there was no difference in BMI (Table 1). BMI and fat mass % were higher among all older age groups compared with 16-24 year olds. Participants of black ethnicity had higher levels of BMI and fat mass % compared with


For peer review only - http://bmjopen.bmj.com/site/about/guidelines.xhtml

whites; there were no differences in BMI and fat mass % between Asian or other/mixed ethnic groups and whites. Those with a limiting longstanding illness had higher levels of both BMI and fat mass %. All PA measures were lower among females. Steps and MVPA were slightly higher in 25-34 year olds and steps were also higher among 35-49 year olds compared with 16-24 year olds; however, there were no age group differences for MVPA in ≥10 minute bouts. Participants of black and Asian ethnicities had lower levels of steps, MVPA and MVPA in ≥10 minute bouts compared to whites. Participants who reported having a limiting longstanding illness had lower levels of steps and MVPA, but not MVPA in ≥10 minute bouts. Educational attainment level was not associated with any of the outcomes once housing sector had been adjusted for and adjustment for educational attainment did not materially alter housing sector differences in adiposity or PA outcomes (data available from authors).

Sensitivity analyses for PA outcomes were carried out in 931 participants who wore an ActiGraph for at least four days with at least 540 minutes of recording per day (Supplementary Table 4). There were no differences between market-rent and intermediate groups (consistent with the main analysis presented in Table 1). Differences between social and intermediate groups were broadly similar with the results presented in Table 1 for the main analysis.

Differences in PA variables between housing groups were examined by day of the week to explore whether differences between groups were consistent across the week (Figure 1A-D). Levels of PA (steps (panel A), MVPA (panel B) and MVPA in ≥10 minute bouts (panel C)) were generally consistent across weekdays (Monday – Friday) among all groups. In the


For peer review only - http://bmjopen.bmj.com/site/about/guidelines.xhtml

intermediate group, steps were higher on Saturdays and lower on Sundays; MVPA and MVPA in ≥10 minute bouts were lower on Sundays but there was no difference on Saturdays compared to weekday activity. In the market-rent group, steps, MVPA and MVPA in ≥10 minute bouts were higher on Saturdays and similar to weekdays on Sundays. In the social group, steps, MVPA and MVPA in ≥10 minute bouts were on average lower on Saturdays and lower still on Sundays. Registered time (panel D) was lowest on average in the social group during weekdays, decreasing on Saturdays and Sundays. The intermediate and market-rent groups had higher levels of registered time during weekdays compared with the social group which decreased on average on Saturdays and Sundays (despite recording more steps and minutes in MVPA suggesting a higher intensity of activity). Mean levels of steps, MVPA, and MVPA in ≥10 minute bouts on weekdays and differences on Saturday and Sunday compared to weekdays are shown by housing sector in Supplementary Table 5. The marked differences in activity between weekdays and weekend days in the social group are not explained by differences in registered time (data available from authors).

Associations between perceived neighbourhood quality and crime scales and adiposity and PA outcomes are shown in Table 2, adjusted for the participant characteristics shown in Table 1. Participants with the most positive perceptions of neighbourhood quality (highest quintile) had lower BMI, higher steps and recorded longer durations of MVPA compared with those who had the most negative perceptions of neighbourhood quality (lowest quintile). There were no significant associations between perceptions of neighbourhood crime and adiposity or PA.


For peer review only - http://bmjopen.bmj.com/site/about/guidelines.xhtml

The effect of adjustment for perceived neighbourhood quality on differences in adiposity and PA between housing sector groups is presented in Table 3. All associations between perceived neighbourhood quality and crime and outcome variables were approximately linear and were therefore fitted as continuous variables in the model. In addition, associations between perceived neighbourhood quality and crime and outcome variables were similar across the three housing groups (all p>0.05). Adjustment for perceptions of neighbourhood quality reduced differences in BMI, fat mass %, steps, MVPA and MVPA in ≥10 minute bouts between the social and intermediate groups by 10%, 6%, 10%, 10% and 7% respectively. Differences between market-rent and intermediate groups in adiposity and PA variables were not statistically significant before or after adjustment. A larger proportion of the social-intermediate group differences in steps, MVPA and MVPA in ≥10 minute bouts on weekends was explained by adjustment for perceptions of neighbourhood quality (10%, 16% and 16% respectively) compared to the differences in steps, MVPA and MVPA in ≥10 minute bouts on weekdays which were reduced by 10%, 8% and 3% respectively (data not shown).

## *Discussion*

The results of this study showed that participants seeking social housing in East Village had lower levels of PA and higher levels of BMI and fat mass % compared with those seeking intermediate and market-rent housing, even when adjusted for demographic factors. In the social housing group, levels of PA were particularly low on weekends compared with weekdays possibly reflecting higher occupational PA and lower leisure time PA; weekday-weekend differences in PA were less marked among those seeking intermediate and market-rent housing. However, the lower registered time at weekends but higher MVPA


For peer review only - http://bmjopen.bmj.com/site/about/guidelines.xhtml

and steps suggests more intense activity at weekends in the intermediate and market-rent housing groups. These findings may inform targeted interventions to increase PA and reduce adiposity in different socioeconomic groups.

Positive associations between perceived neighbourhood quality and PA, BMI and fat mass % were also shown. Adjustment for differences in perceived neighbourhood quality reduced differences in PA and BMI by approximately 10% between social and intermediate housing groups; equivalent to a reduction of 111 for daily steps, 0.5 minutes for MVPA and 0.5kg/m$^2$ for BMI. However, a larger proportion of the difference in PA was apparent at weekends; equivalent to a reduction of 222 for daily steps and 2.2 minutes for MVPA.

**Relation to previous studies**

Studies have shown that lower socioeconomic status is associated with lower levels of PA (37;38), and that those from more socially deprived backgrounds have the most barriers to being physically active (7). Previous research examining the role of housing tenure is limited. Findings from this study showed marked differences in PA and adiposity between those seeking social, intermediate and market-rent housing. In particular, lower PA and higher adiposity in participants seeking social housing, a group which comprises a high proportion of people from more socioeconomically disadvantaged backgrounds (28). The higher levels of BMI and fat mass % in those seeking social housing compared with those seeking intermediate or market-rent housing is consistent with systematic reviews which have found an association between lower socioeconomic status and higher levels of adiposity, particularly in higher income countries and among women (39). While socioeconomic status is a strong determinant of housing status, to our knowledge this is the first study to


For peer review only - http://bmjopen.bmj.com/site/about/guidelines.xhtml

explicitly examine housing sector differences in objective PA and markers of adiposity levels (i.e. BMI and fat mass %). However, it is important to consider more broadly what these aspirational housing sector differences might represent. Related studies have shown that those in social housing are less likely to use active travel compared with owner occupiers (18), and that those in social housing and home owners with a mortgage are more likely to be obese and have higher levels of illness and disability compared to outright home owners, even after adjustment for other socioeconomic status markers (40). These latter findings suggest that the effect of home ownership may be more complex and cannot be simply explained by socioeconomic status. Neighbourhood quality may offer a potential partial explanation for these findings (41). In the present study perceptions of better neighbourhood quality were associated with PA whereas perceptions of crime were not. In contrast, a large UK-based study found that perceptions of feeling safe in the neighbourhood had the largest effect on levels of PA compared with perceptions of leisure facilities, sense of belonging or access to public transport or amenities (42). Another study in the US found that low perceived safety from crime was associated with lower levels of MVPA (43). However, a recent review concluded that higher quality evidence is needed, including prospective studies and natural experiments in areas of wide crime variability, in order to further understand the effect of crime on physical and mental health (27). Moreover, previous work has suggested that objective and perceived measures of the built environment correlate differently with physical activity levels, suggesting that these measures are assessing different dimensions of the built environment which relate differently to health behaviour (44).



For peer review only - http://bmjopen.bmj.com/site/about/guidelines.xhtml

For peer review only

Our findings showed that PA levels were particularly low on the weekend among those seeking social housing, which is consistent with findings from a systematic review which found that leisure-time PA (which may be more likely to occur on weekends) was lower amongst those from lower socioeconomic groups (8). This suggests that low-cost strategies to increase weekend PA may be particularly beneficial to more disadvantaged households. A free community-based program in Bogata Colombia, temporarily closed streets on Sundays to encourage PA amongst more disadvantaged local residents (45). A similar program has been trialled in the United States (46), however the effectiveness, longevity and generalisability of these programs to other socioeconomically deprived areas is yet to be established.

**Strengths and limitations**

Strengths of this study include the representation of three different aspirational housing groups which provides a wide range of socioeconomic backgrounds. Of those seeking social housing, two-thirds (67%) were currently living in social housing accommodation provided by the local authority or housing association; the remainder were largely currently living in privately rented accommodation with many on social housing waiting lists. Of those seeking intermediate or market-rent accommodation, almost two-thirds were living in privately rented accommodation (both 64%); the remainder were largely living with relatives or friends. The study sample is large with good representation from a 'hard to reach' group of social housing participants. Participation rates were high given the target group, with between 50-60% of those who initially agreed to be contacted taking part in the study. The ActiGraph GT3X+ accelerometer provided validated objective measures of PA (47) and the use of bioelectrical impedance to provide more direct measurements of adiposity including



For peer review only - http://bmjopen.bmj.com/site/about/guidelines.xhtml

For peer review only

fat mass %, which may provide a more valid marker of adiposity than BMI, particularly in a multi-ethnic population (48;49). Reassuringly the patterns of PA by sex, ethnic group and health status were consistent with those published previously (50-52). A limitation of the study is the lower number of participants in the market-rent sector compared with the other groups. This was due to restrictions imposed on the study team on the extent and duration of access to potential applicants seeking market-rent accommodation. While the study is longitudinal, these analyses are cross-sectional limiting the degree to which causal inferences can be made. Moreover, there is the possibility of selection amongst study participants, where those who are more active seek to move to East Village, may be more likely to participate in the study and may perceive their environment differently, which may limit the generalisability of the findings to neighbourhoods outside of East London.

**Conclusions and future work**

The findings presented in this paper suggest that perceived neighbourhood quality is associated with meaningful differences in PA and markers of adiposity. Differences in steps (680 steps) and BMI (3.6kg/m$^2$) between the lowest and highest quintiles of perceived neighbourhood quality should be considered in the context of an average 10,000 steps per day, where a 5% increase (500 steps) would be a worthwhile population level increase and a 5kg/m$^2$ increase in BMI is associated with a 31% increase in all-cause mortality (53). Hence, improvements in neighbourhood quality could be associated with health benefits of public health importance. There were also substantial differences in PA, BMI and fat mass % between the three housing groups studied. In particular the very low levels of PA in the social housing group during the weekend could provide a target for intervention to increase levels of PA; again these differences should be considered in relation to 500 steps per day,


For peer review only - http://bmjopen.bmj.com/site/about/guidelines.xhtml

which can be considered as an increase of population importance. Perceptions of neighbourhood quality reduced differences in PA and adiposity between housing sector groups, and the possibility of measuring more objective markers of neighbourhood quality within this study has the potential to explain more (44). The future follow-up of the ENABLE London cohort will allow us to examine whether moving to 'East Village', a neighbourhood designed for healthy active living, will have a positive impact on PA and/or adiposity levels. A major aim of the study is to identify features of the local built environment that increase levels of PA which could potentially help to reduce socioeconomic inequalities in health. It will be of particular interest to determine whether an increase in PA is more apparent in the social housing group whose neighbourhood characteristics should improve. Furthermore, we will be in a position to examine whether any potential effects of the built environment on PA are modified by housing sector type.

For peer review only


For peer review only - http://bmjopen.bmj.com/site/about/guidelines.xhtml

Ethical approval

Full ethical approval was obtained from the relevant Multi-Centre Research Ethics Committee (REC Reference 12/LO/1031). All participants provided written informed consent.

Data sharing statement

Further details of the ENABLE London study are available from the study website (http://www.enable.sgul.ac.uk/). The ongoing collection and management of data has been made possible through grant funding from the Medical Research Council and the National Institute of Health Research. We welcome proposals for collaborative projects. For general data sharing inquiries, contact Professor Owen (cowen@sgul.ac.uk).

Competing interests

We declare that we have no competing interests.

Funding

This research is being supported by project grants from the Medical Research Council National Prevention Research Initiative (MR/J000345/1) and National Institute for Health Research (12/211/69). Diabetes and obesity prevention research at St George's, University of London is supported by the National Institute for Health Research (NIHR) Collaboration for Leadership in Applied Health Research and Care (CLAHRC) South London. AE is funded by the UK Medical Research Council as part of the Neighbourhoods and Communities Programme (MC_UU_12017-10). BG-C is supported by an NHMRC Principal Research Fellowship (#1107672). ARC and ASP are supported by NIHR Biomedical Research Centre at


For peer review only - http://bmjopen.bmj.com/site/about/guidelines.xhtml

University Hospitals Bristol NHS Foundation Trust and the University of Bristol. The views expressed in this publication are those of the author(s) and not necessarily those of the NHS, the National Institute for Health Research or the Department of Health.

Authorship statement

CGO, ARR, AE, ARC, DL, SC, BG-C, DGC and PHW designed the study and raised funding. BR, ARR, CC, DP and CGO collected data for the study; BR, ARR and CGO enrolled participants. CMN, BR, ESL, ARR, CC, DP and CGO undertook data management. CMN, ESL analysed the data; CMN wrote the first draft of the report. ARR, BR, ESL, AS, DP, ARC, ASP, AE, BG-C, CC, DL, SC, PHW, DGC and CGO critically appraised the manuscript and approved the final draft. CGO is responsible for data integrity.

Acknowledgements

The authors thank the East Thames Group, Triathlon Homes and Get Living London who have assisted in recruiting participants into the ENABLE London study. The ENABLE London study is advised by a Steering Committee chaired by Professor Hazel Inskip (University of Southampton), with Dr David Ogilvie (University of Cambridge) and Professor Andy Jones (University of East Anglia) as academic advisors and Mrs Kate Worley (formerly East Thames Group Assistant Director for Strategic Housing) as the lay/stakeholder member. The authors are grateful to the members of the ENABLE London study team (in particular Aine Hogan, Katrin Peuker, Cathy McKay) and to participating households, without whom this study would not be possible.



For peer review only - http://bmjopen.bmj.com/site/about/guidelines.xhtml

Reference List


1. Ford ES, Williamson DF, Liu S. Weight change and diabetes incidence: findings from a national cohort of US adults. Am J Epidemiol 1997;146:214-22.

2. Nocon M, Hiemann T, Muller-Riemenschneider F, Thalau F, Roll S, Willich SN. Association of physical activity with all-cause and cardiovascular mortality: a systematic review and meta-analysis. Eur J Cardiovasc Prev Rehabil 2008;15:239-46.

3. Shaper AG, Wannamethee SG, Walker M. Body weight: implications for the prevention of coronary heart disease, stroke, and diabetes mellitus in a cohort study of middle aged men. BMJ 1997;314:1311-7.

4. Whitlock G, Lewington S, Sherliker P, Clarke R, Emberson J, Halsey J, Qizilbash N, Collins R, Peto R. Body-mass index and cause-specific mortality in 900 000 adults: collaborative analyses of 57 prospective studies. Lancet 2009;373:1083-96.

5. Lim SS, Vos T, Flaxman AD, Danaei G, Shibuya K, Adair-Rohani H, Amann M, Anderson HR, Andrews KG, Aryee M et al. A comparative risk assessment of burden of disease and injury attributable to 67 risk factors and risk factor clusters in 21 regions, 1990-2010: a systematic analysis for the Global Burden of Disease Study 2010. Lancet 2012;380:2224-60.

6. Health Survey for England. Health Survey for England 2012: Health, social care and lifestyles. Craig, R. and Mindell, J.  2013.  The Information Centre.

7. Chinn DJ, White M, Harland J, Drinkwater C, Raybould S. Barriers to physical activity and socioeconomic position: implications for health promotion. J Epidemiol Community Health 1999;53:191-2.

8. Beenackers MA, Kamphuis CB, Giskes K, Brug J, Kunst AE, Burdorf A, van Lenthe FJ. Socioeconomic inequalities in occupational, leisure-time, and transport related physical activity among European adults: a systematic review. Int J Behav Nutr Phys Act 2012;9:116.

9. McVeigh JA, Winkler EA, Howie EK, Tremblay MS, Smith A, Abbott RA, Eastwood PR, Healy GN, Straker LM. Objectively measured patterns of sedentary time and physical activity in young adults of the Raine study cohort. Int J Behav Nutr Phys Act 2016;13:41.

10. Sigmundova D, Sigmund E, Vokacova J, Kopkova J. Parent-child associations in pedometer-determined physical activity and sedentary behaviour on weekdays and weekends in random samples of families in the Czech Republic. Int J Environ Res Public Health 2014;11:7163-81.

11. Smith GD, Egger M. Socioeconomic differences in mortality in Britain and the United States. Am J Public Health 1992;82:1079-81.

12. Dalstra JA, Kunst AE, Mackenbach JP. A comparative appraisal of the relationship of education, income and housing tenure with less than good health among the elderly in Europe. Soc Sci Med 2006;62:2046-60.

13. Ellaway A, Macdonald L, Kearns A. Are housing tenure and car access still associated with health? A repeat cross-sectional study of UK adults over a 13-year period. BMJ Open 2016;6:e012268.




For peer review only - http://bmjopen.bmj.com/site/about/guidelines.xhtml

14. Macintyre S, Hiscock R, Kearns A, Ellaway A. Housing tenure and car access: further exploration of the nature of their relations with health in a UK setting. J Epidemiol Community Health 2001;55:330-1.

15. Macintyre S, McKay L, Der G, Hiscock R. Socio-economic position and health: what you observe depends on how you measure it. J Public Health Med 2003;25:288-94.

16. Harrison RA, McElduff P, Edwards R. Planning to win: Health and lifestyles associated with physical activity amongst 15,423 adults. Public Health 2006;120:206-12.

17. Kendig H, Browning C, Teshuva K. Health actions and social class among older Australians. Aust N Z J Public Health 1998;22:808-13.

18. Ogilvie D, Mitchell R, Mutrie N, Petticrew M, Platt S. Personal and environmental correlates of active travel and physical activity in a deprived urban population. Int J Behav Nutr Phys Act 2008;5:43.

19. Boniface DR, Cottee MJ, Neal D, Skinner A. Social and demographic factors predictive of change over seven years in CHD-related behaviours in men aged 18-49 years. Public Health 2001;115:246-52.

20. Macintyre S, Ellaway A, Hiscock R, Kearns A, Der G, McKay L. What features of the home and the area might help to explain observed relationships between housing tenure and health? Evidence from the west of Scotland. Health Place 2003;9:207-18.

21. Ellaway A, Macintyre S. Does housing tenure predict health in the UK because it exposes people to different levels of housing related hazards in the home or its surroundings? Health Place 1998;4:141-50.

22. Macintyre S, Ellaway A, Der G, Ford G, Hunt K. Do housing tenure and car access predict health because they are simply markers of income or self esteem? A Scottish study. J Epidemiol Community Health 1998;52:657-64.

23. Hoehner CM, Brennan Ramirez LK, Elliott MB, Handy SL, Brownson RC. Perceived and objective environmental measures and physical activity among urban adults. Am J Prev Med 2005;28:105-16.

24. Sallis JF, Bowles HR, Bauman A, Ainsworth BE, Bull FC, Craig CL, Sjostrom M, De B, I, Lefevre J, Matsudo V et al. Neighborhood environments and physical activity among adults in 11 countries. Am J Prev Med 2009;36:484-90.

25. Giles-Corti, B, Ryan, K, and Foster, S. Increasing density in Australia: Maximising the benefits and minimising the harm. 2012. National Heart Foundation of Australia. 16-8-2017.

26. Foster S, Giles-Corti B. The built environment, neighborhood crime and constrained physical activity: an exploration of inconsistent findings. Prev Med 2008;47:241-51.

27. da Silva IC, Payne VL, Hino AA, Varela AR, Reis RS, Ekelund U, Hallal PC. Physical Activity and Safety From Crime Among Adults: A Systematic Review. J Phys Act Health 2016;13:663-70.

28. Ram B, Nightingale CM, Hudda MT, Kapetanakis VV, Ellaway A, Cooper AR, Page A, Lewis D, Cummins S, Giles-Corti B et al. Cohort profile: Examining Neighbourhood Activities in Built



For peer review only - http://bmjopen.bmj.com/site/about/guidelines.xhtml

Living Environments in London: the ENABLE London-Olympic Park cohort. BMJ Open 2016;6:e012643.

29. Ram B, Nightingale CM, Hudda MT, Kapetanakis VV, Ellaway A, Cooper AR, Page A, Lewis D, Cummins S, Giles-Corti B et al. Cohort profile: Examining Neighbourhood Activities in Built Living Environments in London: the ENABLE London-Olympic Park cohort. BMJ Open 2016;6:e012643.

30. Ram B, Shankar A, Nightingale CM, Giles-Corti B, Ellaway A, Cooper AR, Page A, Cummins S, Lewis D, Whincup PH et al. Comparisons of depression, anxiety, well-being, and perceptions of the built environment amongst adults seeking social, intermediate and market-rent accommodation in the former London Olympic Athletes' Village. Health Place 2017;48:31-9.

31. Macintyre S, Ellaway A, Hiscock R, Kearns A, Der G, McKay L. What features of the home and the area might help to explain observed relationships between housing tenure and health? Evidence from the west of Scotland. Health Place 2003;9:207-18.

32. Mason KE, Baker E, Blakely T, Bentley RJ. Housing affordability and mental health: does the relationship differ for renters and home purchasers? Soc Sci Med 2013;94:91-7.

33. Office for National Statistics. The National Statistics Socio-economic Classification User Manual. 2005.

34. Department of Health. Start active, stay active: a report on physical activity from the four home countries Chief Medical Officers. 2011. Department of Health.

35. Freedson PS, Melanson E, Sirard J. Calibration of the Computer Science and Applications, Inc. accelerometer. Med Sci Sports Exerc 1998;30:777-81.

36. Harris T, Kerry SM, Limb ES, Victor CR, Iliffe S, Ussher M, Whincup PH, Ekelund U, Fox-Rushby J, Furness C et al. Effect of a Primary Care Walking Intervention with and without Nurse Support on Physical Activity Levels in 45- to 75-Year-Olds: The Pedometer And Consultation Evaluation (PACE-UP) Cluster Randomised Clinical Trial. PLoS Med 2017;14:e1002210.

37. Allen MS, Vella SA. Longitudinal determinants of walking, moderate, and vigorous physical activity in Australian adults. Prev Med 2015;78:101-4.

38. Juneau CE, Sullivan A, Dodgeon B, Cote S, Ploubidis GB, Potvin L. Social class across the life course and physical activity at age 34 years in the 1970 British birth cohort. Ann Epidemiol 2014;24:641-7, 647.

39. McLaren L. Socioeconomic status and obesity. Epidemiol Rev 2007;29:29-48.

40. Tranter B, Donoghue J. Housing tenure, body mass index and health in Australia. International Journal of Housing Policy 2016;1:1-20.

41. Turrell G, Haynes M, Wilson LA, Giles-Corti B. Can the built environment reduce health inequalities? A study of neighbourhood socioeconomic disadvantage and walking for transport. Health Place 2013;19:89-98.

42. Harrison RA, Gemmell I, Heller RF. The population effect of crime and neighbourhood on physical activity: an analysis of 15,461 adults. J Epidemiol Community Health 2007;61:34-9.



For peer review only - http://bmjopen.bmj.com/site/about/guidelines.xhtml

43. Brown BB, Werner CM, Smith KR, Tribby CP, Miller HJ. Physical activity mediates the relationship between perceived crime safety and obesity. Prev Med 2014;66:140-4.

44. McGinn AP, Evenson KR, Herring AH, Huston SL, Rodriguez DA. Exploring associations between physical activity and perceived and objective measures of the built environment. J Urban Health 2007;84:162-84.

45. Sarmiento O, Torres A, Jacoby E, Pratt M, Schmid TL, Stierling G. The Ciclovia-Recreativa: A mass-recreational program with public health potential. J Phys Act Health 2010;7 Suppl 2:S163-S180.

46. Hipp JA, Eyler AA, Zieff SG, Samuelson MA. Taking physical activity to the streets: the popularity of Ciclovia and Open Streets initiatives in the United States. Am J Health Promot 2014;28:S114-S115.

47. Santos-Lozano A, Santin-Medeiros F, Cardon G, Torres-Luque G, Bailon R, Bergmeir C, Ruiz JR, Lucia A, Garatachea N. Actigraph GT3X: validation and determination of physical activity intensity cut points. Int J Sports Med 2013;34:975-82.

48. Deurenberg P, Yap M, van Staveren WA. Body mass index and percent body fat: a meta analysis among different ethnic groups. Int J Obes Relat Metab Disord 1998;22:1164-71.

49. Gallagher D, Visser M, Sepulveda D, Pierson RN, Harris T, Heymsfield SB. How useful is body mass index for comparison of body fatness across age, sex, and ethnic groups? Am J Epidemiol 1996;143:228-39.

50. Hayes L, White M, Unwin N, Bhopal R, Fischbacher C, Harland J, Alberti KG. Patterns of physical activity and relationship with risk markers for cardiovascular disease and diabetes in Indian, Pakistani, Bangladeshi and European adults in a UK population. J Public Health Med 2002;24:170-8.

51. Health and Social Care Information Centre. Health Survey for England 2004: The health of ethnic minority groups. Sproston, K. and Mindell, J. 2006. The Information Centre.

52. Health and Social Care Information Centre. Health Survey for England - 2008: Physical activity and fitness. Craig R, Mindell J, and Hirani V. 2009. NHS Digital.

53. Global BMI MC, Di AE, Bhupathiraju S, Wormser D, Gao P, Kaptoge S, Berrington de GA, Cairns BJ, Huxley R, Jackson C et al. Body-mass index and all-cause mortality: individual-participant-data meta-analysis of 239 prospective studies in four continents. Lancet 2016;388:776-86.



For peer review only - http://bmjopen.bmj.com/site/about/guidelines.xhtml

For peer review only

Table 1: Associations between adiposity and physical activity outcomes and patient characteristics

| | | Difference or % difference* in adiposity/physical activity (95% CI), p-value | | | | | | | | | | | | | | |
|---|---|---|---|---|---|---|---|---|---|---|---|---|---|---|---|---|
| | n | BMI ($kg/m^2$)* | | | Fat mass % | | | Daily steps† | | | Daily minutes spent in MVPA† | | | Daily minutes spent in MVPA in ≥10 minute bouts† | | |
| **Sex** | | | | | | | | | | | | | | | | |
| Male (Ref) | 522 | | | - | | | - | | | - | | | - | | | - |
| Female | 718 | -1.2 | (-3.2, 0.9) | 0.26 | 11.1 | (10.3, 12.0) | <0.0001 | -570 | (-946, -194) | 0.003 | -9.3 | (-12.2, -6.4) | <0.0001 | -4.1 | (-6.1, -2.0) | <0.001 |
| **Age group** | | | | | | | | | | | | | | | | |
| Age 16-24 (Ref) | 269 | | | - | | | - | | | - | | | - | | | - |
| Age 25-34 | 531 | 6.3 | (3.5, 9.1) | <0.0001 | 3.2 | (2.1, 4.3) | <0.0001 | 502 | (11, 992) | 0.04 | 4.0 | (0.2, 7.9) | 0.04 | 1.0 | (-1.9, 3.8) | 0.51 |
| Age 35-49 | 358 | 13.4 | (10.2, 16.6) | <0.0001 | 6.4 | (5.2, 7.6) | <0.0001 | 699 | (173, 1224) | 0.01 | 3.9 | (-0.2, 8.0) | 0.07 | -1.1 | (-4.0, 1.8) | 0.46 |
| Age 50+ | 82 | 17.6 | (12.6, 22.9) | <0.0001 | 9.2 | (7.3, 11.0) | <0.0001 | -9 | (-832, 813) | 0.98 | -6.0 | (-12.4, 0.5) | 0.07 | -2.0 | (-6.8, 2.7) | 0.40 |
| **Ethnic group** | | | | | | | | | | | | | | | | |
| White (Ref) | 595 | | | - | | | - | | | - | | | - | | | - |
| Black | 314 | 6.2 | (3.3, 9.3) | <0.0001 | 3.6 | (2.4, 4.8) | <0.0001 | -1116 | (-1657, -575) | <0.0001 | -7.4 | (-11.7, -3.2) | <0.001 | -6.6 | (-9.8, -3.4) | <0.0001 |
| Asian | 210 | -0.3 | (-3.1, 2.7) | 0.85 | 0.02 | (-1.2, 1.3) | 0.97 | -1409 | (-1972, -845) | <0.0001 | -11.5 | (-15.9, -7.0) | <0.0001 | -8.1 | (-11.4, -4.8) | <0.0001 |
| Other/Mixed | 121 | 1.3 | (-2.3, 5.0) | 0.48 | 1.0 | (-0.5, 2.5) | 0.18 | -430 | (-1100, 239) | 0.21 | -4.6 | (-9.8, 0.7) | 0.09 | -4.0 | (-7.9, -0.04) | 0.05 |
| **Limiting illness** | | | | | | | | | | | | | | | | |
| No (Ref) | 1087 | | | - | | | - | | | - | | | - | | | - |
| Yes | 153 | 4.3 | (1.1, 7.5) | 0.01 | 1.6 | (0.3, 2.9) | 0.01 | -1081 | (-1666, -496) | <0.001 | -5.7 | (-10.3, -1.1) | 0.01 | -2.8 | (-6.1, 0.5) | 0.10 |
| **Housing sector** | | | | | | | | | | | | | | | | |
| Social | 512 | 5.0 | (2.2, 7.8) | <0.001 | 2.7 | (1.5, 3.8) | <0.0001 | -1125 | (-1629, -620) | <0.0001 | -7.5 | (-11.5, -3.6) | <0.001 | -6.5 | (-9.5, -3.5) | <0.0001 |
| Intermediate (Ref) | 503 | | | - | | | - | | | - | | | - | | | - |
| Market-rent | 225 | -0.8 | (-3.6, 2.0) | 0.57 | -0.2 | (-1.4, 1.0) | 0.70 | -104 | (-633, 424) | 0.70 | 2.3 | (-1.9, 6.4) | 0.29 | 2.8 | (-0.3, 6.0) | 0.08 |

* Percentage differences are presented for BMI, which was log-transformed for analysis
All differences and % differences are mutually adjusted for sex, age group, ethnic group, limiting longstanding illness, housing sector and a random effect to allow for clustering at household level
† Missing data for 133 participants
MVPA and MVPA in ≥10 minute bouts are an average daily estimate, obtained from averaging a participant's weekly total.



For peer review only

For peer review only - http://bmjopen.bmj.com/site/about/guidelines.xhtml

Table 2: Associations between adiposity and physical activity outcomes and neighbourhood perceptions scales

| | Difference or % difference* in outcome between the highest and lowest quintiles for each neighbourhood scale (95% CI), p-value | | | | | |
|---|---|---|---|---|---|---|
| | Perceptions of NH quality | | | Perceptions of NH crime | | |
| Adiposity (N = 1240) | | | | | | |
| Body mass index (kg/m$^2$)* | -3.6 | (-6.5, -0.6) | 0.02 | -2.1 | (-5.4, 1.3) | 0.21 |
| Fat mass % | -1.2 | (-2.5, 0.06) | 0.06 | -0.8 | (-2.2, 0.7) | 0.30 |
| Physical activity (N = 1107) | | | | | | |
| Daily steps | 677 | (108, 1247) | 0.02 | -63 | (-713, 587) | 0.85 |
| Daily MVPA (minutes) | 4.5 | (0.02, 9.0) | 0.05 | 1.1 | (-4.0, 6.2) | 0.68 |
| Daily MVPA in ≥10 minute bouts (minutes) | 2.7 | (-0.6, 6.0) | 0.11 | 2.4 | (-1.4, 6.1) | 0.22 |

* Percentage differences are presented for BMI, which was log-transformed for analysis
All differences and % differences are adjusted for sex, age group, ethnic group, limiting longstanding illness, housing sector and a random effect to allow for clustering at household level.
Abbreviations: NH, neighbourhood
MVPA and MVPA in ≥10 minute bouts are an average daily estimate, obtained from averaging a participant's weekly total.

For peer review only



For peer review only - http://bmjopen.bmj.com/site/about/guidelines.xhtml

Table 3: Adiposity and physical activity differences between housing sectors: adjustment for perceptions of neighbourhood quality

| | Housing sector group | Difference or % difference* compared to intermediate housing group (95% confidence interval), p-value | | | | | |
|---|---|---|---|---|---|---|---|
| | | Model 1 | | | Model 2 (Additionally adjusted for neighbourhood quality scale) | | |
| Adiposity (N = 1240) | | | | | | | |
| Body mass index (kg/m$^2$)* | Social | 5.0 | (2.2, 7.8) | <0.001 | 4.5 | (1.7, 7.3) | 0.002 |
| | Intermediate | Reference group | | | | | |
| | Market rent | -0.8 | (-3.6, 2.0) | 0.57 | -0.9 | (-3.6, 2.0) | 0.55 |
| Fat mass % | Social | 2.7 | (1.5, 3.8) | <0.0001 | 2.5 | (1.4, 3.6) | <0.0001 |
| | Intermediate | Reference group | | | | | |
| | Market rent | -0.2 | (-1.4, 1.0) | 0.70 | -0.2 | (-1.4, 0.9) | 0.68 |
| Physical activity (N = 1107) | | | | | | | |
| Daily steps | Social | -1125 | (-1629, -620) | <0.0001 | -1016 | (-1531, -501) | <0.001 |
| | Intermediate | Reference group | | | | | |
| | Market rent | -104 | (-633, 424) | 0.70 | -96 | (-624, 431) | 0.72 |
| Daily MVPA (minutes) | Social | -7.5 | (-11.5, -3.6) | <0.001 | -6.8 | (-10.8, -2.7) | 0.001 |
| | Intermediate | Reference group | | | | | |
| | Market rent | 2.3 | (-1.9, 6.4) | 0.29 | 2.3 | (-1.8, 6.5) | 0.27 |
| Daily MVPA in ≥10 minute bouts (minutes) | Social | -6.5 | (-9.5, -3.5) | <0.0001 | -6.0 | (-9.1, -3.0) | <0.001 |
| | Intermediate | Reference group | | | | | |
| | Market rent | 2.8 | (-0.3, 6.0) | 0.08 | 2.8 | (-0.3, 6.0) | 0.08 |

* Percentage differences are presented for BMI, which was log-transformed for analysis
Model 1: Adjusted for sex, age group, ethnic group, limiting longstanding illness and clustering at household level (random effect)
Model 2: Adjusted as Model 1 plus neighbourhood quality scale (added as a continuous variable)
MVPA and MVPA in ≥10 minute bouts are an average daily estimate, obtained from averaging a participant's weekly total.



For peer review only - http://bmjopen.bmj.com/site/about/guidelines.xhtml

Figure 1: Daily physical activity by day of the week and housing sector group: N = 6206 days from 1107 participants

Means and 95% confidence intervals are adjusted for sex, age group, ethnic group, limiting longstanding illness, month of recording, day order of recording, day of week, housing sector, an interaction between housing sector and day of week and random effects to allow for multiple days of measurement and clustering of participants within households

For peer review only



For peer review only - http://bmjopen.bmj.com/site/about/guidelines.xhtml

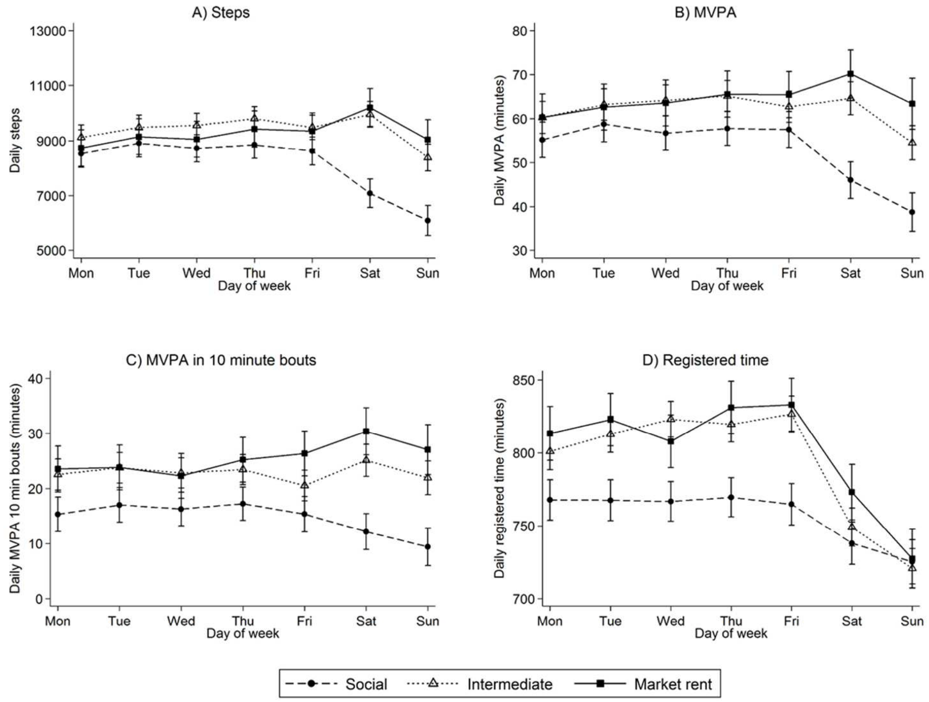


Figure 1: Daily physical activity by day of the week and housing sector group: N = 6206 days from 1107 participants

119x90mm (300 x 300 DPI)

**Supplementary Table 1: Questionnaire items included in the factor analysis on perceptions of the neighbourhood**

| |
|---|
| **Perceptions of neighbourhood crime items** |
| There is a lot of crime in my neighbourhood. |
| The level of crime in my neighbourhood makes it unsafe to walk on the streets at night. |
| There are threatening groups of young people in my neighbourhood. |
| The level of crime in my neighbourhood makes it unsafe to walk on the streets during the day. |
| Vandalism, graffiti or deliberate damage to property is a problem in my local area. |
| **Perceptions of neighbourhood quality items** |
| I enjoy walking in my neighbourhood. |
| This area is a place I enjoy living in. |
| My neighbourhood is attractive to look at (e.g. there are attractive buildings, green space. Landscaping views). |
| This area has good leisure things for people like myself, leisure centres or community centres for example. |
| You often see people out on walks or riding their bicycles in my neighbourhood. |
| This area has good local transport. |
| **Additional items included in the factor analysis with factor loadings below 0.4 and were therefore not included in the solution** |
| My neighbourhood is generally free from litter. |
| There is too much traffic in my neighbourhood. |
| Our neighbourhood streets have good lighting at night. |

Participants were asked to select a response from the following for all questionnaire items stated in the table: Strongly agree, Agree, Neither agree nor disagree, Disagree, Strongly disagree

For peer review only

**Supplementary Table 2: Participant characteristics for 1240 adults with measurements of adiposity at baseline**

| | Housing sector | | | | | | | | |
|---|---|---|---|---|---|---|---|---|---|
| | Social | | Intermediate | | Market rent | | Total | | |
| | (n = 512) | | (n = 503) | | (n = 225) | | (N = 1240) | | p ($\chi^2$) |
| **Sex** | | | | | | | | | |
| Male | 137 | (26.8%) | 259 | (51.5%) | 126 | (56.0%) | 522 | (42.1%) | |
| Female | 375 | (73.2%) | 244 | (48.5%) | 99 | (44.0%) | 718 | (57.9%) | <0.0001 |
| **Age group** | | | | | | | | | |
| 16-24 | 107 | (20.9%) | 92 | (18.3%) | 70 | (31.1%) | 269 | (21.7%) | |
| 25-34 | 129 | (25.2%) | 291 | (57.9%) | 111 | (49.3%) | 531 | (42.8%) | |
| 35-49 | 233 | (45.5%) | 102 | (20.3%) | 23 | (10.2%) | 358 | (28.9%) | |
| 50+ | 43 | (8.4%) | 18 | (3.6%) | 21 | (9.3%) | 82 | (6.6%) | <0.0001 |
| **Ethnic group** | | | | | | | | | |
| White | 96 | (18.8%) | 342 | (68.0%) | 157 | (69.8%) | 595 | (48.0%) | |
| Black | 245 | (47.9%) | 53 | (10.5%) | 16 | (7.1%) | 314 | (25.3%) | |
| Asian | 107 | (20.9%) | 75 | (14.9%) | 28 | (12.4%) | 210 | (16.9%) | |
| Mixed/Other | 64 | (12.5%) | 33 | (6.6%) | 24 | (10.7%) | 121 | (9.8%) | <0.0001 |
| **NS-SEC*** | | | | | | | | | |
| Higher Managerial / Professional | 60 | (11.9%) | 357 | (71.4%) | 150 | (66.7%) | 567 | (46.1%) | |
| Intermediate Occupations | 62 | (12.3%) | 77 | (15.4%) | 38 | (16.9%) | 177 | (14.4%) | |
| Routine / Manual | 125 | (24.8%) | 34 | (6.8%) | 10 | (4.4%) | 169 | (13.7%) | |
| Economically inactive | 258 | (51.1%) | 32 | (6.4%) | 27 | (12.0%) | 317 | (25.8%) | <0.0001 |
| **Limiting illness** | | | | | | | | | |
| Yes | 102 | (19.9%) | 40 | (8.0%) | 11 | (4.9%) | 153 | (12.3%) | |
| No | 410 | (80.1%) | 463 | (92.0%) | 214 | (95.1%) | 1087 | (87.7%) | <0.0001 |

p ($\chi^2$): p-value for Chi-squared test
* 10 responses missing for NS-SEC group

For peer review only

**Supplementary Table 3: Mean levels of adiposity and physical activity by patient characteristics**

| | | Mean/Geometric mean* levels adiposity and physical activity (95% confidence intervals) | | | | | | | | | |
|---|---|---|---|---|---|---|---|---|---|---|---|
| | n | BMI (kg/m$^2$)* | | Fat mass % | | Daily steps† | | Daily minutes of MVPA† | | Daily minutes of MVPA in ≥10 minute bouts† | |
| **Sex** | | | | | | | | | | | |
| Male | 522 | 25.4 | (25.0, 25.8) | 20.4 | (19.7, 21.0) | 9279 | (8991, 9568) | 64.8 | (62.6, 67.1) | 22.8 | (21.1, 24.4) |
| Female | 718 | 25.1 | (24.8, 25.5) | 31.5 | (30.9, 32.0) | 8709 | (8464, 8954) | 55.6 | (53.6, 57.5) | 18.7 | (17.3, 20.1) |
| **Age group** | | | | | | | | | | | |
| Age 16-24 | 269 | 23.5 | (23.0, 24.0) | 23.0 | (22.1, 23.9) | 8534 | (8136, 8932) | 57.0 | (53.9, 60.2) | 20.5 | (18.2, 22.8) |
| Age 25-34 | 531 | 25.0 | (24.6, 25.3) | 26.2 | (25.5, 26.8) | 9035 | (8744, 9326) | 61.1 | (58.8, 63.3) | 21.4 | (19.7, 23.1) |
| Age 35-49 | 358 | 26.6 | (26.1, 27.1) | 29.3 | (28.5, 30.1) | 9232 | (8879, 9585) | 60.9 | (58.1, 63.6) | 19.4 | (17.3, 21.4) |
| Age 50+ | 82 | 27.6 | (26.6, 28.7) | 32.2 | (30.5, 33.8) | 8525 | (7800, 9249) | 51.1 | (45.4, 56.7) | 18.4 | (14.2, 22.7) |
| **Ethnic group** | | | | | | | | | | | |
| White | 595 | 24.9 | (24.5, 25.2) | 25.8 | (25.1, 26.4) | 9491 | (9203, 9779) | 63.6 | (61.3, 65.8) | 23.7 | (22.0, 25.4) |
| Black | 314 | 26.4 | (25.8, 27.0) | 29.4 | (28.5, 30.3) | 8375 | (7961, 8789) | 56.2 | (52.9, 59.4) | 17.1 | (14.7, 19.6) |
| Asian | 210 | 24.8 | (24.2, 25.4) | 25.8 | (24.8, 26.8) | 8082 | (7608, 8556) | 52.1 | (48.4, 55.8) | 15.6 | (12.8, 18.4) |
| Other/Mixed | 121 | 25.2 | (24.4, 26.0) | 26.8 | (25.5, 28.1) | 9060 | (8465, 9656) | 59.0 | (54.3, 63.7) | 19.8 | (16.3, 23.3) |
| **Limiting illness** | | | | | | | | | | | |
| No | 1087 | 25.1 | (24.9, 25.4) | 26.6 | (26.1, 27.0) | 9077 | (8877, 9277) | 60.1 | (58.6, 61.7) | 20.8 | (19.6, 21.9) |
| Yes | 153 | 26.2 | (25.5, 27.0) | 28.2 | (27.0, 29.4) | 7996 | (7447, 8545) | 54.4 | (50.1, 58.7) | 18.0 | (14.8, 21.1) |
| **Housing sector** | | | | | | | | | | | |
| Social | 512 | 26.0 | (25.6, 26.5) | 28.4 | (27.6, 29.1) | 8298 | (7953, 8642) | 54.6 | (51.8, 57.3) | 16.0 | (14.0, 18.1) |
| Intermediate | 503 | 24.8 | (24.4, 25.2) | 25.7 | (25.0, 26.4) | 9422 | (9110, 9735) | 62.1 | (59.6, 64.5) | 22.5 | (20.6, 24.4) |
| Market-rent | 225 | 24.6 | (24.0, 25.2) | 25.5 | (24.5, 26.5) | 9318 | (8863, 9773) | 64.3 | (60.8, 67.9) | 25.3 | (22.6, 28.1) |

* Geometric means are presented for BMI
All means/geometric means are adjusted for sex, age group, ethnic group, limiting longstanding illness, housing sector and a random effect to allow for clustering at household level.
† Data missing for 133 participants for average daily steps, MVPA and MVPA in bouts

For peer review only

**Supplementary Table 4: Associations between participant characteristics and physical activity variables in participants with at least 4 days of recording of physical activity data**

| | n | Difference or % difference* in physical activity variable (95% confidence interval), p-value | | | | | | | | |
|---|---|---|---|---|---|---|---|---|---|---|
| | | Daily steps | | | Daily minutes spent in MVPA | | | Daily minutes spent in MVPA in ≥10 minute bouts | | |
| **Sex** | | | | | | | | | | |
| Male (Ref) | 402 | | - | | | - | | | - | |
| Female | 529 | -559 | (-947, -172) | 0.005 | -9.7 | (-12.7, -6.7) | <0.0001 | -4.7 | (-6.8, -2.5) | <0.0001 |
| **Age group** | | | | | | | | | | |
| Age 16-24 (Ref) | 180 | | - | | | - | | | - | |
| Age 25-34 | 412 | 410 | (-116, 936) | 0.13 | 3.0 | (-1.2, 7.2) | 0.16 | -0.3 | (-3.3, 2.8) | 0.87 |
| Age 35-49 | 276 | 520 | (-45, 1086) | 0.07 | 2.5 | (-2.0, 6.9) | 0.27 | -2.5 | (-5.7, 0.7) | 0.12 |
| Age 50+ | 63 | -25 | (-889, 838) | 0.95 | -7.9 | (-14.8, -1.1) | 0.02 | -3.2 | (-8.2, 1.8) | 0.20 |
| **Ethnic group** | | | | | | | | | | |
| White (Ref) | 482 | | - | | | - | | | - | |
| Black | 214 | -1213 | (-1789, -638) | <0.0001 | -7.1 | (-11.7, -2.5) | 0.002 | -6.5 | (-9.9, -3.1) | <0.001 |
| Asian | 142 | -1128 | (-1719, -538) | <0.001 | -10.3 | (-15.0, -5.6) | <0.0001 | -7.8 | (-11.3, -4.3) | <0.0001 |
| Other/Mixed | 93 | -582 | (-1273, 110) | 0.10 | -4.7 | (-10.2, 0.8) | 0.09 | -4.2 | (-8.2, -0.2) | 0.04 |
| **Limiting illness** | | | | | | | | | | |
| No (Ref) | 834 | | - | | | - | | | - | |
| Yes | 97 | -976 | (-1612, -341) | 0.003 | -4.6 | (-9.6, 0.4) | 0.07 | -2.0 | (-5.6, 1.6) | 0.28 |
| **Housing sector** | | | | | | | | | | |
| Social | 332 | -978 | (-1515, -440) | <0.001 | -6.9 | (-11.2, -2.6) | 0.002 | -7.2 | (-10.4, -4.0) | <0.0001 |
| Intermediate (Ref) | 410 | | - | | | - | | | - | |
| Market-rent | 189 | -359 | (-889, 171) | 0.185 | 0.2 | (-4.0, 4.3) | 0.91 | 0.9 | (-2.2, 4.1) | 0.56 |

All differences are mutually adjusted for sex, age group, ethnic group, limiting longstanding illness, housing sector and a random effect to allow for clustering at household level.
MVPA and MVPA in ≥10 minute bouts are an average daily estimate, obtained from averaging a participant's weekly total.

For peer review only

**Supplementary Table 5: Physical activity differences between weekday (Monday-Friday) and weekend (Saturday, Sunday) activity by housing sector.**

| Physical activity variable (N = 1107) | Housing sector group | Mean (95% CI) weekday (Mon-Fri) activity | | Difference in PA outcome compared to weekdays (95% confidence interval), p-value | | | | | |
|---|---|---|---|---|---|---|---|---|---|
| | | | | Saturday - weekday | | | Sunday - weekday | | |
| Daily steps | Social | 8733 | (8364, 9103) | -1643 | (-2078, -1207) | <0.0001 | -2629 | (-3093, -2164) | <0.0001 |
| | Intermediate | 9497 | (9178, 9817) | 460 | (59, 862) | 0.02 | -1104 | (-1528, -680) | <0.0001 |
| | Market-rent | 9146 | (8673, 9619) | 1055 | (467, 1642) | <0.001 | -102 | (-734, 531) | 0.75 |
| MVPA (minutes) | Social | 57.2 | (54.3, 60.1) | -11.2 | (-14.7, -7.7) | <0.0001 | -18.4 | (-22.1, -14.7) | <0.0001 |
| | Intermediate | 63.1 | (60.6, 65.7) | 1.5 | (-1.8, 4.7) | 0.37 | -8.5 | (-11.9, -5.1) | <0.0001 |
| | Market-rent | 63.5 | (59.8, 67.3) | 6.6 | (1.9, 11.3) | 0.01 | -0.1 | (-5.2, 5.0) | 0.97 |
| MVPA in ≥10 minute bouts (minutes) | Social | 16.3 | (14.0, 18.5) | -4.1 | (-6.9, -1.3) | 0.004 | -6.8 | (-9.8, -3.9) | <0.0001 |
| | Intermediate | 22.6 | (20.7, 24.6) | 2.5 | (-0.06, 5.1) | 0.06 | -0.7 | (-3.4, 2.0) | 0.62 |
| | Market-rent | 24.2 | (21.3, 27.1) | 6.1 | (2.4, 9.9) | 0.001 | 2.8 | (-1.2, 6.9) | 0.17 |

Means and differences (95% confidence intervals) are adjusted for sex, age group, ethnic group, limiting longstanding illness, month of recording, day of the week, housing sector, an interaction between housing sector and day of week, and random effects to allow for multiple days of measurement and clustering of participants within household.

MVPA and MVPA in ≥10 minute bouts are an average daily estimate, obtained from averaging a participant's weekly total.

For peer review only

# Housing, neighbourhood and sociodemographic associations with adult levels of physical activity and adiposity: baseline findings from the ENABLE London Study

| | |
|---|---|
| Journal: | *BMJ Open* |
| Manuscript ID | bmjopen-2017-021257.R2 |
| Article Type: | Research |
| Date Submitted by the Author: | 11-Jul-2018 |
| Complete List of Authors: | Nightingale, C; St George's University London, Division of Population Health Sciences and Education<br>Rudnicka, Alicja; SGUL University of London<br>Ram, Bina; St George's, University of London, Population Health Research Institute<br>Shankar, Aparna; St. Geoege's, University of London, Population Health Research Institute<br>Limb, Elizabeth; St George's University, Population Health Research Institute<br>Procter, Duncan; University of Bristol, Centre for Exercise, Nutrition and Health Sciences<br>Cooper, Ashley; University of Bristol,<br>Page, Angie; University of Bristol, Centre for Exercise, Nutrition and Health Sciences<br>Ellaway, Anne; University of Glasgow, MRC/SCO Social and Public Health Sciences Unit<br>Giles-Corti, Billie; RMIT University, NHMRC Centre of Research Excellence in Healthy Liveable Communities<br>Clary, Christelle; London School of Hygiene and Tropical Medicine, Social And Environmental Health Research<br>Lewis, Daniel; London School of Hygiene and Tropical Medicine,<br>Cummins, Steven; London School of Hygiene and Tropical Medicine, Department of Social and Environmental Health Research<br>Whincup, Peter; St Georges, University of London<br>Cook, Derek; St George's University, Division of Population Health Sciences<br>Owen, C; St George's, University London, Population Health Research Institute |
| <b>Primary Subject Heading</b>: | Epidemiology |
| Secondary Subject Heading: | Cardiovascular medicine |
| Keywords: | Physical activity, Adiposity, Housing, Perceived neighbourhood environment, ENABLE-London |
| | |

For peer review only

# Housing, neighbourhood and sociodemographic associations with adult levels of physical activity and adiposity: baseline findings from the ENABLE London Study

CM Nightingale[1], AR Rudnicka[1], B Ram[1], A Shankar[1], ES Limb[1], D Procter[2,3], AR Cooper[2, 3], AS Page[2, 3], A Ellaway[4], B Giles-Corti[5], C Clary[6], D Lewis[6], S Cummins[6], PH Whincup[1], DG Cook[1], CG Owen[1]

[1] Population Health Research Institute, St George's, University of London, London, UK.

[2] Centre for Exercise, Nutrition and Health Sciences, University of Bristol, Bristol, UK.

[3] National Institute for Health Research Bristol Biomedical Research Centre, University Hospitals Bristol NHS Foundation Trust and University of Bristol, UK.

[4] MRC/SCO Social and Public Health Sciences Unit, University of Glasgow, Glasgow, UK.

[5] NHMRC Centre of Research Excellence in Healthy Liveable Communities, RMIT University, Melbourne, Australia.

[6] London School of Hygiene and Tropical Medicine, London, UK.

Corresponding Author:-

Dr Claire M Nightingale

Population Health Research Institute, St. George's, University of London, Cranmer Terrace, London SW17 0RE. Email: cnightin@sgul.ac.uk. Phone: +44 (0) 208 725 2342

**Total word count:** 4550 excluding abstract (311 words)

**Tables and figures:** 3 tables, 1 figure, 5 supplementary tables

**References:** 51



For peer review only - http://bmjopen.bmj.com/site/about/guidelines.xhtml

## *Abstract*

**Objectives:** The neighbourhood environment is increasingly shown to be an important correlate of health. We assessed associations between housing tenure, neighbourhood perceptions, sociodemographic factors, and levels of physical activity (PA) and adiposity among adults seeking housing in East Village (formerly London 2012 Olympic/Paralympic Games Athletes' Village).

**Setting:** Cross-sectional analysis of adults seeking social, intermediate and market-rent housing in East Village.

**Participants:** 1278 participants took part in the study (58% female). Complete data on adiposity (body mass index [BMI] and fat mass %) were available for 1240 participants (97%); of these a sub-set of 1107 participants (89%) met the inclusion criteria for analyses of accelerometer-based measurements of PA. We examined associations between housing sector sought, neighbourhood perceptions (covariates) and PA and adiposity (dependent variables) adjusted for household clustering, sex, age group, ethnic group, and limiting longstanding illness.

**Results:** Participants seeking social housing had the fewest daily steps (8304, 95%CI 7959,8648) and highest BMI (26.0kg/m$^2$ 95%CI 25.5,26.5kg/m$^2$) compared with those seeking intermediate (daily steps 9417, 95%CI 9106,9731; BMI 24.8kg/m$^2$ 95%CI 24.4,25.2kg/m$^2$) or market-rent housing (daily steps 9313, 95%CI 8858,9768; BMI 24.6kg/m$^2$ 95%CI 24.0,25.2kg/m$^2$). Those seeking social housing had lower levels of PA (by 19-42%) at weekends vs weekdays, compared with other housing groups. Positive perceptions of neighbourhood quality were associated with higher steps and lower BMI, with differences between social and intermediate groups reduced by ~10% following adjustment, equivalent to a reduction of 111 for steps and 0.5kg/m$^2$ for BMI.




For peer review only - http://bmjopen.bmj.com/site/about/guidelines.xhtml

**Conclusions:** The social housing group undertook less PA than other housing sectors, with weekend PA offering the greatest scope for increasing PA, and tackling adiposity in this group. Perceptions of neighbourhood quality were associated with PA and adiposity and reduced differences in steps and BMI between housing sectors. Interventions to encourage physical activity at weekends and improve neighbourhood quality, especially amongst the most disadvantaged, may provide scope to reduce inequalities in health behaviour.

**Strengths and limitations of this study**

- Large sample with representation of three different aspirational housing groups, providing a wide range of socioeconomic backgrounds
- Objective measurements of physical activity and adiposity outcomes using accelerometry and bioelectrical impedance respectively
- Lower number of participants studied seeking market-rent housing compared with those seeking intermediate or social housing

**Keywords**

Physical activity; Adiposity; Housing; Perceived neighbourhood environment; ENABLE-London


For peer review only - http://bmjopen.bmj.com/site/about/guidelines.xhtml

## *Introduction*

Physical inactivity and adiposity are associated with an increased risk of type 2 diabetes and cardiovascular disease (1-4) and constitute a serious public health problem in the UK and globally (5). Evidence suggests that levels of physical activity (PA) are lower among those who are socioeconomically disadvantaged (6), who experience greater economic, access and health related barriers to being physically active (7). Socioeconomic status is also associated with differences in types of PA, in particular higher socioeconomic status is associated with more vigorous leisure time PA (8). Previous research has found variation in PA by day of the week with studies showing lower levels of activity on Sundays compared with weekdays in young adults (9), parents and their children (10).

There is emerging evidence suggesting that housing tenure is an important determinant of health. In particular, UK-based studies have shown that housing tenure (owner vs. private renter vs. public sector renter) is associated with poor health (11;12). Amongst particular groups including those who are economically inactive or unemployed, housing tenure might provide a better indication of socioeconomic status compared with measures based on occupation or income (13). Indeed, in several studies housing tenure remained associated with health outcomes following adjustment for conventional measures of socioeconomic status such as income or education (11;14). A more nuanced approach is therefore required with respect to measures of socioeconomic status, and they should not be simply regarded as interchangeable (12;15). Despite this, there has been limited research examining the direct effect of housing tenure on PA, and existing evidence is equivocal. Harrison and colleagues found no association between housing tenure and meeting recommended levels of PA among community dwelling healthy adults in the North-East of England (16). Similarly


For peer review only - http://bmjopen.bmj.com/site/about/guidelines.xhtml

housing tenure was not associated with self-reported energetic PA among older Australians (17). Ogilvie and colleagues found overall levels of PA to be higher among individuals living in social housing compared with owner-occupiers (18). The authors suggest that may capture occupational PA levels which are likely to be higher among those in social housing (18). In contrast, living in private rental accommodation was associated with a greater likelihood of taking up exercise over a 9-year period among men aged 18-49 at baseline, compared with those in local authority accommodation (19).

Housing tenure may affect health and health behaviours in part through characteristics of the home or neighbourhood itself (20;21) or psychological factors such as self-efficacy or self-esteem (22). Social housing estates which are common in the UK may be associated with specific cultures and norms, which in turn shape residents' behaviours (11). Subjective characteristics of the neighbourhood environment including higher perceived access to recreational facilities and shops in local proximity have been shown to be associated with higher levels of PA (23;24). Residents who perceive their neighbourhood more positively, have been shown to have better mental health and are less likely to relocate (25). Conversely, real and perceived crime, has the potential to constrain residents' PA (26). However, a recent systematic review suggested a lack of association between PA and perceptions of safety from crime; highlighting the need for high quality evidence, including prospective studies and natural experiments (27), to examine this issue further. In particular, high quality evidence is needed to understand the potentially multifactorial influence of residential location on health and health behaviours; effects which are likely to extend beyond simple measures of socioeconomic status (27).


For peer review only - http://bmjopen.bmj.com/site/about/guidelines.xhtml

The Examining Neighbourhood Activities and Built Living Environments in London (ENABLE London) study is a longitudinal study evaluating how active urban design influences the health and wellbeing of people moving into the former Athletes' Village of the London 2012 Olympic and Paralympic Games now known as 'East Village' (28). East Village is a new high-density neighbourhood development built on active design principles containing a mix of social housing, intermediate (including affordable rent, shared ownership and shared equity) housing, and market-rent housing. This paper draws on baseline data (prior to any potential move to East Village) to first, examine predictors of PA and adiposity (measured objectively using accelerometry and bioelectrical impedance), including the housing sector to which they are applying and perceptions of their neighbourhood. Second, to examine whether PA patterns across the week vary by housing sector and third, to examine whether adjustment for perceptions of the neighbourhood environment reduce housing sector differences in PA and adiposity.

## *Methods*

Study participants were recruited from those seeking or who had applied for new accommodation in East Village and were classified by the type of housing tenure sought based on level of income; i.e. social, intermediate or market-rent. The inclusion criteria was broad and included anyone interested / applying for single or multiple occupancy accommodation in East Village. There was no explicit exclusion criteria; adults of any age, gender, ethnic group, with or without handicap, were invited to participate. Current housing status was strongly linked to aspirational housing status, where those seeking social accommodation were currently in social housing or on social housing waiting lists, and those seeking intermediate and market-rent accommodation were largely in privately rented


For peer review only - http://bmjopen.bmj.com/site/about/guidelines.xhtml

housing. Recruitment of participants in the different housing sectors was carried out between January 2013 and December 2015 in three phases determined by the order of availability of housing in East Village (social, intermediate, and market-rent respectively). Those applying for social housing in East Village were initially recruited between January 2013 and May 2014, households seeking intermediate accommodation between July 2013 and November 2014 and those seeking market rent accommodation between September 2014 and December 2015. Recruitment processes for those applying for social housing were slightly different compared with other housing sectors. The East Thames Group housing association was primarily responsible for recruiting participants in social housing, whereas the ENABLE London team (in association with Triathlon Homes and Get Living London) recruited participants from the other housing sectors (28). Aspirational housing tenure is integral to the design of ENABLE London, and we have shown that this provides a clear socioeconomic marker of study participants. For example, those seeking social housing in East Village are more likely to be unemployed, less educated and more likely to represent ethnic minorities (a classic marker of socioeconomic vulnerability), compared to those seeking affordable and market-rent accommodation (28). We have also shown key differences in mental health and well-being between housing groups, where those seeking social housing were more likely to be depressed, anxious and have poorer well-being, compared to other housing groups (29). Moreover, this is entirely consistent with earlier studies which found that both current housing tenure and aspirational housing tenure are associated with a variety of health outcomes, including mental health and measures of general health (20;30).


For peer review only - http://bmjopen.bmj.com/site/about/guidelines.xhtml

Baseline assessments of participants were carried out in their place of residence before any potential move to East Village. Full details of the recruitment process can be found elsewhere (28).

**Independent variables**

A team of trained fieldworkers administered self-complete questionnaires on a laptop during home visits. Data on age, sex, self-defined ethnicity, work status, occupation and whether the participant had a limiting longstanding illness or disability (lasting or expected to last at least 12 months) were collected. Participants self-defined as 'White', 'Asian', 'Black', 'Mixed', or 'Other'; the latter two categories were combined for analyses. Socioeconomic status based on occupation was coded using the National Statistics Social-Economic Coding (NS-SEC) to categorise participants into 'higher managerial or professional occupations', 'intermediate occupations', 'routine or manual' (31). An additional 'economically inactive' category included those seeking employment, unable to work due to disability or illness, retired, looking after home and family, and students. We sought information on educational attainment; participants were categorised into "Degree or equivalent / Higher", "Intermediate qualifications" (including A levels and GSCEs), and "Other / None" (including work-based or foreign qualifications). Participants completed questionnaires assessing neighbourhood perceptions (29). Five items assessed perceived crime (e.g., "There is a lot of crime in my neighbourhood"; Cronbach's $\alpha = 0.87$) and six items assessed neighbourhood quality (e.g. "This area is a place I enjoy living in"; Cronbach's $\alpha = 0.78$). Responses on items were summed and scores ranged from -10 to +10 for perceived crime and -12 to +12 for perceived quality, such that positive scores indicate less perceived crime and better neighbourhood quality while negative scores indicate more


For peer review only - http://bmjopen.bmj.com/site/about/guidelines.xhtml

perceived crime and poorer quality. The scales were derived following an exploratory factor analysis of 14 questions regarding neighbourhood (Supplementary Table 1).

**Dependent variables**

Height was measured to the last complete millimetre using a portable stadiometer; weight was measured to the nearest kilogram using a Tanita SC-240 Body Composition Analyzer (Tanita, Tokyo, Japan); body mass index (BMI) was derived as weight(kg)/height(m)$^2$. The Tanita SC-240 Body Composition Analyzer also measured leg-to-leg bioelectrical impedance from which fat free mass and fat mass were estimated. Fat mass percentage was calculated as fat mass (kg)/weight (kg)*100.

Participants wore a hip-mounted ActiGraph GT3X+ accelerometer during waking hours over a consecutive period of 7 days (ActiGraph LLC, Florida, USA). These accelerometers provided daily measures of steps, counts and time spent in moderate and vigorous PA (MVPA) using established cut-offs. Daily time spent in MVPA both overall and in ≥10 minute bouts in accordance with UK recommendations for PA (32) were assessed. The cut-point for moderate PA was defined as ≥1952 counts per minute (33). We excluded any days of recording where the amount of registered time accumulated was below 540 minutes (34). Non-wear periods were defined as a minimum length of 60 minutes, allowing for a 2-minute spike tolerance. Participants with at least one day of recording were retained in analyses. We fitted a multilevel linear model for each outcome to allow for repeated measurements of daily PA, by fitting participant as a random effect and adjusting for day of the week, day order of recording and month as fixed effects. Raw level one residuals were obtained from the model and a within person average value of each outcome variable was obtained by


For peer review only - http://bmjopen.bmj.com/site/about/guidelines.xhtml

averaging these raw residuals. The average of these raw residuals for each participant was added to the sample mean for that particular PA variable to derive an unbiased average level of each PA variable for each person.

**Statistical analysis**

All analyses were carried out using STATA/SE software (Stata/SE 14 for Windows; StataCorp LP, College Station, TX, USA). Outcome variables were inspected for normality and BMI was log transformed due to its skewed distribution. Multilevel linear regression models were fitted, mutually adjusted for housing sector and participant characteristics (sex, age group, ethnic group, and limiting longstanding illness) as fixed effects, with a random effect to allow for household clustering. Residuals did not show departure from linearity, suggesting that the model assumptions were appropriate. Absolute differences or percentage differences for log transformed outcomes (i.e. BMI) are presented by sex, age group, ethnic group, limiting longstanding illness and housing sector. Sensitivity analyses examined whether associations remained when the sample was restricted to 931 participants (84%) with at least four days of 540 or more minutes per day of recording.

To assess differences in PA by day of the week as opposed to overall levels of PA we took the following approach. Daily PA data were examined using multilevel models with random effects to allow for multiple days of recording within person and household clustering. An interaction between housing sector and day of the week was fitted and models were adjusted for sex, age group, ethnic group, limiting longstanding illness, day order of recording and month of measurement as fixed effects.


For peer review only - http://bmjopen.bmj.com/site/about/guidelines.xhtml

The associations between neighbourhood perception scales and adiposity and PA outcomes were examined. Each of the neighbourhood quality and crime scores were included in the models as quintiles, to examine the differences in outcomes between the top and bottom quintile. Finally, the effect of adjustment for neighbourhood perception on differences in adiposity and PA between housing sectors was examined. If associations between outcomes and neighbourhood perceptions appeared linear, models examining housing sector differences were additionally adjusted for neighbourhood perceptions as a continuous variable.

**Patient and Public Involvement**

The ENABLE London study was developed in partnership with a network of both local and regional stakeholders identified through our collaborator links to agencies, involved with the design, planning and management of large-scale accommodation developments. Locally these included local authorities (particularly Newham) and a number of housing associations, in particular Triathlon Homes, a partner organisation of housing associations, which manages social and intermediate homes in East Village. Participants have been involved in the study from an early stage to ensure assessments and participation remain relevant and enjoyable, to ensure the continued significance and potential generalisability of the work.

## *Results*

Of 1819 households who agreed to be contacted by the study team in order to receive further information about the ENABLE London study, 1278 adults from 1006 households (55%) participated in the study and completed a questionnaire. Participation rates for those


For peer review only - http://bmjopen.bmj.com/site/about/guidelines.xhtml

seeking market-rent and intermediate housing were 58% and 57% respectively and were slightly lower in the social group (52%). Complete data on adiposity were available for 1240 participants (97%); of these a sub-set of 1107 participants (89%) met the inclusion criteria for analyses of objectively measured PA. Participant characteristics (age, sex) and levels of adiposity were similar among those who did and did not provide PA data; however, participants from black and Asian ethnic groups were less likely to provide PA data. Supplementary Table 2 shows participants characteristics at baseline for the 1240 adults with measurements of adiposity at baseline. Those seeking social housing were more likely to be female, of older age, of non-white ethnicity, to have limiting longstanding illness, and be in routine / manual occupations or economically inactive compared to those seeking intermediate or market-rent housing.

Adjusted mean levels of adiposity and PA outcomes by housing sector and participant characteristics are shown in Supplementary Table 3. Table 1 shows housing sector and other participant characteristics associations with BMI and fat mass %, and objectively measured PA (steps, time spent in MVPA, time spent in MVPA in ≥10 minute bouts). Participants seeking social housing had markedly higher levels of BMI and fat mass % and markedly lower levels of steps, MVPA and MVPA in ≥10 minute bouts compared with those seeking intermediate housing, though there were no differences between those seeking market-rent and intermediate accommodation.

Fat mass % was higher in females than males though there was no difference in BMI (Table 1). BMI and fat mass % were higher among all older age groups compared with 16-24 year olds. Participants of black ethnicity had higher levels of BMI and fat mass % compared with


For peer review only - http://bmjopen.bmj.com/site/about/guidelines.xhtml

whites; there were no differences in BMI and fat mass % between Asian or other/mixed ethnic groups and whites. Those with a limiting longstanding illness had higher levels of both BMI and fat mass %. All PA measures were lower among females. Steps and MVPA were slightly higher in 25-34 year olds and steps were also higher among 35-49 year olds compared with 16-24 year olds; however, there were no age group differences for MVPA in ≥10 minute bouts. Participants of black and Asian ethnicities had lower levels of steps, MVPA and MVPA in ≥10 minute bouts compared to whites. Participants who reported having a limiting longstanding illness had lower levels of steps and MVPA, but not MVPA in ≥10 minute bouts. Educational attainment level was not associated with any of the outcomes once housing sector had been adjusted for and adjustment for educational attainment did not materially alter housing sector differences in adiposity or PA outcomes (data available from authors).

Sensitivity analyses for PA outcomes were carried out in 931 participants who wore an ActiGraph for at least four days with at least 540 minutes of recording per day (Supplementary Table 4). There were no differences between market-rent and intermediate groups (consistent with the main analysis presented in Table 1). Differences between social and intermediate groups were broadly similar with the results presented in Table 1 for the main analysis.

Differences in PA variables between housing groups were examined by day of the week to explore whether differences between groups were consistent across the week (Figure 1A-D). Levels of PA (steps (panel A), MVPA (panel B) and MVPA in ≥10 minute bouts (panel C)) were generally consistent across weekdays (Monday – Friday) among all groups. In the



For peer review only - http://bmjopen.bmj.com/site/about/guidelines.xhtml

intermediate group, steps were higher on Saturdays and lower on Sundays; MVPA and MVPA in ≥10 minute bouts were lower on Sundays but there was no difference on Saturdays compared to weekday activity. In the market-rent group, steps, MVPA and MVPA in ≥10 minute bouts were higher on Saturdays and similar to weekdays on Sundays. In the social group, steps, MVPA and MVPA in ≥10 minute bouts were on average lower on Saturdays and lower still on Sundays. Registered time (panel D) was lowest on average in the social group during weekdays, decreasing on Saturdays and Sundays. The intermediate and market-rent groups had higher levels of registered time during weekdays compared with the social group which decreased on average on Saturdays and Sundays (despite recording more steps and minutes in MVPA suggesting a higher intensity of activity). Mean levels of steps, MVPA, and MVPA in ≥10 minute bouts on weekdays and differences on Saturday and Sunday compared to weekdays are shown by housing sector in Supplementary Table 5. The marked differences in activity between weekdays and weekend days in the social group are not explained by differences in registered time (data available from authors).

Associations between perceived neighbourhood quality and crime scales and adiposity and PA outcomes are shown in Table 2, adjusted for the participant characteristics shown in Table 1. Participants with the most positive perceptions of neighbourhood quality (highest quintile) had lower BMI, higher steps and recorded longer durations of MVPA compared with those who had the most negative perceptions of neighbourhood quality (lowest quintile). There were no significant associations between perceptions of neighbourhood crime and adiposity or PA.



For peer review only - http://bmjopen.bmj.com/site/about/guidelines.xhtml

The effect of adjustment for perceived neighbourhood quality on differences in adiposity and PA between housing sector groups is presented in Table 3. All associations between perceived neighbourhood quality and crime and outcome variables were approximately linear and were therefore fitted as continuous variables in the model. In addition, associations between perceived neighbourhood quality and crime and outcome variables were similar across the three housing groups (all $p>0.05$). Adjustment for perceptions of neighbourhood quality reduced differences in BMI, fat mass %, steps, MVPA and MVPA in ≥10 minute bouts between the social and intermediate groups by 10%, 6%, 10%, 10% and 7% respectively. Differences between market-rent and intermediate groups in adiposity and PA variables were not statistically significant before or after adjustment. A larger proportion of the social-intermediate group differences in steps, MVPA and MVPA in ≥10 minute bouts on weekends was explained by adjustment for perceptions of neighbourhood quality (10%, 16% and 16% respectively) compared to the differences in steps, MVPA and MVPA in ≥10 minute bouts on weekdays which were reduced by 10%, 8% and 3% respectively (data not shown).

## *Discussion*

The results of this study showed that participants seeking social housing in East Village had lower levels of PA and higher levels of BMI and fat mass % compared with those seeking intermediate and market-rent housing, even when adjusted for demographic factors. In the social housing group, levels of PA were particularly low on weekends compared with weekdays possibly reflecting higher occupational PA and lower leisure time PA; weekday-weekend differences in PA were less marked among those seeking intermediate and market-rent housing. However, the lower registered time at weekends but higher MVPA


For peer review only - http://bmjopen.bmj.com/site/about/guidelines.xhtml

and steps suggests more intense activity at weekends in the intermediate and market-rent housing groups. These findings may inform targeted interventions to increase PA and reduce adiposity in different socioeconomic groups.

Positive associations between perceived neighbourhood quality and PA, BMI and fat mass % were also shown. Adjustment for differences in perceived neighbourhood quality reduced differences in PA and BMI by approximately 10% between social and intermediate housing groups; equivalent to a reduction of 111 for daily steps, 0.5 minutes for MVPA and 0.5kg/$m^2$ for BMI. However, a larger proportion of the difference in PA was apparent at weekends; equivalent to a reduction of 222 for daily steps and 2.2 minutes for MVPA.

**Relation to previous studies**

Studies have shown that lower socioeconomic status is associated with lower levels of PA (35;36), and that those from more socially deprived backgrounds have the most barriers to being physically active (7). Previous research examining the role of housing tenure is limited. Findings from this study showed marked differences in PA and adiposity between those seeking social, intermediate and market-rent housing. In particular, lower PA and higher adiposity in participants seeking social housing, a group which comprises a high proportion of people from more socioeconomically disadvantaged backgrounds (28). The higher levels of BMI and fat mass % in those seeking social housing compared with those seeking intermediate or market-rent housing is consistent with systematic reviews which have found an association between lower socioeconomic status and higher levels of adiposity, particularly in higher income countries and among women (37). While socioeconomic status is a strong determinant of housing status, to our knowledge this is the first study to


For peer review only - http://bmjopen.bmj.com/site/about/guidelines.xhtml

explicitly examine housing sector differences in objective PA and markers of adiposity levels (i.e. BMI and fat mass %). However, it is important to consider more broadly what these aspirational housing sector differences might represent. Related studies have shown that those in social housing are less likely to use active travel compared with owner occupiers (18), and that those in social housing and home owners with a mortgage are more likely to be obese and have higher levels of illness and disability compared to outright home owners, even after adjustment for other socioeconomic status markers (38). These latter findings suggest that the effect of home ownership may be more complex and cannot be simply explained by socioeconomic status. Neighbourhood quality may offer a potential partial explanation for these findings (39). In the present study perceptions of better neighbourhood quality were associated with PA whereas perceptions of crime were not. In contrast, a large UK-based study found that perceptions of feeling safe in the neighbourhood had the largest effect on levels of PA compared with perceptions of leisure facilities, sense of belonging or access to public transport or amenities (40). Another study in the US found that low perceived safety from crime was associated with lower levels of MVPA (41). However, a recent review concluded that higher quality evidence is needed, including prospective studies and natural experiments in areas of wide crime variability, in order to further understand the effect of crime on physical and mental health (27). Moreover, previous work has suggested that objective and perceived measures of the built environment correlate differently with physical activity levels, suggesting that these measures are assessing different dimensions of the built environment which relate differently to health behaviour (42).


For peer review only - http://bmjopen.bmj.com/site/about/guidelines.xhtml

Our findings showed that PA levels were particularly low on the weekend among those seeking social housing, which is consistent with findings from a systematic review which found that leisure-time PA (which may be more likely to occur on weekends) was lower amongst those from lower socioeconomic groups (8). This suggests that low-cost strategies to increase weekend PA may be particularly beneficial to more disadvantaged households. A free community-based program in Bogata Colombia, temporarily closed streets on Sundays to encourage PA amongst more disadvantaged local residents (43). A similar program has been trialled in the United States (44), however the effectiveness, longevity and generalisability of these programs to other socioeconomically deprived areas is yet to be established.

**Strengths and limitations**

Strengths of this study include the representation of three different aspirational housing groups which provides a wide range of socioeconomic backgrounds. Of those seeking social housing, two-thirds (67%) were currently living in social housing accommodation provided by the local authority or housing association; the remainder were largely currently living in privately rented accommodation with many on social housing waiting lists. Of those seeking intermediate or market-rent accommodation, almost two-thirds were living in privately rented accommodation (both 64%); the remainder were largely living with relatives or friends. The study sample is large with good representation from a 'hard to reach' group of social housing participants. Participation rates were high given the target group, with between 50-60% of those who initially agreed to be contacted taking part in the study. The ActiGraph GT3X+ accelerometer provided validated objective measures of PA (45) and the use of bioelectrical impedance to provide more direct measurements of adiposity including


For peer review only - http://bmjopen.bmj.com/site/about/guidelines.xhtml
For peer review only

fat mass %, which may provide a more valid marker of adiposity than BMI, particularly in a multi-ethnic population (46;47). Reassuringly the patterns of PA by sex, ethnic group and health status were consistent with those published previously (48-50). A limitation of the study is the lower number of participants in the market-rent sector compared with the other groups. This was due to restrictions imposed on the study team on the extent and duration of access to potential applicants seeking market-rent accommodation. While the study is longitudinal, these analyses are cross-sectional limiting the degree to which causal inferences can be made. Moreover, there is the possibility of selection amongst study participants, where those who are more active seek to move to East Village, may be more likely to participate in the study and may perceive their environment differently, which may limit the generalisability of the findings to neighbourhoods outside of East London.

**Conclusions and future work**

The findings presented in this paper suggest that perceived neighbourhood quality is associated with meaningful differences in PA and markers of adiposity. Differences in steps (680 steps) and BMI (3.6kg/m$^2$) between the lowest and highest quintiles of perceived neighbourhood quality should be considered in the context of an average 10,000 steps per day, where a 5% increase (500 steps) would be a worthwhile population level increase and a 5kg/m$^2$ increase in BMI is associated with a 31% increase in all-cause mortality (51). Hence, improvements in neighbourhood quality could be associated with health benefits of public health importance. There were also substantial differences in PA, BMI and fat mass % between the three housing groups studied. In particular the very low levels of PA in the social housing group during the weekend could provide a target for intervention to increase levels of PA; again these differences should be considered in relation to 500 steps per day,



For peer review only - http://bmjopen.bmj.com/site/about/guidelines.xhtml

which can be considered as an increase of population importance. Perceptions of neighbourhood quality reduced differences in PA and adiposity between housing sector groups, and the possibility of measuring more objective markers of neighbourhood quality within this study has the potential to explain more (42). The future follow-up of the ENABLE London cohort will allow us to examine whether moving to 'East Village', a neighbourhood designed for healthy active living, will have a positive impact on PA and/or adiposity levels. A major aim of the study is to identify features of the local built environment that increase levels of PA which could potentially help to reduce socioeconomic inequalities in health. It will be of particular interest to determine whether an increase in PA is more apparent in the social housing group whose neighbourhood characteristics should improve. Furthermore, we will be in a position to examine whether any potential effects of the built environment on PA are modified by housing sector type.



For peer review only - http://bmjopen.bmj.com/site/about/guidelines.xhtml

Ethical approval

Full ethical approval was obtained from the relevant Multi-Centre Research Ethics Committee (REC Reference 12/LO/1031). All participants provided written informed consent.

Data sharing statement

Further details of the ENABLE London study are available from the study website (http://www.enable.sgul.ac.uk/). We welcome proposals for collaborative projects. For general data sharing inquiries, contact Professor Owen (cowen@sgul.ac.uk).

Competing interests

We declare that we have no competing interests.

Funding

This research is being supported by project grants from the Medical Research Council National Prevention Research Initiative (MR/J000345/1) and National Institute for Health Research (12/211/69). Diabetes and obesity prevention research at St George's, University of London is supported by the National Institute for Health Research (NIHR) Collaboration for Leadership in Applied Health Research and Care (CLAHRC) South London. AE is funded by the UK Medical Research Council as part of the Neighbourhoods and Communities Programme (MC_UU_12017-10). BG-C is supported by an NHMRC Principal Research Fellowship (#1107672). ARC and ASP are supported by NIHR Biomedical Research Centre at University Hospitals Bristol NHS Foundation Trust and the University of Bristol. The views



For peer review only - http://bmjopen.bmj.com/site/about/guidelines.xhtml

expressed in this publication are those of the author(s) and not necessarily those of the NHS, the National Institute for Health Research or the Department of Health.

Authorship statement

CGO, ARR, AE, ARC, DL, SC, BG-C, DGC and PHW designed the study and raised funding. BR, ARR, CC, DP and CGO collected data for the study; BR, ARR and CGO enrolled participants. CMN, BR, ESL, ARR, CC, DP and CGO undertook data management. CMN, ESL analysed the data; CMN wrote the first draft of the report. ARR, BR, ESL, AS, DP, ARC, ASP, AE, BG-C, CC, DL, SC, PHW, DGC and CGO critically appraised the manuscript and approved the final draft. CGO is responsible for data integrity.

Acknowledgements

The authors thank the East Thames Group, Triathlon Homes and Get Living London who have assisted in recruiting participants into the ENABLE London study. The ENABLE London study is advised by a Steering Committee chaired by Professor Hazel Inskip (University of Southampton), with Dr David Ogilvie (University of Cambridge) and Professor Andy Jones (University of East Anglia) as academic advisors and Mrs Kate Worley (formerly East Thames Group Assistant Director for Strategic Housing) as the lay/stakeholder member. The authors are grateful to the members of the ENABLE London study team (in particular Aine Hogan, Katrin Peuker, Cathy McKay) and to participating households, without whom this study would not be possible.



For peer review only - http://bmjopen.bmj.com/site/about/guidelines.xhtml

Reference List

1. Ford ES, Williamson DF, Liu S. Weight change and diabetes incidence: findings from a national cohort of US adults. Am J Epidemiol 1997;146:214-22.

2. Nocon M, Hiemann T, Muller-Riemenschneider F, Thalau F, Roll S, Willich SN. Association of physical activity with all-cause and cardiovascular mortality: a systematic review and meta-analysis. Eur J Cardiovasc Prev Rehabil 2008;15:239-46.

3. Shaper AG, Wannamethee SG, Walker M. Body weight: implications for the prevention of coronary heart disease, stroke, and diabetes mellitus in a cohort study of middle aged men. BMJ 1997;314:1311-7.

4. Whitlock G, Lewington S, Sherliker P, Clarke R, Emberson J, Halsey J, Qizilbash N, Collins R, Peto R. Body-mass index and cause-specific mortality in 900 000 adults: collaborative analyses of 57 prospective studies. Lancet 2009;373:1083-96.

5. Lim SS, Vos T, Flaxman AD, Danaei G, Shibuya K, Adair-Rohani H, Amann M, Anderson HR, Andrews KG, Aryee M et al. A comparative risk assessment of burden of disease and injury attributable to 67 risk factors and risk factor clusters in 21 regions, 1990-2010: a systematic analysis for the Global Burden of Disease Study 2010. Lancet 2012;380:2224-60.

6. Craig R, Mindell J. Health Survey for England 2012: Health, social care and lifestyles. Health and Social Care Information Centre, Leeds, 2013, 2013.

7. Chinn DJ, White M, Harland J, Drinkwater C, Raybould S. Barriers to physical activity and socioeconomic position: implications for health promotion. J Epidemiol Community Health 1999;53:191-2.

8. Beenackers MA, Kamphuis CB, Giskes K, Brug J, Kunst AE, Burdorf A, van Lenthe FJ. Socioeconomic inequalities in occupational, leisure-time, and transport related physical activity among European adults: a systematic review. Int J Behav Nutr Phys Act 2012;9:116.

9. McVeigh JA, Winkler EA, Howie EK, Tremblay MS, Smith A, Abbott RA, Eastwood PR, Healy GN, Straker LM. Objectively measured patterns of sedentary time and physical activity in young adults of the Raine study cohort. Int J Behav Nutr Phys Act 2016;13:41.

10. Sigmundova D, Sigmund E, Vokacova J, Kopkova J. Parent-child associations in pedometer-determined physical activity and sedentary behaviour on weekdays and weekends in random samples of families in the Czech Republic. Int J Environ Res Public Health 2014;11:7163-81.

11. Ellaway A, Macdonald L, Kearns A. Are housing tenure and car access still associated with health? A repeat cross-sectional study of UK adults over a 13-year period. BMJ Open 2016;6:e012268.

12. Macintyre S, Hiscock R, Kearns A, Ellaway A. Housing tenure and car access: further exploration of the nature of their relations with health in a UK setting. J Epidemiol Community Health 2001;55:330-1.

13. Smith GD, Egger M. Socioeconomic differences in mortality in Britain and the United States. Am J Public Health 1992;82:1079-81.



For peer review only - http://bmjopen.bmj.com/site/about/guidelines.xhtml

14. Dalstra JA, Kunst AE, Mackenbach JP. A comparative appraisal of the relationship of education, income and housing tenure with less than good health among the elderly in Europe. Soc Sci Med 2006;62:2046-60.

15. Macintyre S, McKay L, Der G, Hiscock R. Socio-economic position and health: what you observe depends on how you measure it. J Public Health Med 2003;25:288-94.

16. Harrison RA, McElduff P, Edwards R. Planning to win: Health and lifestyles associated with physical activity amongst 15,423 adults. Public Health 2006;120:206-12.

17. Kendig H, Browning C, Teshuva K. Health actions and social class among older Australians. Aust N Z J Public Health 1998;22:808-13.

18. Ogilvie D, Mitchell R, Mutrie N, Petticrew M, Platt S. Personal and environmental correlates of active travel and physical activity in a deprived urban population. Int J Behav Nutr Phys Act 2008;5:43.

19. Boniface DR, Cottee MJ, Neal D, Skinner A. Social and demographic factors predictive of change over seven years in CHD-related behaviours in men aged 18-49 years. Public Health 2001;115:246-52.

20. Macintyre S, Ellaway A, Hiscock R, Kearns A, Der G, McKay L. What features of the home and the area might help to explain observed relationships between housing tenure and health? Evidence from the west of Scotland. Health Place 2003;9:207-18.

21. Ellaway A, Macintyre S. Does housing tenure predict health in the UK because it exposes people to different levels of housing related hazards in the home or its surroundings? Health Place 1998;4:141-50.

22. Macintyre S, Ellaway A, Der G, Ford G, Hunt K. Do housing tenure and car access predict health because they are simply markers of income or self esteem? A Scottish study. J Epidemiol Community Health 1998;52:657-64.

23. Hoehner CM, Brennan Ramirez LK, Elliott MB, Handy SL, Brownson RC. Perceived and objective environmental measures and physical activity among urban adults. Am J Prev Med 2005;28:105-16.

24. Sallis JF, Bowles HR, Bauman A, Ainsworth BE, Bull FC, Craig CL, Sjostrom M, De B, I, Lefevre J, Matsudo V et al. Neighborhood environments and physical activity among adults in 11 countries. Am J Prev Med 2009;36:484-90.

25. Giles-Corti B, Ryan K, Foster S. Increasing density in Australia: maximising the health benefits and minimising harm. https://www.heartfoundation.org.au/images/uploads/publications/Increasing-density-in-Australia-Evidence-Review-2012-trevor.pdf [Accessed August 2017], 2012.

26. Foster S, Giles-Corti B. The built environment, neighborhood crime and constrained physical activity: an exploration of inconsistent findings. Prev Med 2008;47:241-51.

27. da Silva IC, Payne VL, Hino AA, Varela AR, Reis RS, Ekelund U, Hallal PC. Physical Activity and Safety From Crime Among Adults: A Systematic Review. J Phys Act Health 2016;13:663-70.



For peer review only - http://bmjopen.bmj.com/site/about/guidelines.xhtml

28. Ram B, Nightingale CM, Hudda MT, Kapetanakis VV, Ellaway A, Cooper AR, Page A, Lewis D, Cummins S, Giles-Corti B et al. Cohort profile: Examining Neighbourhood Activities in Built Living Environments in London: the ENABLE London-Olympic Park cohort. BMJ Open 2016;6:e012643.

29. Ram B, Shankar A, Nightingale CM, Giles-Corti B, Ellaway A, Cooper AR, Page A, Cummins S, Lewis D, Whincup PH et al. Comparisons of depression, anxiety, well-being, and perceptions of the built environment amongst adults seeking social, intermediate and market-rent accommodation in the former London Olympic Athletes' Village. Health Place 2017;48:31-9.

30. Mason KE, Baker E, Blakely T, Bentley RJ. Housing affordability and mental health: does the relationship differ for renters and home purchasers? Soc Sci Med 2013;94:91-7.

31. Office for National Statistics. The National Statistics Socio-economic Classification User Manual. 2005.

32. Department of Health. Start active, stay active: a report on physical activity from the four home countries Chief Medical Officers. 2011. Department of Health.

33. Freedson PS, Melanson E, Sirard J. Calibration of the Computer Science and Applications, Inc. accelerometer. Med Sci Sports Exerc 1998;30:777-81.

34. Harris T, Kerry SM, Limb ES, Victor CR, Iliffe S, Ussher M, Whincup PH, Ekelund U, Fox-Rushby J, Furness C et al. Effect of a Primary Care Walking Intervention with and without Nurse Support on Physical Activity Levels in 45- to 75-Year-Olds: The Pedometer And Consultation Evaluation (PACE-UP) Cluster Randomised Clinical Trial. PLoS Med 2017;14:e1002210.

35. Allen MS, Vella SA. Longitudinal determinants of walking, moderate, and vigorous physical activity in Australian adults. Prev Med 2015;78:101-4.

36. Juneau CE, Sullivan A, Dodgeon B, Cote S, Ploubidis GB, Potvin L. Social class across the life course and physical activity at age 34 years in the 1970 British birth cohort. Ann Epidemiol 2014;24:641-7, 647.

37. McLaren L. Socioeconomic status and obesity. Epidemiol Rev 2007;29:29-48.

38. Tranter B, Donoghue J. Housing tenure, body mass index and health in Australia. International Journal of Housing Policy 2016;1:1-20.

39. Turrell G, Haynes M, Wilson LA, Giles-Corti B. Can the built environment reduce health inequalities? A study of neighbourhood socioeconomic disadvantage and walking for transport. Health Place 2013;19:89-98.

40. Harrison RA, Gemmell I, Heller RF. The population effect of crime and neighbourhood on physical activity: an analysis of 15,461 adults. J Epidemiol Community Health 2007;61:34-9.

41. Brown BB, Werner CM, Smith KR, Tribby CP, Miller HJ. Physical activity mediates the relationship between perceived crime safety and obesity. Prev Med 2014;66:140-4.

42. McGinn AP, Evenson KR, Herring AH, Huston SL, Rodriguez DA. Exploring associations between physical activity and perceived and objective measures of the built environment. J Urban Health 2007;84:162-84.



For peer review only - http://bmjopen.bmj.com/site/about/guidelines.xhtml

43. Sarmiento O, Torres A, Jacoby E, Pratt M, Schmid TL, Stierling G. The Ciclovia-Recreativa: A mass-recreational program with public health potential. J Phys Act Health 2010;7 Suppl 2:S163-S180.

44. Hipp JA, Eyler AA, Zieff SG, Samuelson MA. Taking physical activity to the streets: the popularity of Ciclovia and Open Streets initiatives in the United States. Am J Health Promot 2014;28:S114-S115.

45. Santos-Lozano A, Santin-Medeiros F, Cardon G, Torres-Luque G, Bailon R, Bergmeir C, Ruiz JR, Lucia A, Garatachea N. Actigraph GT3X: validation and determination of physical activity intensity cut points. Int J Sports Med 2013;34:975-82.

46. Deurenberg P, Yap M, van Staveren WA. Body mass index and percent body fat: a meta analysis among different ethnic groups. Int J Obes Relat Metab Disord 1998;22:1164-71.

47. Gallagher D, Visser M, Sepulveda D, Pierson RN, Harris T, Heymsfield SB. How useful is body mass index for comparison of body fatness across age, sex, and ethnic groups? Am J Epidemiol 1996;143:228-39.

48. Hayes L, White M, Unwin N, Bhopal R, Fischbacher C, Harland J, Alberti KG. Patterns of physical activity and relationship with risk markers for cardiovascular disease and diabetes in Indian, Pakistani, Bangladeshi and European adults in a UK population. J Public Health Med 2002;24:170-8.

49. Health and Social Care Information Centre. Health Survey for England 2004: The health of ethnic minority groups. Sproston, K. and Mindell, J. 2006. The Information Centre.

50. Health and Social Care Information Centre. Health Survey for England - 2008: Physical activity and fitness. Craig R, Mindell J, and Hirani V. 2009. NHS Digital.

51. Global BMI Mortality Collaboration, Di AE, Bhupathiraju S, Wormser D, Gao P, Kaptoge S, Berrington de GA, Cairns BJ, Huxley R, Jackson C et al. Body-mass index and all-cause mortality: individual-participant-data meta-analysis of 239 prospective studies in four continents. Lancet 2016;388:776-86.

For peer review only



For peer review only - http://bmjopen.bmj.com/site/about/guidelines.xhtml

Table 1: Associations between adiposity and physical activity outcomes and patient characteristics

| | | Difference or % difference* in adiposity/physical activity (95% CI), p-value | | | | | | | | | | | | | | |
|---|---|---|---|---|---|---|---|---|---|---|---|---|---|---|---|---|
| | n | BMI (kg/m$^2$)* | | | Fat mass % | | | Daily steps† | | | Daily minutes spent in MVPA† | | | Daily minutes spent in MVPA in ≥10 minute bouts† | | |
| **Sex** | | | | | | | | | | | | | | | | |
| Male (Ref) | 522 | | | - | | | - | | | - | | | - | | | - |
| Female | 718 | -1.2 | (-3.2, 0.9) | 0.26 | 11.1 | (10.3, 12.0) | <0.0001 | -570 | (-946, -194) | 0.003 | -9.3 | (-12.2, -6.4) | <0.0001 | -4.1 | (-6.1, -2.0) | <0.001 |
| **Age group** | | | | | | | | | | | | | | | | |
| Age 16-24 (Ref) | 269 | | | - | | | - | | | - | | | - | | | - |
| Age 25-34 | 531 | 6.3 | (3.5, 9.1) | <0.0001 | 3.2 | (2.1, 4.3) | <0.0001 | 502 | (11, 992) | 0.04 | 4.0 | (0.2, 7.9) | 0.04 | 1.0 | (-1.9, 3.8) | 0.51 |
| Age 35-49 | 358 | 13.4 | (10.2, 16.6) | <0.0001 | 6.4 | (5.2, 7.6) | <0.0001 | 699 | (173, 1224) | 0.01 | 3.9 | (-0.2, 8.0) | 0.07 | -1.1 | (-4.0, 1.8) | 0.46 |
| Age 50+ | 82 | 17.6 | (12.6, 22.9) | <0.0001 | 9.2 | (7.3, 11.0) | <0.0001 | -9 | (-832, 813) | 0.98 | -6.0 | (-12.4, 0.5) | 0.07 | -2.0 | (-6.8, 2.7) | 0.40 |
| **Ethnic group** | | | | | | | | | | | | | | | | |
| White (Ref) | 595 | | | - | | | - | | | - | | | - | | | - |
| Black | 314 | 6.2 | (3.3, 9.3) | <0.0001 | 3.6 | (2.4, 4.8) | <0.0001 | -1116 | (-1657, -575) | <0.0001 | -7.4 | (-11.7, -3.2) | <0.001 | -6.6 | (-9.8, -3.4) | <0.0001 |
| Asian | 210 | -0.3 | (-3.1, 2.7) | 0.85 | 0.02 | (-1.2, 1.3) | 0.97 | -1409 | (-1972, -845) | <0.0001 | -11.5 | (-15.9, -7.0) | <0.0001 | -8.1 | (-11.4, -4.8) | <0.0001 |
| Other/Mixed | 121 | 1.3 | (-2.3, 5.0) | 0.48 | 1.0 | (-0.5, 2.5) | 0.18 | -430 | (-1100, 239) | 0.21 | -4.6 | (-9.8, 0.7) | 0.09 | -4.0 | (-7.9, -0.04) | 0.05 |
| **Limiting illness** | | | | | | | | | | | | | | | | |
| No (Ref) | 1087 | | | - | | | - | | | - | | | - | | | - |
| Yes | 153 | 4.3 | (1.1, 7.5) | 0.01 | 1.6 | (0.3, 2.9) | 0.01 | -1081 | (-1666, -496) | <0.001 | -5.7 | (-10.3, -1.1) | 0.01 | -2.8 | (-6.1, 0.5) | 0.10 |
| **Housing sector** | | | | | | | | | | | | | | | | |
| Social | 512 | 5.0 | (2.2, 7.8) | <0.001 | 2.7 | (1.5, 3.8) | <0.0001 | -1125 | (-1629, -620) | <0.0001 | -7.5 | (-11.5, -3.6) | <0.001 | -6.5 | (-9.5, -3.5) | <0.0001 |
| Intermediate (Ref) | 503 | | | - | | | - | | | - | | | - | | | - |
| Market-rent | 225 | -0.8 | (-3.6, 2.0) | 0.57 | -0.2 | (-1.4, 1.0) | 0.70 | -104 | (-633, 424) | 0.70 | 2.3 | (-1.9, 6.4) | 0.29 | 2.8 | (-0.3, 6.0) | 0.08 |

* Percentage differences are presented for BMI, which was log-transformed for analysis

All differences and % differences are mutually adjusted for sex, age group, ethnic group, limiting longstanding illness, housing sector and a random effect to allow for clustering at household level

† Missing data for 133 participants

MVPA and MVPA in ≥10 minute bouts are an average daily estimate, obtained from averaging a participant's weekly total.



For peer review only

For peer review only - http://bmjopen.bmj.com/site/about/guidelines.xhtml

Table 2: Associations between adiposity and physical activity outcomes and neighbourhood perceptions scales

| | Difference or % difference* in outcome between the highest and lowest quintiles for each neighbourhood scale (95% CI), p-value | | | | | |
|---|---|---|---|---|---|---|
| | Perceptions of NH quality | | | Perceptions of NH crime | | |
| Adiposity (N = 1240) | | | | | | |
| Body mass index ($kg/m^2$)* | -3.6 | (-6.5, -0.6) | 0.02 | -2.1 | (-5.4, 1.3) | 0.21 |
| Fat mass % | -1.2 | (-2.5, 0.06) | 0.06 | -0.8 | (-2.2, 0.7) | 0.30 |
| Physical activity (N = 1107) | | | | | | |
| Daily steps | 677 | (108, 1247) | 0.02 | -63 | (-713, 587) | 0.85 |
| Daily MVPA (minutes) | 4.5 | (0.02, 9.0) | 0.05 | 1.1 | (-4.0, 6.2) | 0.68 |
| Daily MVPA in ≥10 minute bouts (minutes) | 2.7 | (-0.6, 6.0) | 0.11 | 2.4 | (-1.4, 6.1) | 0.22 |

* Percentage differences are presented for BMI, which was log-transformed for analysis
All differences and % differences are adjusted for sex, age group, ethnic group, limiting longstanding illness, housing sector and a random effect to allow for clustering at household level.
Abbreviations: NH, neighbourhood
MVPA and MVPA in ≥10 minute bouts are an average daily estimate, obtained from averaging a participant's weekly total.

For peer review only



For peer review only - http://bmjopen.bmj.com/site/about/guidelines.xhtml

Table 3: Adiposity and physical activity differences between housing sectors: adjustment for perceptions of neighbourhood quality

| | Housing sector group | Difference or % difference* compared to intermediate housing group (95% confidence interval), p-value — Model 1 | | | Model 2 (Additionally adjusted for neighbourhood quality scale) | | |
|---|---|---|---|---|---|---|---|
| Adiposity (N = 1240) | | | | | | | |
| Body mass index (kg/m$^2$)* | Social | 5.0 | (2.2, 7.8) | <0.001 | 4.5 | (1.7, 7.3) | 0.002 |
| | Intermediate | Reference group | | | | | |
| | Market rent | -0.8 | (-3.6, 2.0) | 0.57 | -0.9 | (-3.6, 2.0) | 0.55 |
| Fat mass % | Social | 2.7 | (1.5, 3.8) | <0.0001 | 2.5 | (1.4, 3.6) | <0.0001 |
| | Intermediate | Reference group | | | | | |
| | Market rent | -0.2 | (-1.4, 1.0) | 0.70 | -0.2 | (-1.4, 0.9) | 0.68 |
| Physical activity (N = 1107) | | | | | | | |
| Daily steps | Social | -1125 | (-1629, -620) | <0.0001 | -1016 | (-1531, -501) | <0.001 |
| | Intermediate | Reference group | | | | | |
| | Market rent | -104 | (-633, 424) | 0.70 | -96 | (-624, 431) | 0.72 |
| Daily MVPA (minutes) | Social | -7.5 | (-11.5, -3.6) | <0.001 | -6.8 | (-10.8, -2.7) | 0.001 |
| | Intermediate | Reference group | | | | | |
| | Market rent | 2.3 | (-1.9, 6.4) | 0.29 | 2.3 | (-1.8, 6.5) | 0.27 |
| Daily MVPA in ≥10 minute bouts (minutes) | Social | -6.5 | (-9.5, -3.5) | <0.0001 | -6.0 | (-9.1, -3.0) | <0.001 |
| | Intermediate | Reference group | | | | | |
| | Market rent | 2.8 | (-0.3, 6.0) | 0.08 | 2.8 | (-0.3, 6.0) | 0.08 |

* Percentage differences are presented for BMI, which was log-transformed for analysis
Model 1: Adjusted for sex, age group, ethnic group, limiting longstanding illness and clustering at household level (random effect)
Model 2: Adjusted as Model 1 plus neighbourhood quality scale (added as a continuous variable)
MVPA and MVPA in ≥10 minute bouts are an average daily estimate, obtained from averaging a participant's weekly total.



For peer review only - http://bmjopen.bmj.com/site/about/guidelines.xhtml

Figure 1: Daily physical activity by day of the week and housing sector group: N = 6206 days from 1107 participants

Means and 95% confidence intervals are adjusted for sex, age group, ethnic group, limiting longstanding illness, month of recording, day order of recording, day of week, housing sector, an interaction between housing sector and day of week and random effects to allow for multiple days of measurement and clustering of participants within households

For peer review only



For peer review only - http://bmjopen.bmj.com/site/about/guidelines.xhtml

For peer review only



For peer review only - http://bmjopen.bmj.com/site/about/guidelines.xhtml

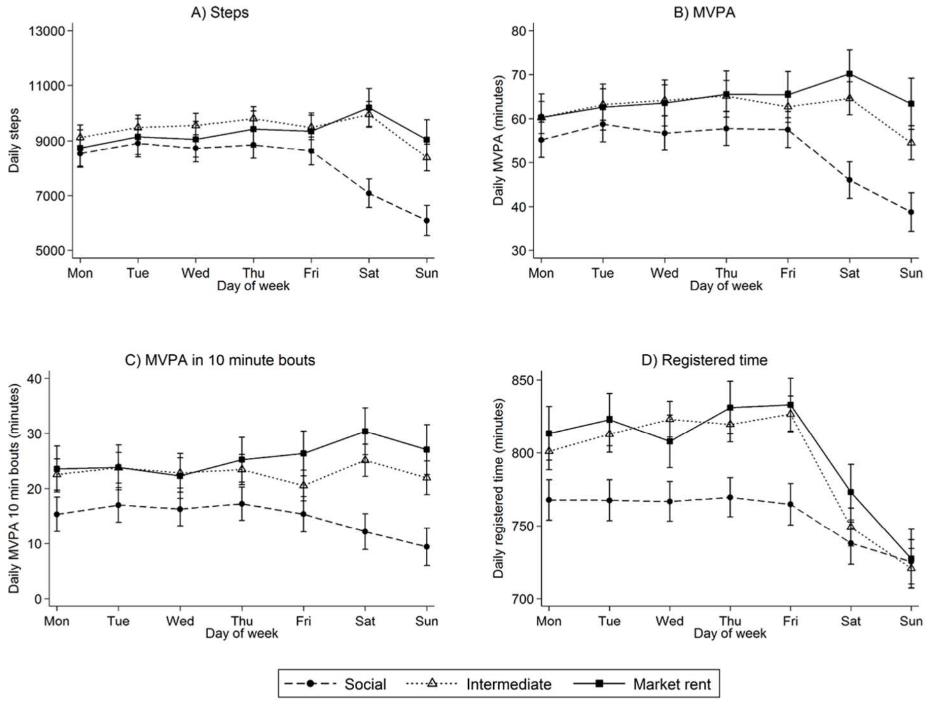


Figure 1: Daily physical activity by day of the week and housing sector group: N = 6206 days from 1107 participants

119x90mm (300 x 300 DPI)

**Supplementary Table 1: Questionnaire items included in the factor analysis on perceptions of the neighbourhood**

| |
|---|
| **Perceptions of neighbourhood crime items** |
| There is a lot of crime in my neighbourhood. |
| The level of crime in my neighbourhood makes it unsafe to walk on the streets at night. |
| There are threatening groups of young people in my neighbourhood. |
| The level of crime in my neighbourhood makes it unsafe to walk on the streets during the day. |
| Vandalism, graffiti or deliberate damage to property is a problem in my local area. |
| **Perceptions of neighbourhood quality items** |
| I enjoy walking in my neighbourhood. |
| This area is a place I enjoy living in. |
| My neighbourhood is attractive to look at (e.g. there are attractive buildings, green space. Landscaping views). |
| This area has good leisure things for people like myself, leisure centres or community centres for example. |
| You often see people out on walks or riding their bicycles in my neighbourhood. |
| This area has good local transport. |
| **Additional items included in the factor analysis with factor loadings below 0.4 and were therefore not included in the solution** |
| My neighbourhood is generally free from litter. |
| There is too much traffic in my neighbourhood. |
| Our neighbourhood streets have good lighting at night. |

Participants were asked to select a response from the following for all questionnaire items stated in the table: Strongly agree, Agree, Neither agree nor disagree, Disagree, Strongly disagree

For peer review only

**Supplementary Table 2: Participant characteristics for 1240 adults with measurements of adiposity at baseline**

| | Housing sector | | | | | | | | |
|---|---|---|---|---|---|---|---|---|---|
| | Social | | Intermediate | | Market rent | | Total | | |
| | (n = 512) | | (n = 503) | | (n = 225) | | (N = 1240) | | p ($\chi^2$) |
| **Sex** | | | | | | | | | |
| Male | 137 | (26.8%) | 259 | (51.5%) | 126 | (56.0%) | 522 | (42.1%) | |
| Female | 375 | (73.2%) | 244 | (48.5%) | 99 | (44.0%) | 718 | (57.9%) | <0.0001 |
| **Age group** | | | | | | | | | |
| 16-24 | 107 | (20.9%) | 92 | (18.3%) | 70 | (31.1%) | 269 | (21.7%) | |
| 25-34 | 129 | (25.2%) | 291 | (57.9%) | 111 | (49.3%) | 531 | (42.8%) | |
| 35-49 | 233 | (45.5%) | 102 | (20.3%) | 23 | (10.2%) | 358 | (28.9%) | |
| 50+ | 43 | (8.4%) | 18 | (3.6%) | 21 | (9.3%) | 82 | (6.6%) | <0.0001 |
| **Ethnic group** | | | | | | | | | |
| White | 96 | (18.8%) | 342 | (68.0%) | 157 | (69.8%) | 595 | (48.0%) | |
| Black | 245 | (47.9%) | 53 | (10.5%) | 16 | (7.1%) | 314 | (25.3%) | |
| Asian | 107 | (20.9%) | 75 | (14.9%) | 28 | (12.4%) | 210 | (16.9%) | |
| Mixed/Other | 64 | (12.5%) | 33 | (6.6%) | 24 | (10.7%) | 121 | (9.8%) | <0.0001 |
| **NS-SEC*** | | | | | | | | | |
| Higher Managerial / Professional | 60 | (11.9%) | 357 | (71.4%) | 150 | (66.7%) | 567 | (46.1%) | |
| Intermediate Occupations | 62 | (12.3%) | 77 | (15.4%) | 38 | (16.9%) | 177 | (14.4%) | |
| Routine / Manual | 125 | (24.8%) | 34 | (6.8%) | 10 | (4.4%) | 169 | (13.7%) | |
| Economically inactive | 258 | (51.1%) | 32 | (6.4%) | 27 | (12.0%) | 317 | (25.8%) | <0.0001 |
| **Limiting illness** | | | | | | | | | |
| Yes | 102 | (19.9%) | 40 | (8.0%) | 11 | (4.9%) | 153 | (12.3%) | |
| No | 410 | (80.1%) | 463 | (92.0%) | 214 | (95.1%) | 1087 | (87.7%) | <0.0001 |

p ($\chi^2$): p-value for Chi-squared test
* 10 responses missing for NS-SEC group

For peer review only

**Supplementary Table 3: Mean levels of adiposity and physical activity by patient characteristics**

| | | Mean/Geometric mean* levels adiposity and physical activity (95% confidence intervals) | | | | | | | | | |
|---|---|---|---|---|---|---|---|---|---|---|---|
| | n | BMI (kg/m$^2$)* | | Fat mass % | | Daily steps† | | Daily minutes of MVPA† | | Daily minutes of MVPA in ≥10 minute bouts† | |
| **Sex** | | | | | | | | | | | |
| Male | 522 | 25.4 | (25.0, 25.8) | 20.4 | (19.7, 21.0) | 9279 | (8991, 9568) | 64.8 | (62.6, 67.1) | 22.8 | (21.1, 24.4) |
| Female | 718 | 25.1 | (24.8, 25.5) | 31.5 | (30.9, 32.0) | 8709 | (8464, 8954) | 55.6 | (53.6, 57.5) | 18.7 | (17.3, 20.1) |
| **Age group** | | | | | | | | | | | |
| Age 16-24 | 269 | 23.5 | (23.0, 24.0) | 23.0 | (22.1, 23.9) | 8534 | (8136, 8932) | 57.0 | (53.9, 60.2) | 20.5 | (18.2, 22.8) |
| Age 25-34 | 531 | 25.0 | (24.6, 25.3) | 26.2 | (25.5, 26.8) | 9035 | (8744, 9326) | 61.1 | (58.8, 63.3) | 21.4 | (19.7, 23.1) |
| Age 35-49 | 358 | 26.6 | (26.1, 27.1) | 29.3 | (28.5, 30.1) | 9232 | (8879, 9585) | 60.9 | (58.1, 63.6) | 19.4 | (17.3, 21.4) |
| Age 50+ | 82 | 27.6 | (26.6, 28.7) | 32.2 | (30.5, 33.8) | 8525 | (7800, 9249) | 51.1 | (45.4, 56.7) | 18.4 | (14.2, 22.7) |
| **Ethnic group** | | | | | | | | | | | |
| White | 595 | 24.9 | (24.5, 25.2) | 25.8 | (25.1, 26.4) | 9491 | (9203, 9779) | 63.6 | (61.3, 65.8) | 23.7 | (22.0, 25.4) |
| Black | 314 | 26.4 | (25.8, 27.0) | 29.4 | (28.5, 30.3) | 8375 | (7961, 8789) | 56.2 | (52.9, 59.4) | 17.1 | (14.7, 19.6) |
| Asian | 210 | 24.8 | (24.2, 25.4) | 25.8 | (24.8, 26.8) | 8082 | (7608, 8556) | 52.1 | (48.4, 55.8) | 15.6 | (12.8, 18.4) |
| Other/Mixed | 121 | 25.2 | (24.4, 26.0) | 26.8 | (25.5, 28.1) | 9060 | (8465, 9656) | 59.0 | (54.3, 63.7) | 19.8 | (16.3, 23.3) |
| **Limiting illness** | | | | | | | | | | | |
| No | 1087 | 25.1 | (24.9, 25.4) | 26.6 | (26.1, 27.0) | 9077 | (8877, 9277) | 60.1 | (58.6, 61.7) | 20.8 | (19.6, 21.9) |
| Yes | 153 | 26.2 | (25.5, 27.0) | 28.2 | (27.0, 29.4) | 7996 | (7447, 8545) | 54.4 | (50.1, 58.7) | 18.0 | (14.8, 21.1) |
| **Housing sector** | | | | | | | | | | | |
| Social | 512 | 26.0 | (25.6, 26.5) | 28.4 | (27.6, 29.1) | 8298 | (7953, 8642) | 54.6 | (51.8, 57.3) | 16.0 | (14.0, 18.1) |
| Intermediate | 503 | 24.8 | (24.4, 25.2) | 25.7 | (25.0, 26.4) | 9422 | (9110, 9735) | 62.1 | (59.6, 64.5) | 22.5 | (20.6, 24.4) |
| Market-rent | 225 | 24.6 | (24.0, 25.2) | 25.5 | (24.5, 26.5) | 9318 | (8863, 9773) | 64.3 | (60.8, 67.9) | 25.3 | (22.6, 28.1) |

* Geometric means are presented for BMI
All means/geometric means are adjusted for sex, age group, ethnic group, limiting longstanding illness, housing sector and a random effect to allow for clustering at household level.
† Data missing for 133 participants for average daily steps, MVPA and MVPA in bouts

For peer review only

**Supplementary Table 4: Associations between participant characteristics and physical activity variables in participants with at least 4 days of recording of physical activity data**

| | | Difference or % difference* in physical activity variable (95% confidence interval), p-value | | | | | | | | |
|---|---|---|---|---|---|---|---|---|---|---|
| | n | Daily steps | | | Daily minutes spent in MVPA | | | Daily minutes spent in MVPA in ≥10 minute bouts | | |
| **Sex** | | | | | | | | | | |
| Male (Ref) | 402 | - | | | - | | | - | | |
| Female | 529 | -559 | (-947, -172) | 0.005 | -9.7 | (-12.7, -6.7) | <0.0001 | -4.7 | (-6.8, -2.5) | <0.0001 |
| **Age group** | | | | | | | | | | |
| Age 16-24 (Ref) | 180 | - | | | - | | | - | | |
| Age 25-34 | 412 | 410 | (-116, 936) | 0.13 | 3.0 | (-1.2, 7.2) | 0.16 | -0.3 | (-3.3, 2.8) | 0.87 |
| Age 35-49 | 276 | 520 | (-45, 1086) | 0.07 | 2.5 | (-2.0, 6.9) | 0.27 | -2.5 | (-5.7, 0.7) | 0.12 |
| Age 50+ | 63 | -25 | (-889, 838) | 0.95 | -7.9 | (-14.8, -1.1) | 0.02 | -3.2 | (-8.2, 1.8) | 0.20 |
| **Ethnic group** | | | | | | | | | | |
| White (Ref) | 482 | - | | | - | | | - | | |
| Black | 214 | -1213 | (-1789, -638) | <0.0001 | -7.1 | (-11.7, -2.5) | 0.002 | -6.5 | (-9.9, -3.1) | <0.001 |
| Asian | 142 | -1128 | (-1719, -538) | <0.001 | -10.3 | (-15.0, -5.6) | <0.0001 | -7.8 | (-11.3, -4.3) | <0.0001 |
| Other/Mixed | 93 | -582 | (-1273, 110) | 0.10 | -4.7 | (-10.2, 0.8) | 0.09 | -4.2 | (-8.2, -0.2) | 0.04 |
| **Limiting illness** | | | | | | | | | | |
| No (Ref) | 834 | - | | | - | | | - | | |
| Yes | 97 | -976 | (-1612, -341) | 0.003 | -4.6 | (-9.6, 0.4) | 0.07 | -2.0 | (-5.6, 1.6) | 0.28 |
| **Housing sector** | | | | | | | | | | |
| Social | 332 | -978 | (-1515, -440) | <0.001 | -6.9 | (-11.2, -2.6) | 0.002 | -7.2 | (-10.4, -4.0) | <0.0001 |
| Intermediate (Ref) | 410 | - | | | - | | | - | | |
| Market-rent | 189 | -359 | (-889, 171) | 0.185 | 0.2 | (-4.0, 4.3) | 0.91 | 0.9 | (-2.2, 4.1) | 0.56 |

All differences are mutually adjusted for sex, age group, ethnic group, limiting longstanding illness, housing sector and a random effect to allow for clustering at household level.
MVPA and MVPA in ≥10 minute bouts are an average daily estimate, obtained from averaging a participant's weekly total.

For peer review only

**Supplementary Table 5: Physical activity differences between weekday (Monday-Friday) and weekend (Saturday, Sunday) activity by housing sector.**

| Physical activity variable (N = 1107) | Housing sector group | Mean (95% CI) weekday (Mon-Fri) activity | | Difference in PA outcome compared to weekdays (95% confidence interval), p-value | | | | | |
|---|---|---|---|---|---|---|---|---|---|
| | | | | Saturday - weekday | | | Sunday - weekday | | |
| Daily steps | Social | 8733 | (8364, 9103) | -1643 | (-2078, -1207) | <0.0001 | -2629 | (-3093, -2164) | <0.0001 |
| | Intermediate | 9497 | (9178, 9817) | 460 | (59, 862) | 0.02 | -1104 | (-1528, -680) | <0.0001 |
| | Market-rent | 9146 | (8673, 9619) | 1055 | (467, 1642) | <0.001 | -102 | (-734, 531) | 0.75 |
| MVPA (minutes) | Social | 57.2 | (54.3, 60.1) | -11.2 | (-14.7, -7.7) | <0.0001 | -18.4 | (-22.1, -14.7) | <0.0001 |
| | Intermediate | 63.1 | (60.6, 65.7) | 1.5 | (-1.8, 4.7) | 0.37 | -8.5 | (-11.9, -5.1) | <0.0001 |
| | Market-rent | 63.5 | (59.8, 67.3) | 6.6 | (1.9, 11.3) | 0.01 | -0.1 | (-5.2, 5.0) | 0.97 |
| MVPA in ≥10 minute bouts (minutes) | Social | 16.3 | (14.0, 18.5) | -4.1 | (-6.9, -1.3) | 0.004 | -6.8 | (-9.8, -3.9) | <0.0001 |
| | Intermediate | 22.6 | (20.7, 24.6) | 2.5 | (-0.06, 5.1) | 0.06 | -0.7 | (-3.4, 2.0) | 0.62 |
| | Market-rent | 24.2 | (21.3, 27.1) | 6.1 | (2.4, 9.9) | 0.001 | 2.8 | (-1.2, 6.9) | 0.17 |

Means and differences (95% confidence intervals) are adjusted for sex, age group, ethnic group, limiting longstanding illness, month of recording, day of the week, housing sector, an interaction between housing sector and day of week, and random effects to allow for multiple days of measurement and clustering of participants within household.

MVPA and MVPA in ≥10 minute bouts are an average daily estimate, obtained from averaging a participant's weekly total.

For peer review only

STROBE Statement—checklist of items that should be included in reports of observational studies

| | Item No | Recommendation | Page / line number at first call out |
|---|---|---|---|
| **Title and abstract** | 1 | (*a*) Indicate the study's design with a commonly used term in the title or the abstract | 2 / 7 |
| | | (*b*) Provide in the abstract an informative and balanced summary of what was done and what was found | 2 / 2 |
| **Introduction** | | | |
| Background/rationale | 2 | Explain the scientific background and rationale for the investigation being reported | 5 / 9 |
| Objectives | 3 | State specific objectives, including any prespecified hypotheses | 6 / 7 |
| **Methods** | | | |
| Study design | 4 | Present key elements of study design early in the paper | 6 / 16 |
| Setting | 5 | Describe the setting, locations, and relevant dates, including periods of recruitment, exposure, follow-up, and data collection | 7 / 4 |
| Participants | 6 | (*a*) *Cohort study*—Give the eligibility criteria, and the sources and methods of selection of participants. Describe methods of follow-up<br>*Case-control study*—Give the eligibility criteria, and the sources and methods of case ascertainment and control selection. Give the rationale for the choice of cases and controls<br>*Cross-sectional study*—Give the eligibility criteria, and the sources and methods of selection of participants | 6 / 18 |
| | | (*b*) *Cohort study*—For matched studies, give matching criteria and number of exposed and unexposed<br>*Case-control study*—For matched studies, give matching criteria and the number of controls per case | |
| Variables | 7 | Clearly define all outcomes, exposures, predictors, potential confounders, and effect modifiers. Give diagnostic criteria, if applicable | 8 / 5 |
| Data sources/ measurement | 8* | For each variable of interest, give sources of data and details of methods of assessment (measurement). Describe comparability of assessment methods if there is more than one group | *8 / 5* |
| Bias | 9 | Describe any efforts to address potential sources of bias | 10 / 14 |
| Study size | 10 | Explain how the study size was arrived at | 11 / 22 |
| Quantitative variables | 11 | Explain how quantitative variables were handled in the analyses. If applicable, describe which groupings were chosen and why | 8 / 6 |
| Statistical methods | 12 | (*a*) Describe all statistical methods, including those used to control for confounding | 10 / 8 |
| | | (*b*) Describe any methods used to examine subgroups and interactions | 10 / 12 |
| | | (*c*) Explain how missing data were addressed | Table 1 |
| | | (*d*) *Cohort study*—If applicable, explain how loss to follow-up was addressed<br>*Case-control study*—If applicable, explain how matching of cases | 10 / 8 |

For peer review only

| | | | |
|---|---|---|---|
| | | and controls was addressed<br>*Cross-sectional study*—If applicable, describe analytical methods taking account of sampling strategy | |
| | | (*e*) Describe any sensitivity analyses | 10 / 14 |
| **Results** | | | |
| Participants | 13* | (a) Report numbers of individuals at each stage of study—eg numbers potentially eligible, examined for eligibility, confirmed eligible, included in the study, completing follow-up, and analysed | 11 / 22 |
| | | (b) Give reasons for non-participation at each stage | 19 / 8 |
| | | (c) Consider use of a flow diagram | N / A |
| Descriptive data | 14* | (a) Give characteristics of study participants (eg demographic, clinical, social) and information on exposures and potential confounders | 12 / 7 |
| | | (b) Indicate number of participants with missing data for each variable of interest | Table 1 |
| | | (c) *Cohort study*—Summarise follow-up time (eg, average and total amount) | |
| Outcome data | 15* | *Cohort study*—Report numbers of outcome events or summary measures over time | |
| | | *Case-control study*—Report numbers in each exposure category, or summary measures of exposure | |
| | | *Cross-sectional study*—Report numbers of outcome events or summary measures | *12 / 2* |
| Main results | 16 | (*a*) Give unadjusted estimates and, if applicable, confounder-adjusted estimates and their precision (eg, 95% confidence interval). Make clear which confounders were adjusted for and why they were included | 12 /13 |
| | | (*b*) Report category boundaries when continuous variables were categorized | 12 /13 |
| | | (*c*) If relevant, consider translating estimates of relative risk into absolute risk for a meaningful time period | N / A |
| Other analyses | 17 | Report other analyses done—eg analyses of subgroups and interactions, and sensitivity analyses | 13 / 14 |
| **Discussion** | | | |
| Key results | 18 | Summarise key results with reference to study objectives | 15 / 18 |
| Limitations | 19 | Discuss limitations of the study, taking into account sources of potential bias or imprecision. Discuss both direction and magnitude of any potential bias | 19 / 3 |
| Interpretation | 20 | Give a cautious overall interpretation of results considering objectives, limitations, multiplicity of analyses, results from similar studies, and other relevant evidence | 19 / 14 |
| Generalisability | 21 | Discuss the generalisability (external validity) of the study results | 19 / 19 |
| **Other information** | | | |
| Funding | 22 | Give the source of funding and the role of the funders for the present study and, if applicable, for the original study on which the present article is based | 21 / 16 |

*Give information separately for cases and controls in case-control studies and, if applicable, for exposed and unexposed groups in cohort and cross-sectional studies.

For peer review only

**Note:** An Explanation and Elaboration article discusses each checklist item and gives methodological background and published examples of transparent reporting. The STROBE checklist is best used in conjunction with this article (freely available on the Web sites of PLoS Medicine at http://www.plosmedicine.org/, Annals of Internal Medicine at http://www.annals.org/, and Epidemiology at http://www.epidem.com/). Information on the STROBE Initiative is available at www.strobe-statement.org.

For peer review only



For peer review only - http://bmjopen.bmj.com/site/about/guidelines.xhtml
